# The hydroxamate based HDAC inhibitor WMJ-J-09 induces colorectal cancer cell death by targeting tubulin and downregulating survivin

**DOI:** 10.1038/s41598-025-04714-w

**Published:** 2025-06-04

**Authors:** Yu-Han Huang, Yu-Min Huang, Wei-Jan Huang, Meng-Chieh Yu, Chin-Hui Chuang, Ya-Fen Hsu, Hsiu-Chen Chen, Liang-Chieh Chen, Shiu-Wen Huang, Ming-Jen Hsu

**Affiliations:** 1 Division of Genetics and Genomics, Department of Pediatrics, Boston Children’s Hospital, Boston, MA USA; 2 Department of Surgery, School of Medicine, College of Medicine, Taipei Medical University, Taipei, Taiwan; 3 Division of Gastrointestinal Surgery, Department of Surgery, Taipei Medical University Hospital, Taipei Medical University, Taipei, Taiwan; 4 Department of Pharmaceutical Sciences, Taipei Medical University, Taipei, Taiwan; 5 Department of Pharmacology, School of Medicine, College of Medicine, Taipei Medical University, Taipei, Taiwan; 6 Graduate Institute of Medical Sciences, College of Medicine, Taipei Medical University, Taipei, Taiwan; 7 Division of General Surgery, Department of Surgery, Landseed Hospital, Taoyuan, Taiwan; 8 School of Medicine, National Sun Yat-Sen University, Kaohsiung, Taiwan; 9 Research Center of Thoracic Medicine, Taipei Medical University Hospital, Taipei, Taiwan; 10 Cell Physiology and Molecular Image Research Center, Wan Fang Hospital, Taipei Medical University, Taipei, Taiwan

**Keywords:** Acetylation, Histone deacetylase (HDAC), Liver kinase B1 (LKB1), P53, Survivin, Biochemistry, Drug discovery and development, Pharmacology

## Abstract

Aberrant expression of histone deacetylases (HDACs) is associated with cancer drug resistance and tumor progression. While considerable studies and effort have been devoted to developing novel HDAC inhibitors in cancer therapy, hydroxamate-based HDAC inhibitors have gained growing interest for their broad-spectrum anti-tumor properties. We developed a series of HDAC inhibitors featuring a hydroxamate moiety, and WMJ-J-09 was selected due to its potent cytotoxic effect in colorectal cancer (CRC) cells, and its molecular mechanisms driving CRC cell death were characterized. WMJ-J-09 reduced cell viability, arrested the cell cycle at the G2/M phase, and triggered apoptosis. Mechanistically, it activated LKB1-p38MAPK signaling, leading to p53 phosphorylation and acetylation, which elevated p21 and suppressed survivin levels. WMJ-J-09 also acetylated α-tubulin, impaired microtubule assembly, and acetylated survivin, resulting in proteasomal degradation. Both LKB1 siRNA and anacardic acid, a histone acetyltransferase inhibitor, reversed WMJ-J-09-reduced survivin, confirming its dual effects on survivin at transcriptional and post-translational levels. In vivo, the subcutaneous growth of HCT116 CRC xenografts was reduced by WMJ-J-09. In conclusion, WMJ-J-09 causes CRC cell death via the LKB1-p53-survivin signaling pathway and HDAC inhibition, leading to acetylation of α-tubulin, p53, and survivin. This study highlights WMJ-J-09’s potential as a promising therapeutic candidate for CRC treatment.

**Supplementary Information:**

The online version contains supplementary material available at 10.1038/s41598-025-04714-w.

## Introduction

Data from the World Health Organization (WHO) indicates that colorectal cancer (CRC) ranks third among the most commonly diagnosed malignancies and the second leading cause of cancer mortality globally, leading to a substantial impact on global healthcare expenditure ^1^. Despite advancements in screening and therapeutic interventions, as well as encompassing biological target therapy and immunotherapy, a significant proportion of CRC patients are diagnosed at advanced disease stages, and approximately 50% of them succumb due to drug resistance, relapse, or metastasis ^2,3^. Hence, continuously pursuing novel therapeutic agents or strategies is imperative to improve CRC survival rates. The multifaceted etiology of CRC involves environmental, genetic, and epigenetic factors. It appears that extensive epigenetic alterations, along with other genetic abnormalities, contribute to cancer initiation and progression ^4^. Epigenetic modifications, in contrast to genetic alterations in cancer, are reversible. These changes can be utilized as biomarkers for developing cancer therapy. Numerous literature reports the overexpression of histone deacetylase (HDAC), a class of epigenetic enzymes, in various cancers, including CRC. HDACs play a pivotal role in malignancy-associated epigenetic alterations ^5^. Therefore, HDAC inhibitors have emerged as promising entities for improving the therapeutic outcomes of CRC ^6^. While considerable efforts have been contributed to developing novel HDAC inhibitors for cancer treatment, hydroxamate-based HDAC inhibitors have gained growing attention due to their broad-spectrum anti-tumor activities ^7–10^. Among five U.S. Food and Drug Administration (FDA)-approved HDAC inhibitors in treating hematological malignancies, three are hydroxamate-based compounds, including vorinostat, belinostat, and panobinostat ^11^. Abexinostat, another hydroxamate derivative, is presently in clinical trials and demonstrates inhibitory activities against tumors ^12^. These observations imply that HDAC inhibitors with hydroxamate functional groups might exhibit anti-tumor properties with significant therapeutic potential, which warrants further exploration.

Survivin, an inhibitor of apoptosis (IAP) family member, is a distinctive protein characterized by different expression patterns in normal versus cancerous cells. Survivin is not detectable in differentiated adult tissues except for hematopoietic or vascular endothelial cells ^13^. Survivin overexpression, however, is commonly observed in most human malignancies, including CRC, contributing to its association with increased invasion, metastasis, and unfavorable patient prognosis ^14–16^. Survivin is recognized as a key regulator in modulating apoptosis, cell division, angiogenesis, and lymphangiogenesis, underscoring its significance as a potential therapeutic target ^7,17–19^. Preclinical investigations and experimental models have highlighted the promise of targeting survivin, demonstrating its potential to inhibit cancer cell growth and augment the effectiveness of conventional treatment modalities ^8,9,19,20^. Consequently, pharmacological targeting of survivin represents a noteworthy therapeutic strategy in CRC treatment.

Multiple mechanisms could regulate survivin gene expression, including transcriptional, post-translational, and post-transcriptional processes. Transcriptional regulation involves interactions of various transcription factors, including hypoxia-inducible factor-1α (HIF-1α), NF-κB, specificity protein 1 (Sp1), signal transducer and activator of transcription 3 (STAT3), as well as p53, with specific binding sequences in the promoter region of survivin ^7–9^. In addition, microRNAs (miRNAs) contribute to post-transcriptional regulation by targeting survivin mRNA for degradation or suppressing its translation. Furthermore, ubiquitination, phosphorylation, or acetylation, the post-translational modifications, can modulate the stability and function of the survivin protein ^17,18^. These mechanisms likely represent feasible options for targeting survivin in cancer treatment. Various strategies, such as small-molecule inhibitors, RNA interference, and immunotherapeutic approaches, are currently under investigation to modulate the survivin expression or function in CRC cells ^21^. To develop novel anti-tumor HDAC inhibitors, WMJ-J compounds, a series of HDAC inhibitors with an aliphatic hydroxamate group ^22^, were synthesized and evaluated for their anti-CRC properties. WMJ-J-09, among these, exhibited pronounced effects in promoting CRC cell death (Supplement Fig. 1). The present study explores the mechanisms underlying the CRC cell death induced by WMJ-J-09. Potential effects of WMJ-J-09 on survivin expression and key factors were also investigated.

**Fig. 1:**
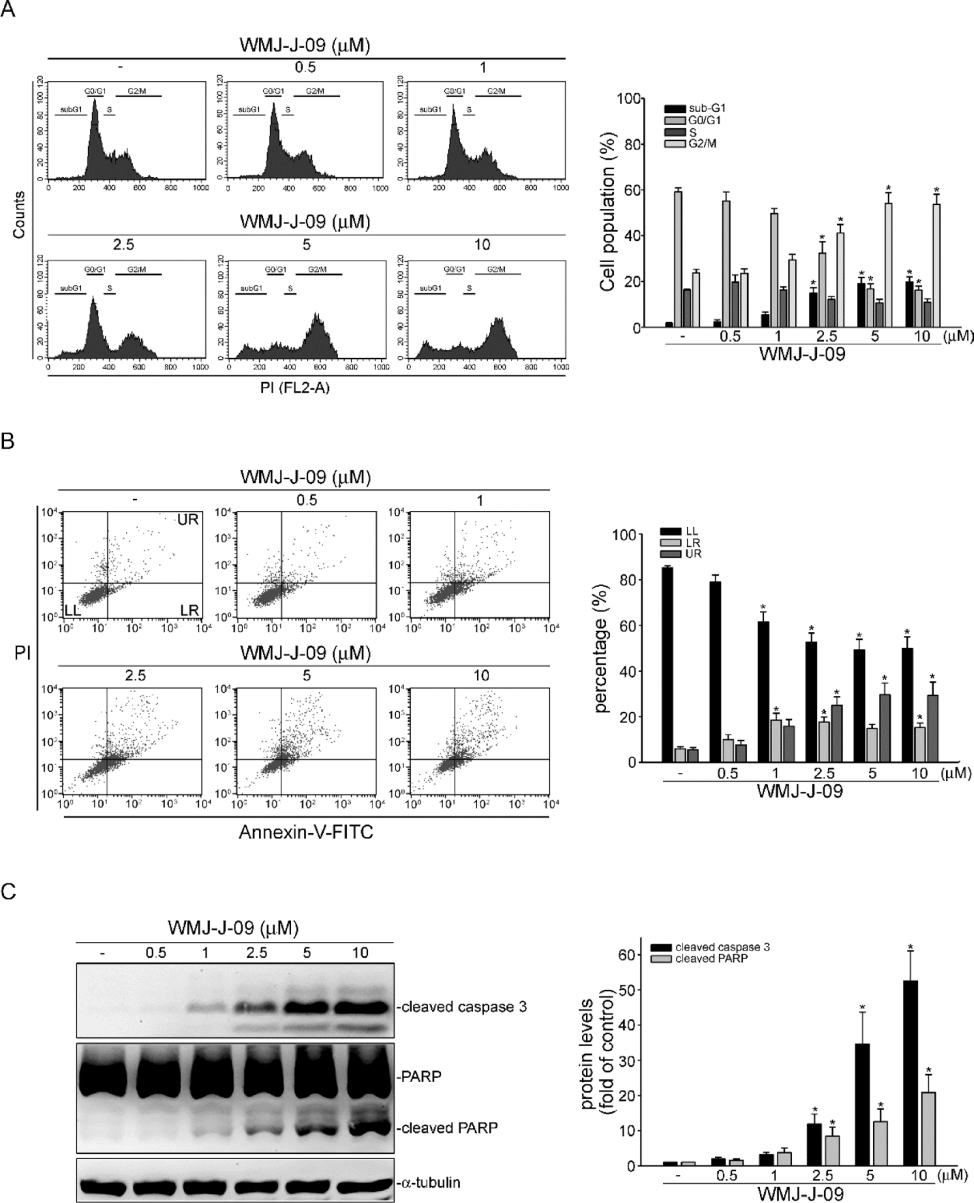
WMJ-J-09 arrested the cell cycle and induced apoptosis. (**A**) Flow cytometric analysis of WMJ-J-09-mediated cell cycle distribution in HCT116 cells using propidium iodide (P.I.) staining (left panel). The cell population of each phase was calculated in the right panel for each condition. (**B**) Flow cytometric analysis of WMJ-J-09-induced cell apoptosis using double labeling of annexin V and P.I. (left panel). The percentage of early (lower right, L.R.) and late (upper right, U.R.) apoptotic cells, as well as viable cells (lower left, L.L.), were calculated in the right panel. (**C**) Immunoblot results of PARP and caspase 3 cleavage in WMJ-J-09-treated HCT116 cells (left panel). Each band intensity was quantified, and cleaved caspase 3 and PARP fold changes were normalized by total α-tubulin levels (right panel). Error bars, mean ± S.E.M. (shown only for independent replicate experiments with n = 4). One-way ANOVA followed by Tukey’s post-hoc test assessed statistical significance (*p < 0.05 compared to the control group).

## Results

### WMJ-J-09 arrested the HCT116 cell cycle and triggered apoptosis

The WMJ-J compounds’ ^22^ effects on cell viability were first investigated via MTT assay on colorectal cancer (CRC) cell lines, including HCT15, HT29, Lovo, and HCT116. Most WMJ-J compounds significantly reduced cell viability in these CRC cell lines at the concentration of 10 µM after 24 to 48 h exposure (Supplement Fig. S1). Among these compounds, WMJ-J-09 notably reduced cell viability across multiple CRC cell lines and exhibited the most potent cytotoxic effects. While further validation in additional CRC cell lines is necessary for broader generalization, the consistent cytotoxic trend supports WMJ-J-09 as a potential therapeutic lead. We prioritized HCT116 cells for mechanistic investigations due to their well-characterized genetic background and frequent use in studies of apoptosis and cell cycle regulation. Further MTT assay results indicated that WMJ-J-09 concentration- and time-dependently reduced HCT116 cell viability after 24 or 48 h exposures (Supplement Fig. S2A). In contrast, WMJ-J-09 did not significantly affect cell viability in non-tumor FHC colon epithelial cells (Supplement Fig. S2B). These observations suggest that WMJ-J-09 may target cancer cells but not normal human cells. Consequently, we aimed to elucidate how WMJ-J-09 causes HCT116 cell death, possibly through inducing apoptosis or modulating cell cycle progression. We performed propidium iodide (PI) labeling and flow cytometric analysis to investigate the distribution change of the cell cycle phase in the presence of WMJ-J-09 treatment. A 24-h treatment of WMJ-J-09 reduced the cell distribution in the S phase, accompanied by a marked increase in the G2/M and apoptotic (sub-G1) phases (Fig. 1A). Additionally, double labeling with PI and annexin V-FITC was utilized to examine different sub-populations of apoptotic HCT116 cells after WMJ-J-09 exposure. As shown in Fig. 1B, the percentage of early apoptotic cells (LR; annexin V^+^PI^-^) and advanced apoptotic or necrotic cells (UR; annexin V^+^PI^+^) significantly increased after 24-h exposure to WMJ-J-09. Furthermore, WMJ-J-09 treatment led to increasing levels of caspase 3 cleavage as well as its selective substrate, poly ADP-ribose polymerase (PARP) (Fig. 1C). These results indicate that WMJ-J-09 arrests the cell cycle at G2/M phase and induces apoptosis in HCT116 CRC cells.

### Microtubule assembly disruption and increased α-tubulin acetylation in HCT116 cells after WMJ-J-09 exposure

**Fig. 2:**
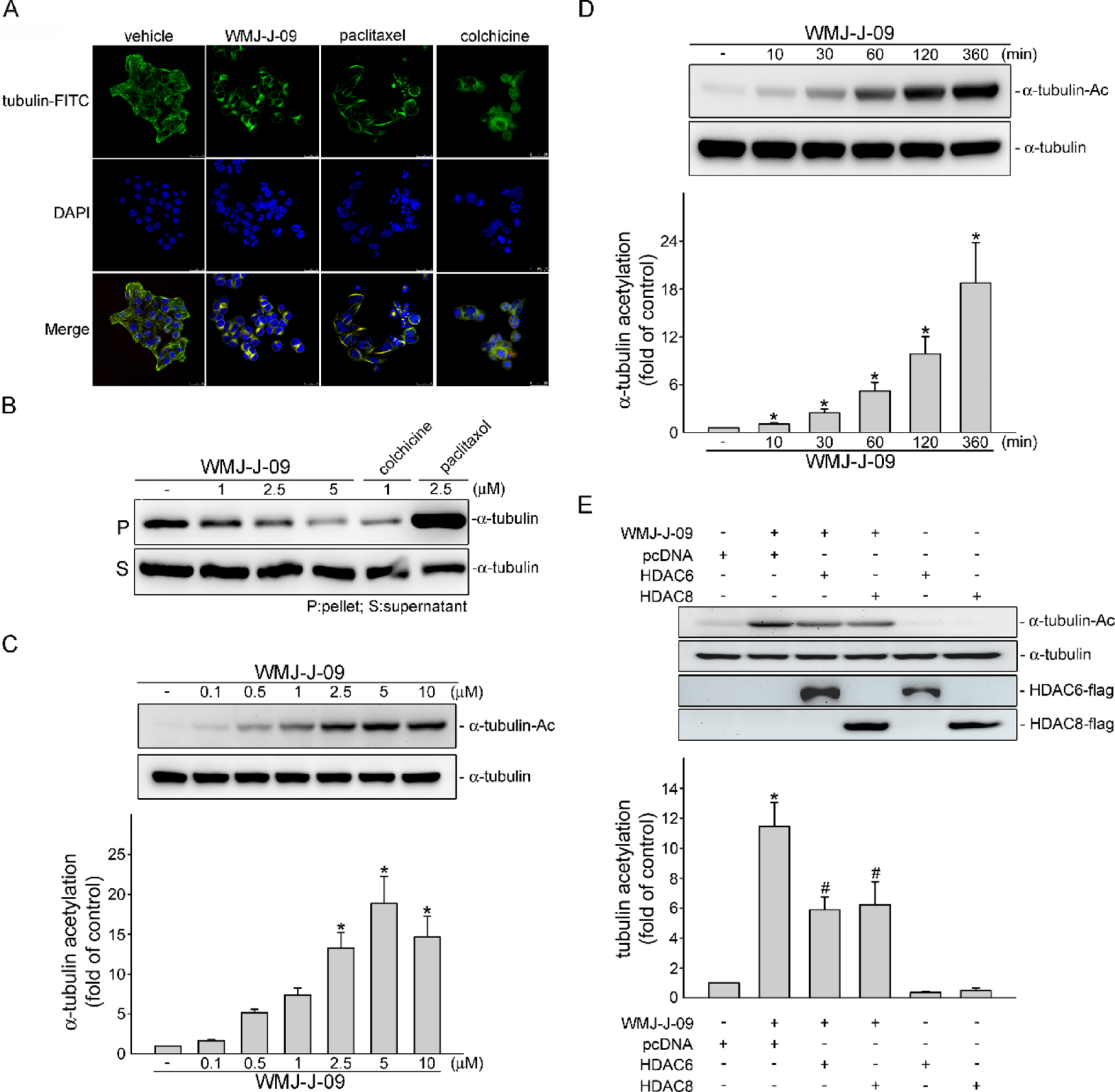
WMJ-J-09 disrupted microtubule assembly through HDAC inhibition. (**A**) Microtubule formation in HCT116 cells treated with colchicine, paclitaxel, WMJ-J-09, or vehicle, detected by confocal immunofluorescence analysis with β-tubulin-staining images in the upper panel, DAPI-staining images in the middle panel, and merged images in the bottom panel. (**B**) Immunoblotting result of polymerized tubulin in HCT116 cells treated with colchicine, paclitaxel, WMJ-J-09, or vehicle (**C**, **D**) Immunoblot result of α-tubulin acetylation induced by WMJ-J-09 in either a concentration-dependent (**C**) or time-dependent manner (**D**). (**E**) Immunoblot result of α-tubulin acetylation in WMJ-J-09-treated HCT116 cells with HDAC6 or HDAC8 overexpression. Each band intensity was quantified, and the total α-tubulin level normalized the fold changes of α-tubulin acetylation. Error bars, mean ± S.E.M. (shown only for independent replicate experiments with n ≥ 5).

Microtubule dynamics, consisting of α- and β-tubulin, are crucial in the cell cycle transition between mitosis and interphase, and the cell cycle G2/M arrest is often accompanied by the disruption in tubulin distribution ^23^. Two microtubule-targeting agents, colchicine and paclitaxel, were utilized as reference compounds to determine the action of WMJ-J-09 on tubulin distribution associated with the cell cycle G2/M arrest in HCT116 cells. Colchicine, an anti-mitotic drug, inhibits the intercellular microtubule assembly and polymerization, while paclitaxel is a microtubule-stabilizing agent inducing microtubule polymerization ^24^. Under the confocal immunofluorescence microscopy using specific β-tubulin antibodies, WMJ-J-09-treated cells showed the disappearance of the normal distribution of tubulin (Fig. 2A), comparable to the cells treated with colchicine rather than paclitaxel, indicating cellular microtubule depolymerization. Next, the contents as soluble (monomeric) or polymeric tubulin in HCT116 cell lysate were analyzed via western blot analysis. Paclitaxel, as expected, increased the polymeric tubulin in the precipitate and pellet fractions, while colchicine and WMJ-J-09 significantly reduced the polymerized tubulin fraction (Fig. 2B).

Tubulin could undergo post-translational modifications, including acetylation that alters microtubule dynamics and disrupts the assembly ^8,22,25^. In addition, WMJ-J-09 was revealed to have inhibitory activity toward HDACs in HCT116 cells, as evidenced by increasing levels of histone 3 (H3) acetylation (Supplement Fig. S3). We also compared WMJ-J-09’s impact on HCT116 cell viability with the pan-HDAC inhibitor suberoylanilide hydroxamic acid (SAHA, vorinostat) using the MTT assay. Both compounds exhibited similar potency in reducing HCT116 viability (Supplement Fig. S4A). However, in p53-null HCT116 cells, WMJ-J-09 demonstrated better efficacy than SAHA (Supplement Fig. S4B). To further characterize the HDAC inhibitory profile of WMJ-J-09, we evaluated its in vitro enzyme inhibition activity against a panel of recombinant human HDAC isoforms. As shown in Table 1, WMJ-J-09 exhibited potent inhibitory activity against class I HDACs, including HDAC1 (IC_50_ = 7.5 ± 0.7 nM), HDAC2 (IC_50_= 21.3 ± 1.3 nM), HDAC3 (IC_50_ = 18.4 ± 3.0 nM), and HDAC8 (IC_50_ = 90.9 ± 14.7 nM). Notably, WMJ-J-09 also strongly inhibited HDAC6 (class IIb) with an IC_50_ value of 3.9 ± 1.0 nM, while showing moderate inhibition toward HDAC4 (class IIa) with an IC_50_ of 8715.7 ± 645.8 nM. Compared to SAHA, WMJ-J-09 displayed stronger inhibition of most HDAC isoforms tested, particularly HDAC1 and HDAC6. These results suggest that WMJ-J-09 possesses broad-spectrum HDAC inhibitory activity with potential as a pan-HDAC inhibitor. Hence, we examined whether the HDAC inhibitory effect of WMJ-J-09 would also modulate α-tubulin acetylation. As shown in Fig. 2C and Fig. 2D, α-tubulin acetylation increased concentration- and time-dependently after exposure to WMJ-J-09. Furthermore, HCT116 cells overexpressed with HDAC8 (a class I HDAC) or HDAC6 (a class II HDAC) showed significant attenuation in α-tubulin acetylation elicited by WMJ-J-09 (Fig. 2E). These observations indicate that WMJ-J-09 may inhibit selective HDAC to increase α-tubulin acetylation and this subsequently interferes the microtubule assembly in HCT116 cells thereby cell cycle arrest.

**Table 1:** In vitro inhibitory activity (IC_50_ values*, nM) of WMJ-J-09 and SAHA against recombinant human HDAC isoforms.

Compound	Class I				Class IIa	Class IIb
	HDAC1	HDAC2	HDAC3	HDAC8	HDAC4	HDAC6
WMJ-J-09	7.5 ± 0.7	21.3 ± 1.3	18.4 ± 3.0	90.9 ± 14.7	8715.7 ± 645.8	3.9 ± 1.0
SAHA	38.9 ± 6.7	153.9 ± 8.8	51.5 ± 2.3	1413.2 ± 155.6	> 10,000	9.3 ± 0.6

*IC₅₀ values represent the mean ± SD of three independent experiments.

### p53 participates in WMJ-J-09-caused alterations in survivin and p21 levels in HCT116 cells

**Fig. 3:**
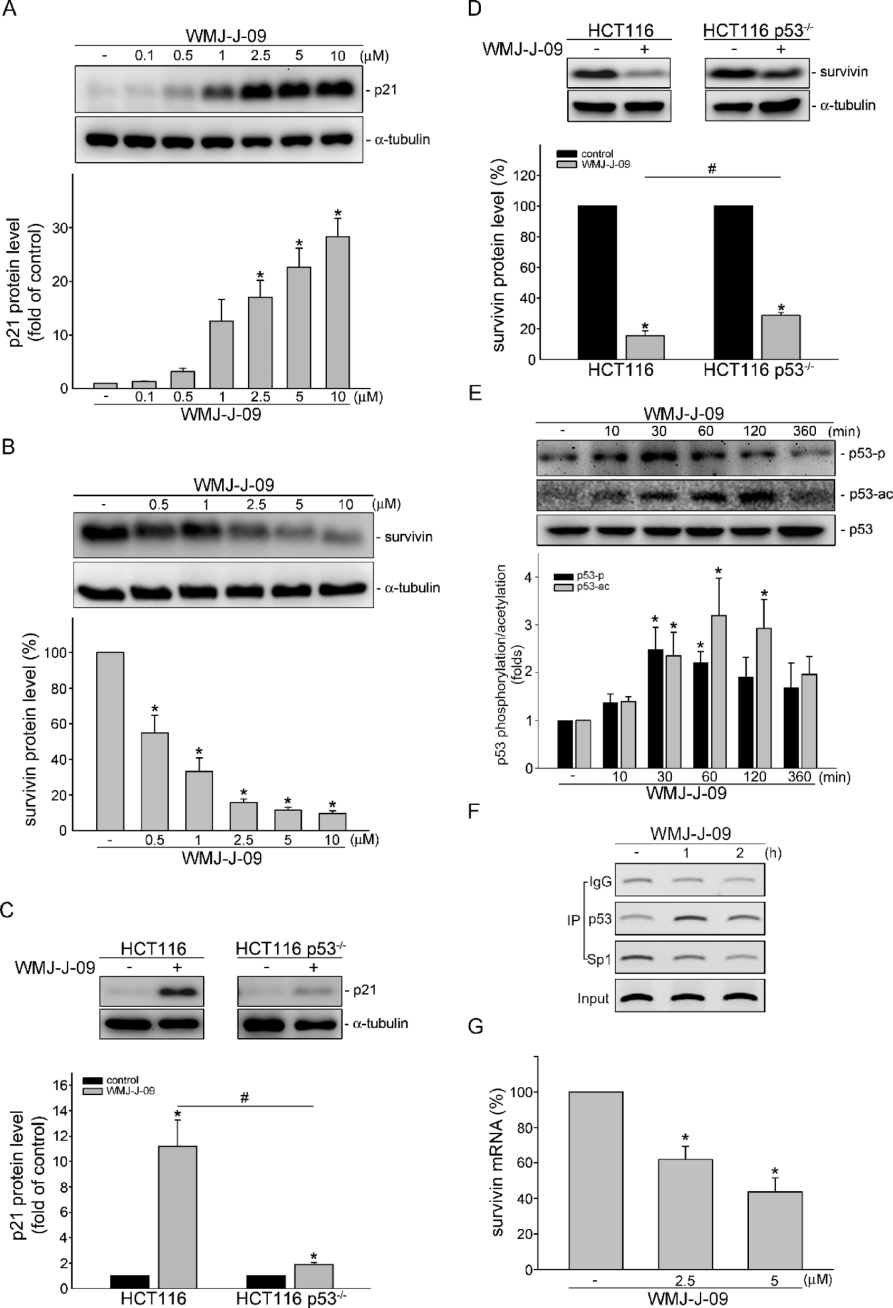
p53 participates in WMJ-J-09-mediated p21 expression and survivin reduction. (**A**, **B**) Immunoblot result of WMJ-J-09-mediated p21 (A) and survivin (B) expression in HCT116 cells. (**C**, **D**) Immunoblot result of WMJ-J-09-mediated p21 (**C**) and survivin (**D**) expression in p53 wildtype (HCT116) and p53 deficient (HCT116 p53^-/-^) HCT116 cells. (**E**) Immunoblot result of p53 phosphorylation and acetylation induced by WMJ-J-09 in HCT116 cells. Each band intensity was quantified, and total α-tubulin levels normalized the fold changes of p21 and survivin; total p53 levels normalized the fold changes of p53 phosphorylation and acetylation. (**F**) Cells were treated with WMJ-J-09 (5 μM) for 1 or 2 h. ChIP assay was performed as described in the “Methods” Section. Typical traces representative of three independent experiments with similar results are shown. (**G**) RT-qPCR result of WMJ-J-09-mediated survivin mRNA downregulation. Error bars, mean ± S.E.M. (shown only for independent replicate experiments with n ≥ 4). One-way ANOVA followed by Tukey’s post-hoc test assessed statistical significance (*p < 0.05 compared to the control group).

Survivin, as an IAP, also functions as a key cell cycle regulator. Generally, survivin is expressed highly in the G2/M phase and rapidly declines in the G1 phase ^19,26^. In contrast, survivin reduction results in the G2/M cell cycle arrest and apoptosis in various cancers, including CRC ^8,22,27^. Furthermore, p21, a cyclin-dependent kinase (CDK) 1 inhibitor, contributes to G2 cell cycle arrest ^28^. Therefore, we investigated the effect of WMJ-J-09 on p21 and survivin expression in HCT116 cells. As demonstrated in Fig. 3A, WMJ-J-09 treatment significantly increased the p21 protein levels, whereas it decreased the survivin protein levels (Fig. 3B). These observations indicate that WMJ-J-09-caused HCT116 cell death may involve p21 elevation and survivin suppression.

In response to various stress signals, p53, the tumor suppressor gene, could activate the p21 promoter, resulting in the upregulation of p21 expression ^29^. Concurrently, p53 could counteract transcription factor Sp1 binding to the survivin promoter region, thereby repressing survivin expression ^7,27^. To explore the role of p53 in regulating WMJ-J-09-mediated p21 and survivin expression, we utilized HCT116 cells and their isogenic derivatives with the p53 gene knocked out (HCT116 p53^-/-^). As shown in Fig. 3C, WMJ-J-09-induced p21 expression was significantly abolished in HCT116 p53^-/-^ cells. Similarly, compared to HCT116 cells, the survivin reduction via WMJ-J-09 was less pronounced in HCT116 p53^-/-^ cells (Fig. 3D). Post-translational modification of p53, especially acetylation, is crucial for its stability and transcriptional activity in response to stresses ^30^. Given that WMJ-J-09 is a pan-HDAC inhibitor, which could induce tubulin acetylation as described above, the effect of WMJ-J-09 on the acetylation of p53 was first investigated. As shown in Fig. 3E, WMJ-J-09 treatment induced p53 acetylation time-dependently. At the same time, p53 phosphorylation was also examined, which is also responsible for its transcriptional activity in response to stress signals ^31^. Figure 3E shows that WMJ-J-09 also induced the phosphorylation of p53 in a time-dependent manner. These suggested that WMJ-J-09 enhances p53 activity, potentially interfering with Sp1 binding to the survivin promotor. To investigate this, a ChIP assay was performed. As shown in Fig. 3F, WMJ-J-09 treatment increased p53 binding to the *survivin* promoter region (-264 to -37), while Sp1 binding was concurrently reduced. The impact of WMJ-J-09 on survivin gene expression was examined. As shown in Fig. 3G, the mRNA level of survivin was significantly reduced in HCT116 cells exposed to WMJ-J-09, suggesting WMJ-J-09 repressed the survivin expression. These results suggest that WMJ-J-09 could activate p53 by enhancing its acetylation and phosphorylation, thereby inducing p21 expression and suppressing survivin, leading to apoptosis and cell cycle arrest in HCT116 cells.

### LKB1-p38MAPK signaling pathway is involved in WMJ-J-09’s actions on p53 and survivin in HCT116 cells

**Fig. 4:**
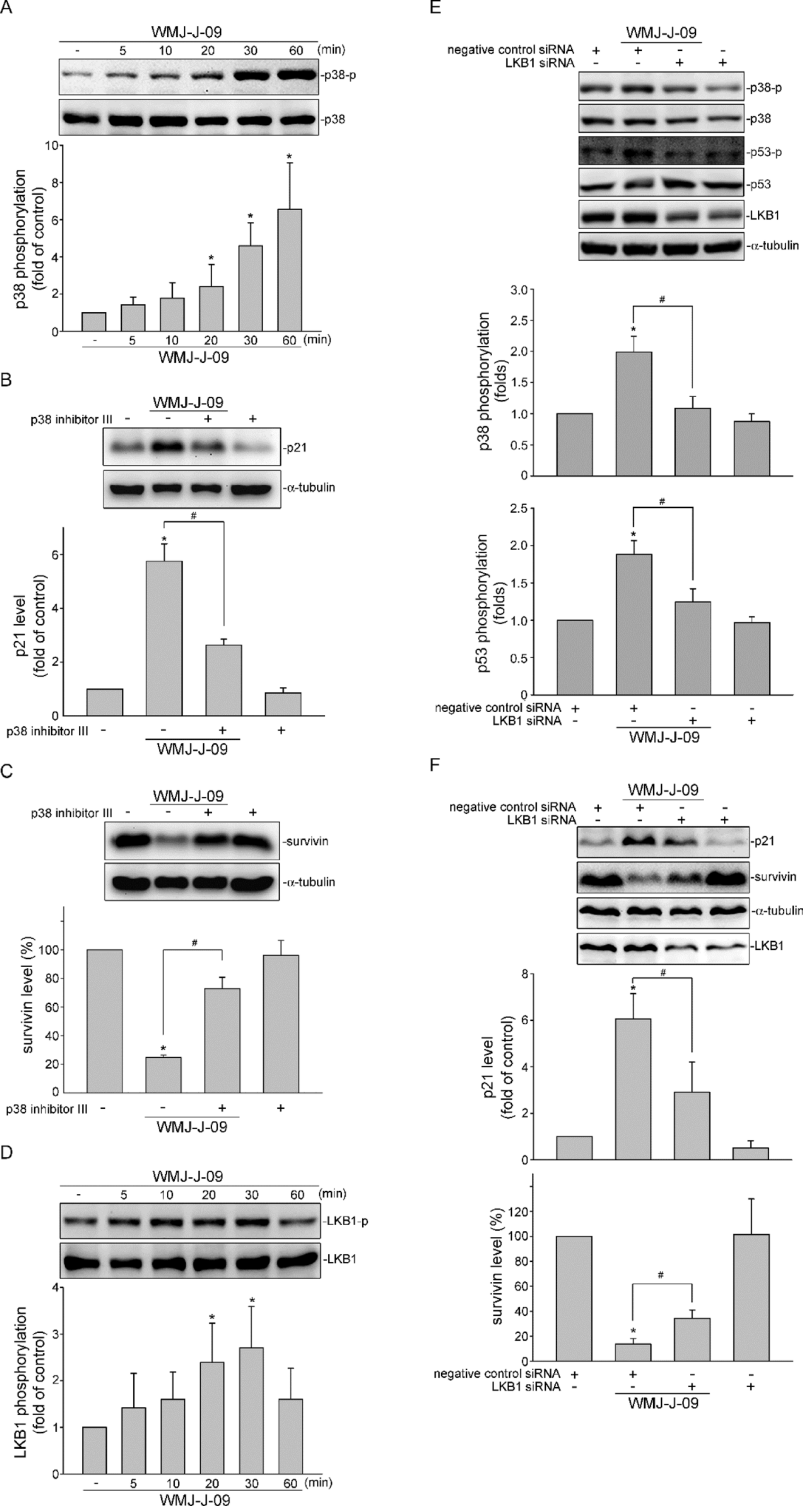
LKB1-p38 MAPK signaling contributed to WMJ-J-09-mediated p21 and survivin regulation. (**A**) Immunoblot result of p38MAPK phosphorylation in HCT116 cells exposed to WMJ-J-09. (**B**, **C**) Immunoblot result of p21 (**B**) and survivin (**C**) expression in WMJ-J-09-stimulated HCT116 cells with or without p38 inhibitor III (**D**) Immunoblot result of LKB1 phosphorylation in HCT116 cells exposed to WMJ-J-09 for indicated periods. (**E**) Immunoblot results from the effects of LKB1 siRNA or negative control siRNA on p38MAPK and p53 phosphorylation elicited by WMJ-J-09. (**F**) Immunoblot result of the effects of LKB1 siRNA or negative control siRNA on WMJ-J-09-modulated p21 and survivin expression in HCT116 cells. Each band intensity was quantified, and total α-tubulin levels normalized the fold changes of LKB1, p21, and survivin; total p53 levels normalized that of p53 phosphorylation; total p38MAPK levels normalized that of p38MAPK phosphorylation; total LKB1 levels normalized that of LKB1 phosphorylation. Error bars, mean ± S.E.M. (shown only for independent replicate experiments with n ≥ 4). One-way ANOVA followed by Tukey’s post-hoc test assessed statistical significance (*p < 0.05 compared to the control group).

WMJ-J-09, as an HDAC inhibitor, not only promotes p53 acetylation but also induces its phosphorylation. The protein kinase p38MAPK is crucial in regulating cellular responses, mainly by arresting the cell cycle and inducing cell death via p53 phosphorylation ^10,32,33^. The effect of WMJ-J-09 on p38MAPK was examined in HCT116 cells. WMJ-J-09 treatment time-dependently led to p38MAPK phosphorylation (Fig. 4A). The pharmacological inhibition of p38MAPK with p38MAPK inhibitor III markedly attenuated the WMJ-J-09-elevated p21 expression (Fig. 4B). It also reversed the WMJ-J-09-induced survivin suppression (Fig. 4C). Liver kinase B1 (LKB1), a serine/threonine kinase known for its role as a master kinase in cellular energy sensing and homeostasis, also acts as a tumor suppressor by activating its downstream kinases such as AMPK and p38MAPK ^22,34^. Therefore, we explored whether LKB1 contributes to p38MAPK activation and consequential events in HCT116 cells following WMJ-J-09 treatment. A significant increase in LKB1 phosphorylation was noted in WMJ-J-09-stimulated HCT116 cells (Fig. 4D). knockdown of LKB1 using siRNA suppressed p38MAPK and p53 phosphorylation caused by WMJ-J-09 (Fig. 4E). Additionally, LKB1 siRNA reduced the actions of WMJ-J-09 on p21 induction and survivin suppression (Fig. 4F). These findings suggest that LKB1-p38MAPK-p53 signaling pathway triggered by WMJ-J-09 leads to increased p21 expression and survivin reduction, culminating in HCT116 cell death.

### Survivin degradation was enhanced in WMJ-J-09-stimulated HCT116 cells

**Fig. 5:**
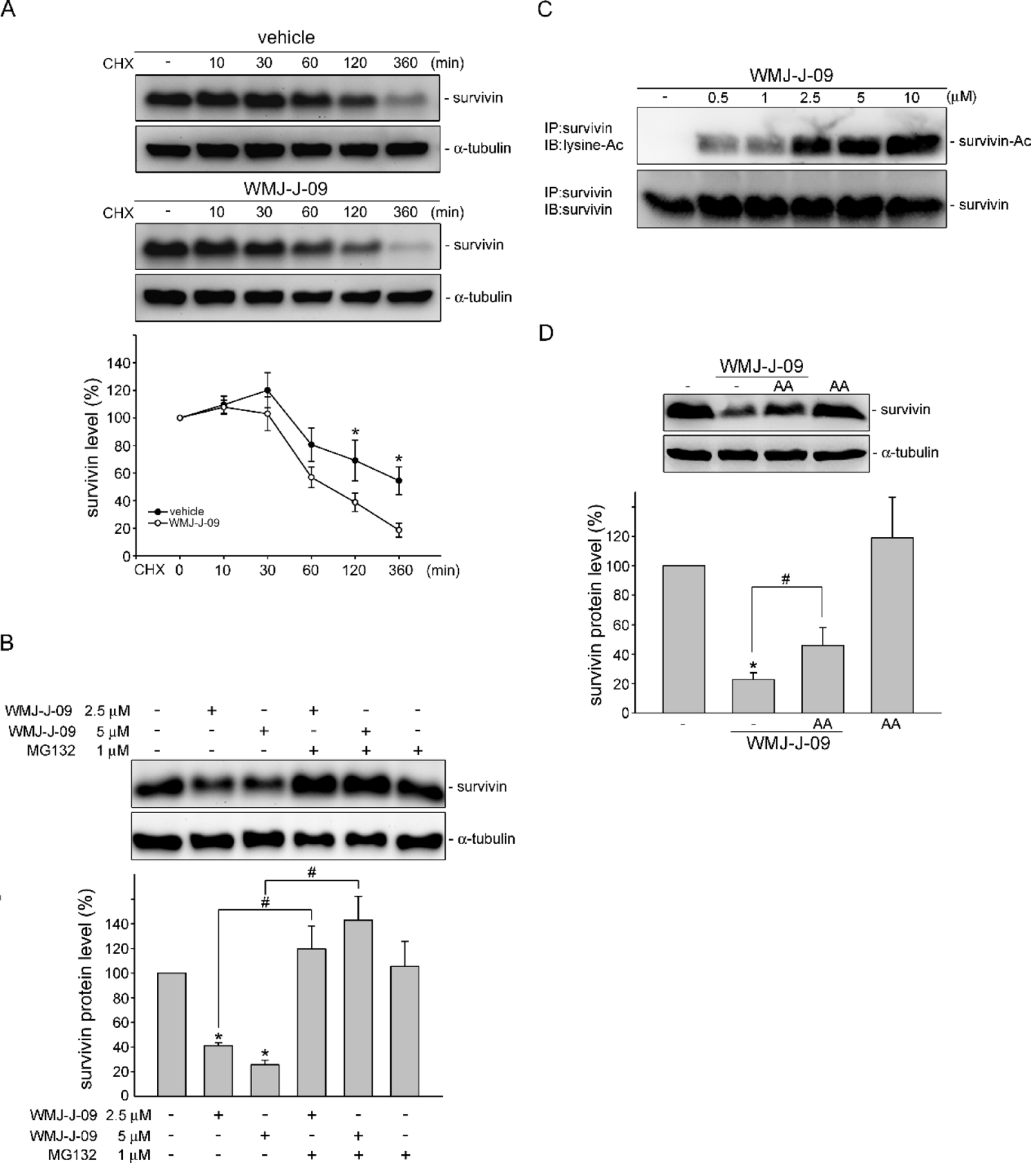
WMJ-J-09 induced survivin degradation. (**A**) Immunoblot result of the survivin protein stability in WMJ-J-09-treated HCT116 cells. (**B**) Immunoblot result of WMJ-J-09-induced proteasomal degradation of survivin. (**C**) Immunoprecipitation and immunoblot result of the survivin acetylation in WMJ-J-09-treated HCT116 cells. (**D**) Immunoblot result of the survivin protein in WMJ-J-09-stimulated HCT116 cells with vehicle or anacardic acid. Each band intensity was quantified, and total α-tubulin levels normalized the fold changes of survivin and its acetylation. Error bars, mean ± S.E.M. (shown only for independent replicate experiments with n ≥ 4). One-way ANOVA followed by Tukey’s post-hoc test assessed statistical significance (*p < 0.05 compared to the control group). CHX, cycloheximide; IP, immunoprecipitation; IB, immunoblotting; Ac, acetylation.

Survivin can be post-translationally acetylated to mediate protein stability ^35–37^. It showed that most class I/II HDACs are crucial in regulating survivin acetylation, and pharmacological inhibition of HDACs could induce survivin protein degradation via the proteasomal degradation pathway ^35–37^. Therefore, we questioned whether the inhibitory effect of WMJ-J-09 on HDACs could also impact survivin acetylation and, as expected, exert survivin protein degradation. To do so, we first explored whether WMJ-J-09 would affect the survivin protein stability via cycloheximide (CHX) chase assay. It was indicated that in the presence of WMJ-J-09, the degradation of survivin protein was accelerated (Fig. 5A). In addition, the proteasomal inhibitor MG132 could significantly reverse the survivin degradation induced by WMJ-J-09 (Fig. 5B). Next, we performed immunoprecipitation and immunoblotting analysis to explore whether the alteration in the survivin protein stability was modulated by post-translational acetylation. Figure 5C showed that WMJ-J-09 significantly induced the acetylation on survivin (Fig. 5C). In addition, WMJ-J-09-mediated survivin reduction could be attenuated by adding a histone acetyltransferase (HAT) inhibitor, anacardic acid (Fig. 5D). In summary, the results support that WMJ-J-09 could promote the acetylation on the survivin protein and it undergoes proteasomal-dependent protein degradation, which eventually contributes to cell death in HCT116 cells.

### The growth of the HCT116 tumor xenograft was reduced by WMJ-J-09

**Fig. 6:**
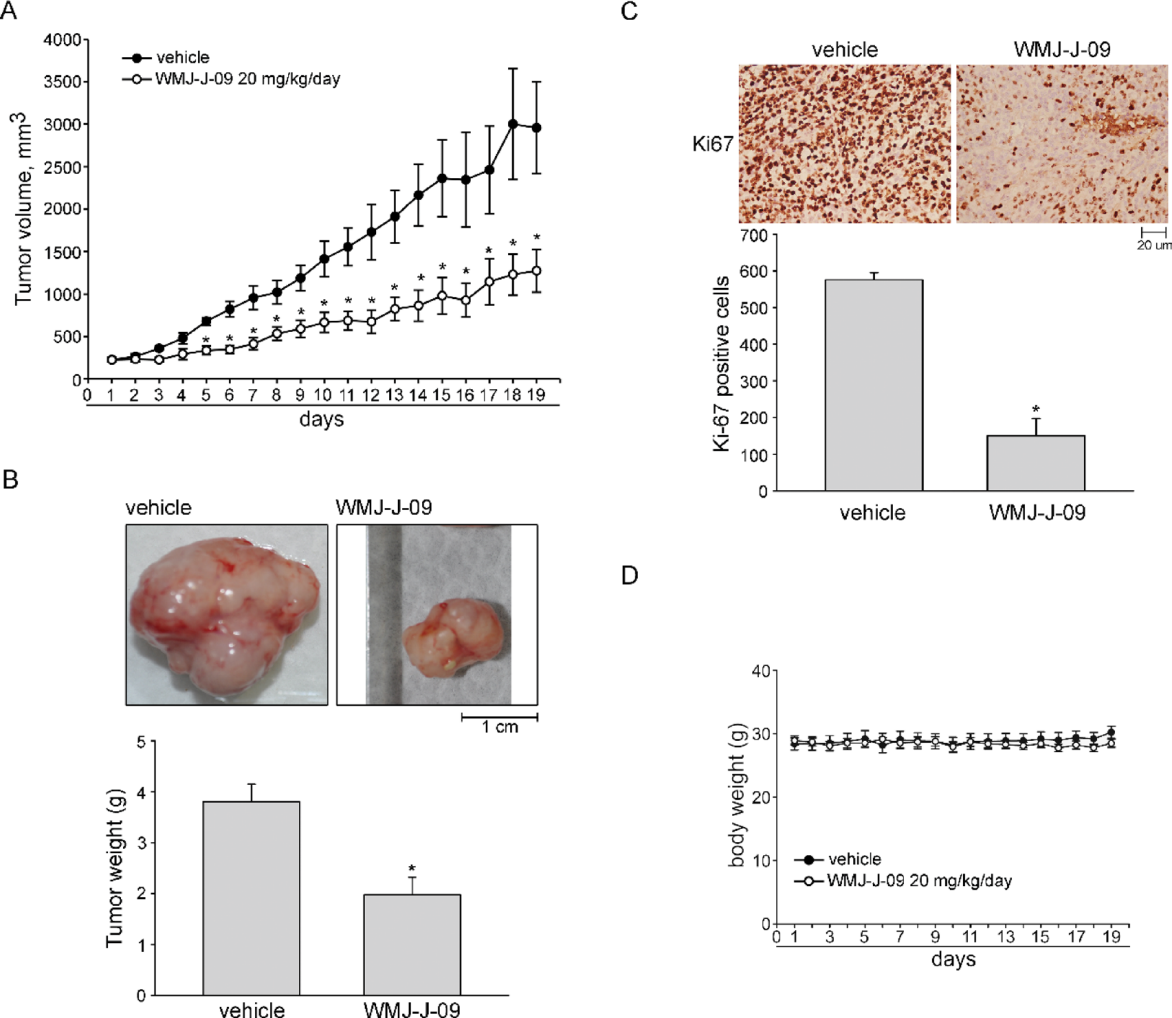
WMJ-J-09 inhibited in vivo HCT116 tumor xenograft growth. (**A**) Tumor growth in mice administrated with or without WMJ-J-09. (**B**) Tumor imaging and weight measurement excised from mice administrated with or without WMJ-J-09. (**C**) Ki-67 positive cells in tumors excised from mice administrated with or without WMJ-J-09. (**D**) Body weight in mice administrated with or without WMJ-J-09. Error bars represent mean ± S.E.M. (shown only for independent replicate experiments with n = 6). One-way ANOVA followed by Tukey’s post-hoc test assessed statistical significance (*p < 0.05 compared to the control group).

**Fig. 7:**
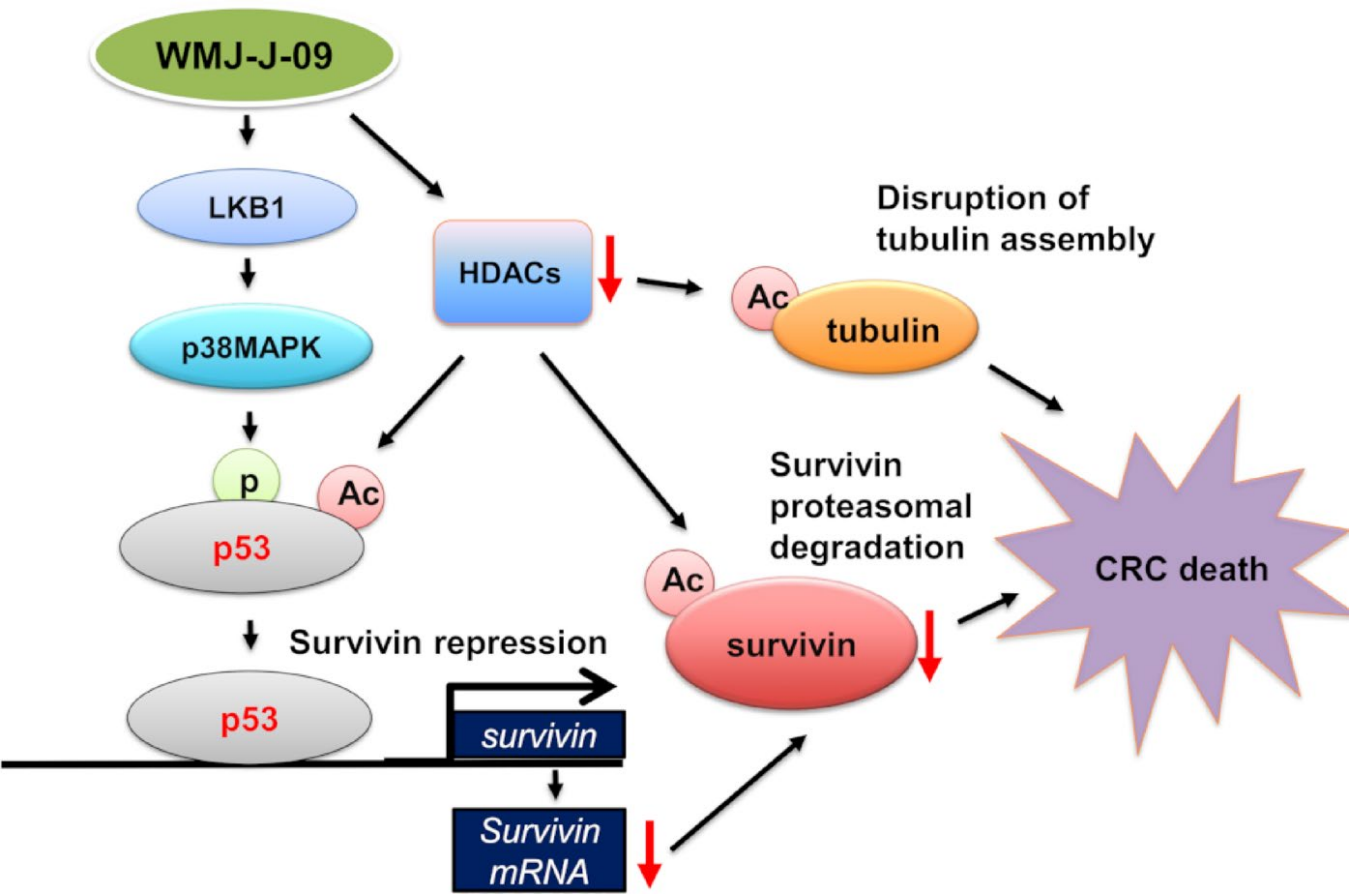
Schematic representation of the signaling mechanism underlying WMJ-J-09-induced colorectal cancer cell death.

Whether WMJ-J-09 is effective against CRC in vivo was examined via the murine xenograft model. HCT116 cells were injected into each nude mouse’s right flank subcutaneously. Once tumors reached an approximate volume of 200 mm^3^, mice were intraperitoneally administered either 20 mg/kg/day WMJ-J-09 or vehicle for 19 days. Upon completion of the treatment period, the xenografts were collected after the mice were euthanized. As shown in Fig. 6A, WMJ-J-09 markedly inhibited HCT116 tumor xenograft growth in vivo (Fig. 6A). WMJ-J-09 treatment also markedly reduced tumor weight (Fig. 6B) compared to the vehicle-treated controls. We further assessed cell proliferation in the tumors by immunohistochemistry (IHC) staining for the nuclear antigen Ki67, a marker of cellular proliferation. The number of Ki67-positive cells was markedly reduced in WMJ-J-09-treated xenografts compared to vehicle-treated tumors (Fig. 6C), indicating reduced cell proliferation. Additionally, treatment with WMJ-J-09 at the concentration of 20 mg/kg/day did not impact mouse body weight over the 19 days compared to the control group (Fig. 6D). It indicates that WMJ-J-09 effectively reduces in vivo tumor growth. Together, these findings support the notion that WMJ-J-09 induces CRC cell death through the LKB1-p53-survivin signaling pathway and HDAC inhibition, leading to the acetylation of α-tubulin, p53 and survivin, ultimately resulting in survivin downregulation (Fig. 7).

## Discussion

The ongoing development of new strategies and treatments for CRC highlights the advancements and the remaining challenges in the field ^38^. While traditional cytotoxic chemotherapeutics remain the most established and effective regimens, targeted therapies and immunotherapies have marked the beginning of a new era of precision medicine. The success of monoclonal antibodies targeting vascular endothelial growth factor (VEGF) and epidermal growth factor receptor (EGFR) signaling, as well as the advent of immune checkpoint inhibitors, has significantly improved therapeutic outcomes in CRC patients ^39^. Beyond these targeted approaches, exploring HDAC inhibitors’ potential for treating solid tumors, including CRC, is promising, while most approved HDAC inhibitors are mainly valuable for treating hematological tumors ^40,41^. Hydroxamate-based HDAC inhibitors have shown promising results in preclinical and early clinical studies ^42^. However, their precise mechanisms and clinical efficacy in CRC are still being investigated. The present study revealed that WMJ-J-09, an innovative aliphatic hydroxamate-based HDAC inhibitor, enhances the acetylation of α-tubulin, p53, and survivin. On the other hand, WMJ-J-09 also triggers the LKB1-p38MAPK-p53 signaling pathway, resulting in p21 elevation and survivin depression at the transcriptional level. Together with its effects on protein acetylation and transcriptional regulation, these eventually lead to apoptosis and G2/M phase arrest in HCT116 CRC cells. Moreover, WMJ-J-09 showed high efficiency in suppressing HCT116 xenograft growth in vivo.

WMJ-J-09 shows consistency with prior studies that hydroxamate derivatives elicit p21 expression and arrest cell cycle in different cancer cells ^8,9,22,43^. WMJ-J-09 was also indicated with survivin suppression in HCT116 CRC cells. The actions of WMJ-J-09 on survivin and p21 are alleviated in HCT116 p53^-/-^ cells, suggesting p53’s causal role in mediating the anti-tumor activities of WMJ-J-09. Post-translational modifications (PTMs), including acetylation and phosphorylation, are the primary mechanism in regulating p53 stabilization and activation ^31,44,45^, and WMJ-J-09 promotes both p53 acetylation and phosphorylation. WMJ-J-09 increased p53 Ser15 phosphorylation via the LKB1-p38MAPK signaling pathway. In addition to p38MAPK, AMPK, a downstream signaling mediator of LKB1, can also lead to p53 phosphorylation at Ser20 in the apoptotic stress ^9,22,32,46,47^. At the same time, AMPK can also promote p38MAPK activation in stress-signaling pathways ^48^. Our recent studies showed that WMJ-J-09 time-dependently increased AMPK phosphorylation (Supplement Fig. S5A). WMJ-J-009’s impacts on p38MAPK phosphorylation (Supplement Fig. S5B), survivin, and p21 (Supplement Fig. S5C) were reduced in HCT116 cells transfected with AMPK dominant negative mutant (DN). This evidence raises the possibility that AMPK may mediate LKB1-activated p38MAPK and p53 in HCT116 cells in the presence of WMJ-J-09. Further studies must clarify whether WMJ-J-09 could activate AMPK-mediated p53 phosphorylation at Ser20 or other residues.

Beyond promoting p53 phosphorylation, WMJ-J-09, as a pan-HDAC inhibitor, also induced the acetylation of p53 and α-tubulin by inhibiting HDAC. This indicates that WMJ-J-09 mediates p53 activation, phosphorylation, and acetylation via the LKB1-p38MAPK signaling and HDAC inhibition. The possible crosslink between the two mechanisms remains to be established in HCT116 cells. Previously, Wang et al. ^49^ reported that HDAC6 knockdown in macrophages could lead to microtubule acetylation, subsequently amplifying the p38MAPK signaling and consequential events in macrophages. This evidence may imply that tubulin acetylation induced by WMJ-J-09 may disrupt microtubule assembly and activate the p38MAPK-p53 cascade in HCT116 cells. The transient increase in p53 acetylation and phosphorylation by WMJ-J-09 likely reflects a dynamic regulatory mechanism. Similar oscillatory dynamic behaviors in p53 PTM have been reported, such as p53 acetylation changes in breast cancer cells treated with sirtuin 1 (SIRT1) inhibitor^50^ and p53 phosphorylation fluctuation in lymphoblastoid cells after irradiation^51^. These reversible modifications may involve SIRT1-mediated deacetylation^50^ or phosphatase-driven dephosphorylation^51,52^. Additionally, the pharmacokinetics of WMJ-J-09 may influence the transient nature of p53 activation. Importantly, despite its transient effects, a single treatment of WMJ-J-09 was sufficient to activate downstream signaling, ultimately inducing CRC death. This suggests that the observed PTM changes are functionally relevant to its cytotoxic effects. Future studies will explore additional signaling pathways that may contribute to p53 regulation in this context.

Additionally, WMJ-J-09 showed a partial restoration in survivin reduction in p53 deficient HCT116 cells, and this may also imply the involvement of transcription factors other than p53 in CRC cell death. We previously demonstrated that p63 mediates survivin downregulation and consequent p53-deficient hypopharyngeal tumor cell death ^22^. p63, as a member of the p53 family, may also cooperate with p53 in counteracting Sp1 binding to the survivin promoter region and repressing its expression in HCT116 cells ^27^. p53 was predominantly investigated in the HCT116 cell line since it is a wild-type p53 cell line, and p53 plays a vital role in the apoptotic paradigm. Whether p63 or other transcription factors contribute to survivin depression induced by WMJ-J-09 in the HCT116 cell line is worth investigating further. We noted that WMJ-J-09 significantly reduces cell viability in p53-deficient HCT116 cells more efficiently than SAHA (Supplement Fig. S4B), suggesting that it may retain anti-CRC activity in tumors with p53 deficiency, a common feature in CRC and a known factor in chemoresistance. It indicates that WMJ-J-09 not only functions as an effective HDAC inhibitor but may also have advantages over existing pan-HDAC inhibitors in targeting p53-deficient or chemoresistant CRC cells. Future studies will further explore its therapeutic potential in resistant CRC models.

Survivin represents an attractive target for cancer treatment due to its critical role in therapy resistance and cancer cell survival ^18,53^. A decrease in survivin expression arrests the cell cycle and induces apoptosis in various cancers, including CRC ^27,54^. Previous studies have shown that HDAC inhibition can promote survivin acetylation, increasing nuclear translocation and reducing protein stability ^17^. SAHA, a well-characterized HDAC inhibitor, has been shown to enhance survivin acetylation and facilitate its degradation through the proteasomal pathway ^36^. In line with these findings, we observed that WMJ-J-09 enhances survivin acetylation and facilitates its degradation. We also noted that the HAT inhibitor anacardic acid reverses the effect of WMJ-J-09 on survivin suppression**,** further supporting the role of acetylation in this process. Further investigations are needed to explore survivin subcellular localization upon WMJ-J-09 treatment and the potential contribution of subcellular localization changes**.** Furthermore, WMJ-J-09 can suppress survivin transcription by enhancing the activity of its upstream repressor, p53. This partially explains that survivin reduction by WMJ-J-09 was less pronounced in p53- deficient HCT116 cells. Both protein acetylation and ubiquitination are post-translational modifications on lysine residues. The crosslink between the two modifications may contribute to protein stabilization and degradation regulation. Trichostatin A and valproic acid, two HDAC inhibitors, are known to affect the ubiquitination state in substrates, including accelerating protein degradation ^55^. Moreover, it is reported that survivin acetylation is responsible for its nuclear localization ^17^, and the nuclear survivin remains ubiquitinated and could undergo ubiquitin-directed proteolysis ^56,57^. Therefore, it is worth investigating if survivin acetylation induced by WMJ-J-09 will be further ubiquitinated to undergo proteasomal degradation in HCT116 cells.

The possible mediator interplaying in-between is also worth exploring further, given that WMJ-J-09 shows its action on survivin suppression in both transcriptional and post-translational levels. Hsp90, as a heat shock protein chaperone, is highly associated with the correct folding of survivin protein and subsequently prevents it from proteasomal degradation ^58^. At the same time, Hsp90 also impacts the transcriptional, as well as post-transcriptional modification, of the survivin expression ^59^. Hsp90 activation, on the other hand, is highly regulated by the acetylation modification via HDAC6 as the main deacetylase of Hsp90 ^60^. Hence, the impact of WMJ-J-09 on survivin expression in both transcriptional and post-translational levels could also be attributed to Hsp90 regulation via inhibiting HDAC6.

We noted that treatment with WMJ-J-09 at 20 mg/kg/day for 19 days did not result in significant body weight loss in mice compared to the control group (Fig. 6D), indicating that the compound is well-tolerated at this dosage. Additionally, WMJ-J-09 did not affect the cell viability of non-tumor FHC colon epithelial cells (Supplement Fig. S2B). This suggests that WMJ-J-09 preferentially targets malignant cells, sparing normal human colon epithelium, which is a promising feature for potential therapeutic applications. Moreover, the reduction in HCT116 cell viability induced by 5-fluorouracil (5-FU) (Supplement Fig. S6A) or oxaliplatin (Supplement Fig. S6B) was significantly greater in the presence of WMJ-J-09, as determined by the MTT assay. These findings suggest that WMJ-J-09 may act synergistically with existing CRC treatments, potentially improving therapeutic outcomes and reducing the required doses of conventional chemotherapy, thereby minimizing associated toxicities. Future investigations will be necessary to further explore its pharmacokinetics, toxicity, and the mechanisms underlying its synergy with conventional treatments, as well as to assess its in vivo therapeutic efficacy in CRC models.

It is recognized that prolonged exposure to HDAC inhibitors can lead to resistance in cancer cells ^61,62^, and understanding whether CRC cells develop resistance to WMJ-J-09 is an important aspect for future investigation. While our current study focuses on the immediate effects of WMJ-J-09 on CRC cells, future studies are needed to assess the potential resistance mechanisms by evaluating long-term WMJ-J-09 exposure in CRC cells, monitoring changes in drug sensitivity, and identifying potential adaptive mechanisms, such as alterations in epigenetic regulators, compensatory signaling pathways, or drug efflux transporters. Additionally, we will investigate whether combinatorial strategies (e.g., WMJ-J-09 with other targeted therapies) could help mitigate resistance development.

In conclusion, this study highlights WMJ-J-09, a novel HDAC inhibitor featuring with hydroxamate moiety, as a promising candidate for anti-tumor agent development. Elucidating its underlying mechanisms may provide valuable insights into the therapeutic potential of hydroxamate-based HDAC inhibitors against CRC. Further investigations are needed to elucidate the underlying mechanisms and optimize their clinical translation.

## Methods

### Reagents

Colchicine, p38MAPK inhibitor III, paclitaxel, MG132, anacardic acid, cycloheximide, and propidium iodide were purchased from MedChemExpress (Monmouth Junction, NJ, U.S.A). Turbofect™ transfection reagent, Fetal bovine serum (F.B.S.), TrypLE™, Opti-MEM™, all cell culture reagents including RPMI1640, Ham’s F12, and DMEM medium were from Thermo Fisher Scientific (Waltham, MA, U.S.A.). Anti-rabbit and anti-mouse IgG conjugated horseradish peroxidase antibodies and antibodies against DDDDK (Flag) and α-tubulin were from GeneTex Inc (Irvine, CA, U.S.A.). McCoy’s 5A medium, MTT, negative siRNA, and LKB1 siRNA were from Sigma-Aldrich (St Louis, MO, U.S.A). Normal IgG and anti-p21 antibody were from Santa Cruz Biotechnology (Santa Cruz, CA, U.S.A.). Antibodies against α-tubulin Lys40 acetylation, p53, p53 Lys379 acetylation, p53 Ser15 phosphorylation, p38MAPK Thr180/Tyr182 phosphorylation, p38MAPK, LKB1, LKB1 Ser428 phosphorylation, PARP, cleaved caspase 3 (active form) and survivin were from Cell Signaling (Danvers, MA, U.S.A.). The chemiluminescent H.R.P. substrate kit was from Millipore (Billerica, MA, U.S.A.). We obtained all materials for western blot analysis from Bio-Rad (Hercules, CA, U.S.A.). pcDNA-HDAC6-FLAG was a gift from Tso-Pang Yao (Addgene plasmid # 30482; http://n2t.net/addgene:30482; RRID:Addgene_30482) ^63^. HDAC8 Flag was a gift from Eric Verdin (Addgene plasmid # 13825; http://n2t.net/addgene:13825; RRID:Addgene_13825) ^64^. All other chemicals used in this study were from Sigma-Aldrich (St Louis, MO, U.S.A.).

### The WMJ-J series of HDAC inhibitors synthesis

The WMJ-J series of HDAC inhibitors featuring an aliphatic hydroxamate moiety, including WMJ-J-09, was synthesized as a previously reported method ^22^.

### Cell culture

Dr. Bert Vogelstein kindly provided the HCT116 cells and their isogenic derivatives with the p53 gene knocked out (HCT116 p53^-/-^) ^65^. Other cell lines used in this study, including HCT-15, LoVo, and HT29, were from the Bioresource Collection and Research Center (BBRC, Hsinchu, Taiwan). These cells were maintained in RPMI1640 (HCT-15), Ham’s F12 (LoVo) or DMEM (HT29), McCoy’s 5A (HCT116), containing penicillin G (100 U/ml), streptomycin (100 μg/ml), and FCS (10%) in a humidified 37 °C incubator.

### Flowcytometry

#### Propidium iodide (PI) staining

Following the indicated treatments, cells were collected and fixed in 70% ethanol for 24 h at 0 °C. After washing with phosphate-citric acid buffer, the fixed cells were stained using a staining solution containing PI (25 μg/ml), RNase A (100 μg/ml), and Triton X-100 (0.1%) for 30 min in the dark. We performed flow cytometric analysis with the FACScan and Cellquest program (BD Biosciences, San Jose, CA, U.S.A). To examine the percentage of PI-stained cells in each cell cycle phase, we utilized the ModFit program (BD Biosciences, San Jose, CA, U.S.A) as described previously ^9^.

#### Double staining with PI and annexin V-FITC

Following the indicated treatments, cells were harvested and incubated immediately in the dark at 37 °C with a staining solution containing annexin V-FITC (2 μg/ml) and PI (25 μg/ml) for 20 min. We performed flow cytometric analysis with the FACScan and Cellquest program (BD Biosciences, San Jose, CA, U.S.A). We utilized the FCS Express program (BD Biosciences, San Jose, CA, U.S.A) to examine the percentage of stained cells in different quadrants as described previously ^9^. Each panel’s upper right (U.R.) quadrant indicates advanced apoptotic and necrotic cells (annexin V^+^PI^+^). The lower right (L.R.) quadrant corresponds to early apoptotic cells (annexin V^+^PI^-^), while the lower left (L.L.) quadrant represents viable cells (annexin V^-^PI^-^).

### Immunoblotting (western blot analysis)

Following the indicated treatments, a lysis buffer containing NP-40 (0.5%), Tris (10 mM) (pH 7.0), PMSF (2 mM), NaCl (140 mM), leupeptin (0.2 mM), and pepstatin A (0.05 mM) was utilized to lyse cells to extract proteins. SDS-PAGE was utilized to separate protein samples in equal quantities, then transferred to a nitrocellulose membrane (Pall Corporation, Washington, NY, U.S.A.). Following transfer and 1-h blocking by incubation with TBST buffer containing 5% non-fat, the proteins immobilized on the membranes undergo primary antibody hybridization. Secondary antibodies conjugated with horseradish peroxidase were used to detect target proteins. Immunoreactivity was detected using the Western chemiluminescent HRP substrate kit, as per the manufacturer’s recommendations. We used a densitometer integrated into a scientific imaging system (Biospectrum AC System, UVP) to obtain quantitative data.

### Transfection in HCT116 cells

We performed cell transfection as per the manufacturer’s recommendations using a Turbofect™ transfection reagent (Invitrogen, Carlsbad, CA, USA). HCT116 cells (7 × 10^4^ cells/well) were transfected with HDAC8-Flag, HDAC6-Flag, or pcDNA for western blot analysis. The suppression of the target gene was carried out as previously described^8^. Pre-designed siRNAs targeting human LKB1 (STK11, NM_000455) for LKB1 suppression and negative control (NC) siRNA were purchased from Sigma-Aldrich (St. Louis, MO, USA). The siRNA sequences were as follows: LKB1 siRNA, 5′-guacuucugucagcugauu-3′ (SASI_Hs01-00092687); negative control siRNA, 5′-gaucauacgugcgaucaga-3′ (41105324).

### Immuno-precipitation

Cells were lyzed in a lysis buffer [Triton X-100 (1%), NaCl (125 mM), MgCl_2_ (1 mM), PMSF (1 mM), sodium orthovanadate (100 μM), leupeptin (10 μg/ml), aprotinin (10 μg/ml), and Tris–HCl (20 mM) (pH 7.5)]. After 30 min centrifugation at 4 °C, the supernatant was collected and incubated with IgG or anti-survivin antibody at 4 °C with gentle rotation for 16 h in the presence of protein A-magnetic beads (Millipore, Billerica, MA, U.S.A.). Following three washes, the immunoprecipitated complexes were subjected to immunoblotting to assess the acetylation status of survivin.

### Immunofluorescence assay

We examined tubulin assembly as previously described ^8^. HCT116 cells were cultured on glass coverslips, followed by a 24-h treatment with paclitaxel, colchicine, or WMJ-J-09. Subsequently, the cells were washed with PBS twice and fixed with 4% paraformaldehyde at room temperature for 15 min. Cells were permeabilized by incubation with PBS containing Triton X-100 (0.1%) for 30 min. HCT116 cells underwent two PBS washes and were incubated with PBS containing 1% BSA for 1 h. The fixed cells on the coverslips were incubated with rabbit antibody against β-tubulin (1:100, Cell Signaling, Danvers, MA, U.S.A.) at 4 °C for 16 h. Following two additional washes, the fixed cells on the coverslips were incubated with anti-rabbit IgG conjugated with FITC for 1 h. A DAPI-containing mounting medium (SlowFad Gold, Thermo Fisher Scientific, Waltham, MA U.S.A.) was utilized to mount the coverslips. The distribution of tubulin was analyzed using a confocal microscope (Zeiss, LSM 410). Green fluorescence represented β-tubulin, while blue fluorescence (derived from DAPI) corresponds to nuclei.

### RT-qPCR

For complementary DNA (cDNA) synthesis, treated and untreated cells were collected to extract total RNA as previously described ^8^. We performed real-time PCR using a StepOne Real-Time PCR system (Applied Biosystems, Grand Island, NY, U.S.A.) with the GoTaq qPCR Master Mix (Promega, Madison, WI, U.S.A.). The PCR cycling protocol included an initial hot-start activation at 95 °C for 2 min, followed by 40 cycles of denaturation at 95 °C for 15 s, and annealing/extension at 60 °C for 60 s. The primer sequences used for human GAPDH and survivin transcripts were as follows: GAPDH reverse, 5′-agg ggtctacatggcaactg-3′; GAPDH forward, 5′-gtcagtggtggacctgac ct-3′; survivin reverse, 5′-aacccttcccagactccact-3′; survivin forward, 5′-gcctttccttaaaggccatc-3′.

### HDAC activity analysis

Effects of WMJ-J-09 on HDAC activity were examined using a fluorometric HDAC activity assay as described previously ^66^. Briefly, 10 μL of recombinant HDAC1, HDAC2, HDAC3, HDAC6 (BPS Biosciences, Huissen, The Netherlands), HDAC4, or HDAC8 ^67^ in HDAC buffer (15 mM Tris–HCl pH 8.1, 0.25 mM EDTA, 250 mM NaCl, 10% glycerol) as well as 50 μL of vehicle, WMJ-J-09 or SAHA at different concentrations were added to a well of a 96-well microtiter plate. After pre-incubation at 30 °C for 5 min, the enzymatic reaction was started by the addition of 40 μL substrate at 10 μM. These substrates include Boc-Lys(Ac)-AMC (Bachem, Bubendorf, Switzerland) for HDAC6; KI 177 (Enzo Life Science, Long Island, NY, U.S.A.) for HDAC1, HDAC 2, and HDAC3; Boc-Lys(TFA)-AMC (Bachem, Bubendorf, Switzerland) for HDAC4, and HDAC8. After incubation at 37 °C for 30 min, the reaction was stopped by adding 100 μL trypsin solution (10 mg/mL trypsin in 50 mM Tris–HCl pH 8, 100 mM NaCl). After incubation at 37 °C for another 20 min, fluorescence was measured (excitation λ = 355 nm, emission λ = 460 nm) with VICTOR X2 microplate spectrophotometer. The fluorescence in wells with vehicle only (0.1% DMSO, negative control) was set as 100% enzymatic activity, and the fluorescence in wells with thr enzyme eliminated was set as 0% enzymatic activity. The fluorescence ratio of WMJ-J-09 to negative control was defined as the percentage of remaining enzyme activity. The IC_50_ values were calculated by linear regression of the data. All experiments were performed in triplicate.

### Chromatin immunoprecipitation (ChIP) assay

A ChIP assay was performed as previously described ^9^. Briefly, cells were cross-linked with 1% formaldehyde at 37 °C for 10 min, rinsed with ice-cold PBS, and lysed in SDS buffer. Chromatin was sonicated (4 × 15 s) and centrifuged (10 min), and the supernatant was diluted in a ChIP dilution buffer. After pre-clearing with protein A-agarose (60 µl, 1 h, 4 °C), an aliquot of each sample was used as “input” in the PCR analysis. The remainder of the soluble chromatin was incubated at 4 °C overnight with control normal IgG, SP1, or p53 antibodies (Santa Cruz Biotechnology, Santa Cruz, CA, USA). Immunocomplexes were captured using protein A-agarose (20 µl, 2 h, 4 °C), followed by sequential washes in low-salt, high-salt, and LiCl immune complex washing buffers, and two washes in Tris–EDTA buffer. DNA was eluted, cross-links reversed (0.2 M NaCl, 65 °C, 4 h), and purified using GP™ DNA purification spin columns (Viogene, New Taipei City, Taiwan). PCR amplification of a 228-bp *survivin* promoter fragment (-264 to -37) was performed using Promega PCR Master Mix with primers, sense: 5’-ttc ttt gaa agc agt cga gg-3’ and antisense: 5’-tca aat ctg gcg gtt aat gg-3’. PCR conditions: 30 cycles (95 °C for 30 s, 56 °C for 30 s, 72 °C for 45 s). Products were analyzed by 1.5% agarose gel electrophoresis.

### Mouse xenograft model

We performed animal studies per the ARRIVE guidelines ^68,69^. We utilized nude_nu/nu_ mice to establish a xenograft model as previously described ^9^ to evaluate the in vivo anti-tumor efficacy of WMJ-J-09. Male nude_nu/nu_ mice (four-week-old), each weighing approximately 24 g, were procured from BioLasco (Taipei, Taiwan) for the experiments shown in Fig. 6. The mice were housed in groups of three per cage under specific pathogen-free (SPF) conditions, with a standard 12-h light/dark cycle at 22 °C at the Laboratory Animal Center of Taipei Medical University. They were provided with standard chow and autoclaved water. Upon transfer from BioLasco, vivarium staff randomly assigned the mice to individually ventilated cages (IVC) and acclimatized them in the animal housing facility for seven days before experimentation. Cells (HCT116) were collected, suspended in PBS at a density of 5 × 10^6^ cells in a 250 μl volume, and injected into each mouse’s flanks subcutaneously. When the tumors reached an approximate volume of 200 mm^3^, we randomly divided the mice into two groups: a treatment group (6 mice) and a control group (6 mice) that were intraperitoneally administered with 20 mg/kg/day WMJ-J-09 once daily for 19 days. Tumor size was measured daily using a digital caliper. We calculated the tumor volume with the formula *V* (mm3) = [*ab*2] × 0.52, where 'a' represents the tumor length and 'b' represents the width ^8,9,22^. Body weights were recorded daily throughout the 19-day treatment period. The animals were euthanized at the end of the treatment using carbon dioxide, and the tumors were excised and weighed. This study adhered to the Guide for the Care and Use of Laboratory Animals (NIH publication No. 85-23, revised 1996) and received approval from the Taipei Medical University Animal Care and Use Committee (Permit Number: LAC-2020-0451).

### Immunohistochemical analysis

The proliferative cells (Ki67^+^ area) in the cryosections obtained from HCT116 xenografts were determined using a rabbit anti-Ki67 antibody (Novus Biologicals, Littleton, CO, USA) and peroxidase-conjugated goat anti-rabbit antibody (The Jackson Laboratory, Sacramento, CA, USA). Stable diaminobenzidine was employed to visualize antibody binding. Images were obtained in four different quadrants of each tumor section at ×40 magnification. Measurement of the area of Ki67-stained proliferative cells was performed as described previously ^9^.

### Randomization and blinding

We used HCT116 cells to assess the actions of WMJ-J-09 compared to the control group in all cell experiments. Thus, formal randomization was not applied. In the animal model, vivarium staff randomly assigned mice to cages, and mice were subsequently randomized into WMJ-J-09- or vehicle-treated groups. Additionally, separate individuals conducted the experiments (operators) and analyzed the data (analysts) to ensure blinding.

### Data analysis

The data and statistical analyses in this study were conducted as described previously ^22^, following the guidelines for experimental design and analysis in pharmacology ^70^. Results are presented as mean ± standard error of the mean (S.E.M.) with n ≥ 5, where '*n*' denotes the number of independent values, not replicates. We applied normalization to control extraneous variation and highlight significant trends after the treatment. We performed statistical analyses utilizing SigmaPlot 14 (Build 10.0.0.54; Systat Software, San Jose, CA, U.S.A.). For non-parametric data, two groups were compared using the unpaired Student’s t-test, while the Mann–Whitney test was used for non-parametric data. For comparisons involving more than two groups, one-way analysis of variance (ANOVA) with Tukey’s posthoc test was used for parametric data, and the Kruskal–Wallis test, followed by Dunn’s multiple comparison tests, was applied for non-parametric data. A *P* value of less than 0.05 was considered statistically significant. We conducted post hoc tests only if the “F” value achieved P < 0.05 without significant inhomogeneity.

## Electronic supplementary material

Below is the link to the electronic supplementary material.

Supplementary Material 1

Supplementary Material 2

## Data Availability

The data supporting this study’s findings are available from the corresponding author upon reasonable request.

## Abbreviations

CRC: Colorectal cancer
HAT: Histone acetyltransferase
HDAC: Histone deacetylase
LKB1: Liver kinase B1
