# Supplementary material for: The hydroxamate based HDAC inhibitor WMJ-J-09 induces colorectal cancer cell death by targeting tubulin and downregulating survivin
Source: Sci Rep. 2025 Jun 4;15:19590. doi: 10.1038/s41598-025-04714-w (PMC12137728; doi:10.1038/s41598-025-04714-w)
Supplement: Supplementary file 1 — Supplementary Material 1 [file 41598_2025_4714_MOESM1_ESM.pptx]

## Slide 1
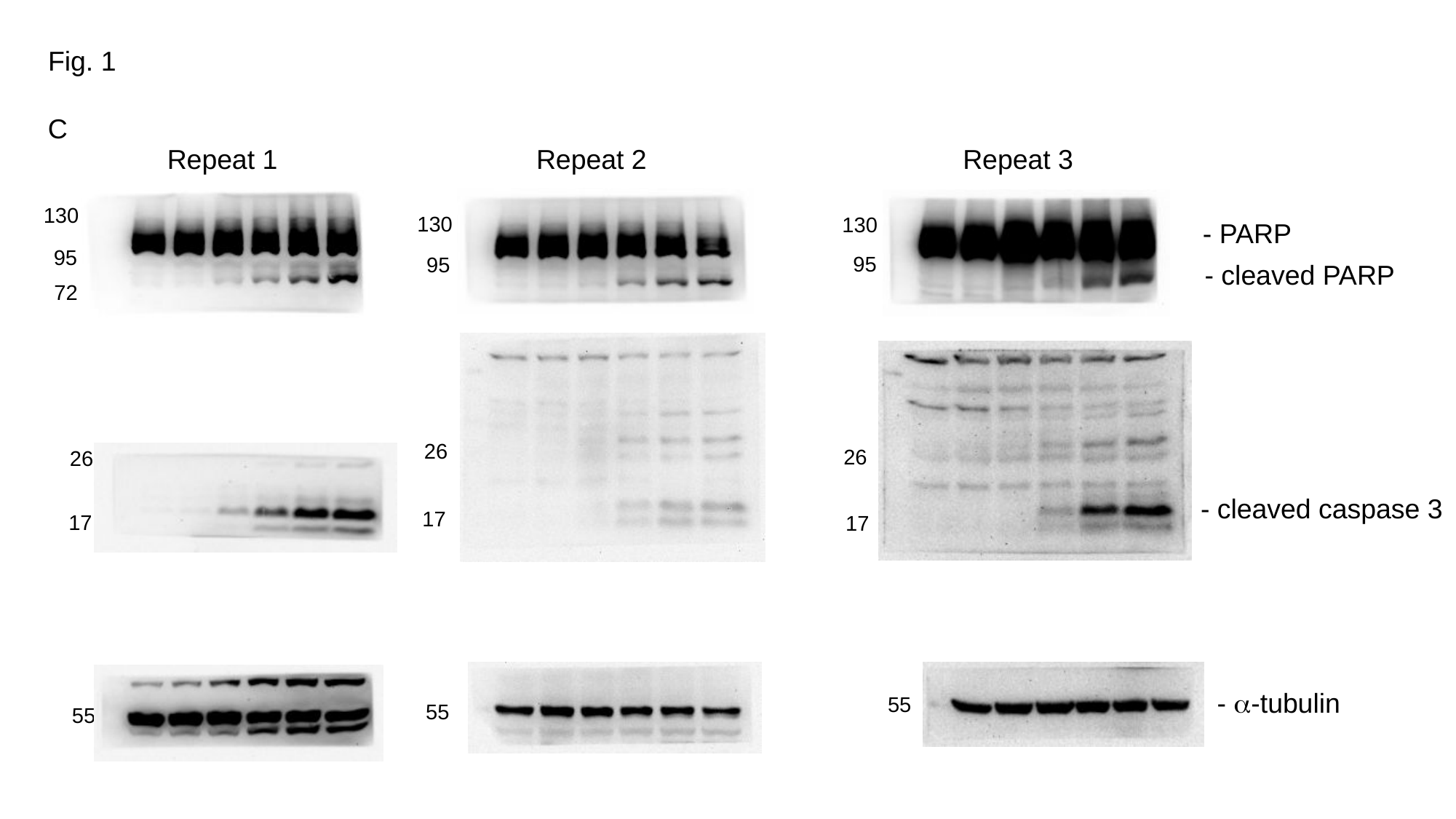

Fig. 1
C
Repeat 1
Repeat 2
Repeat 3
130
130
130
- PARP
95
95
95
- cleaved PARP
72
26
26
26
- cleaved caspase 3
17
17
17
- a-tubulin
55
55
55

## Slide 2
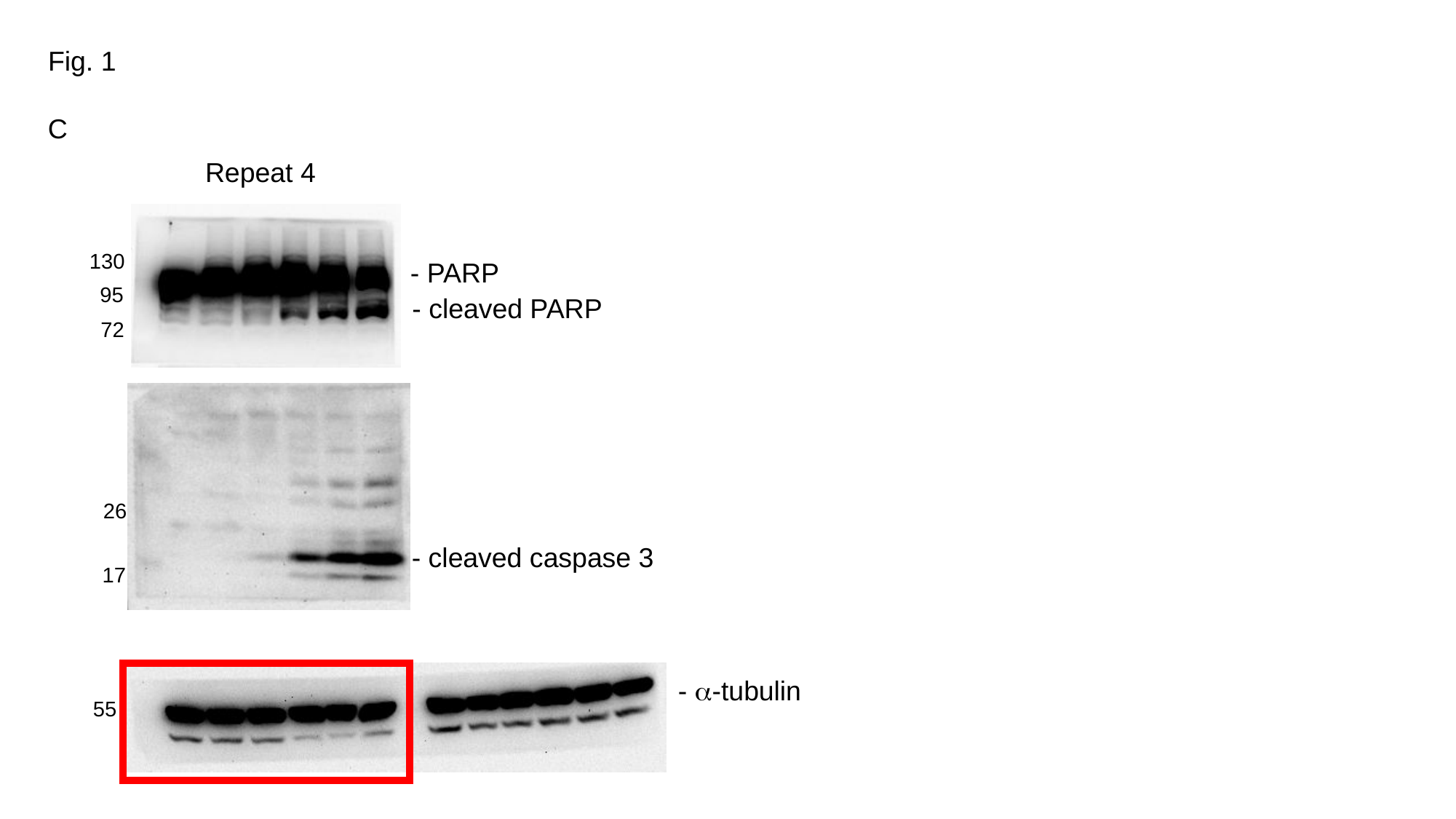

Fig. 1
C
Repeat 4
130
- PARP
95
- cleaved PARP
72
26
- cleaved caspase 3
17
- a-tubulin
55

## Slide 3
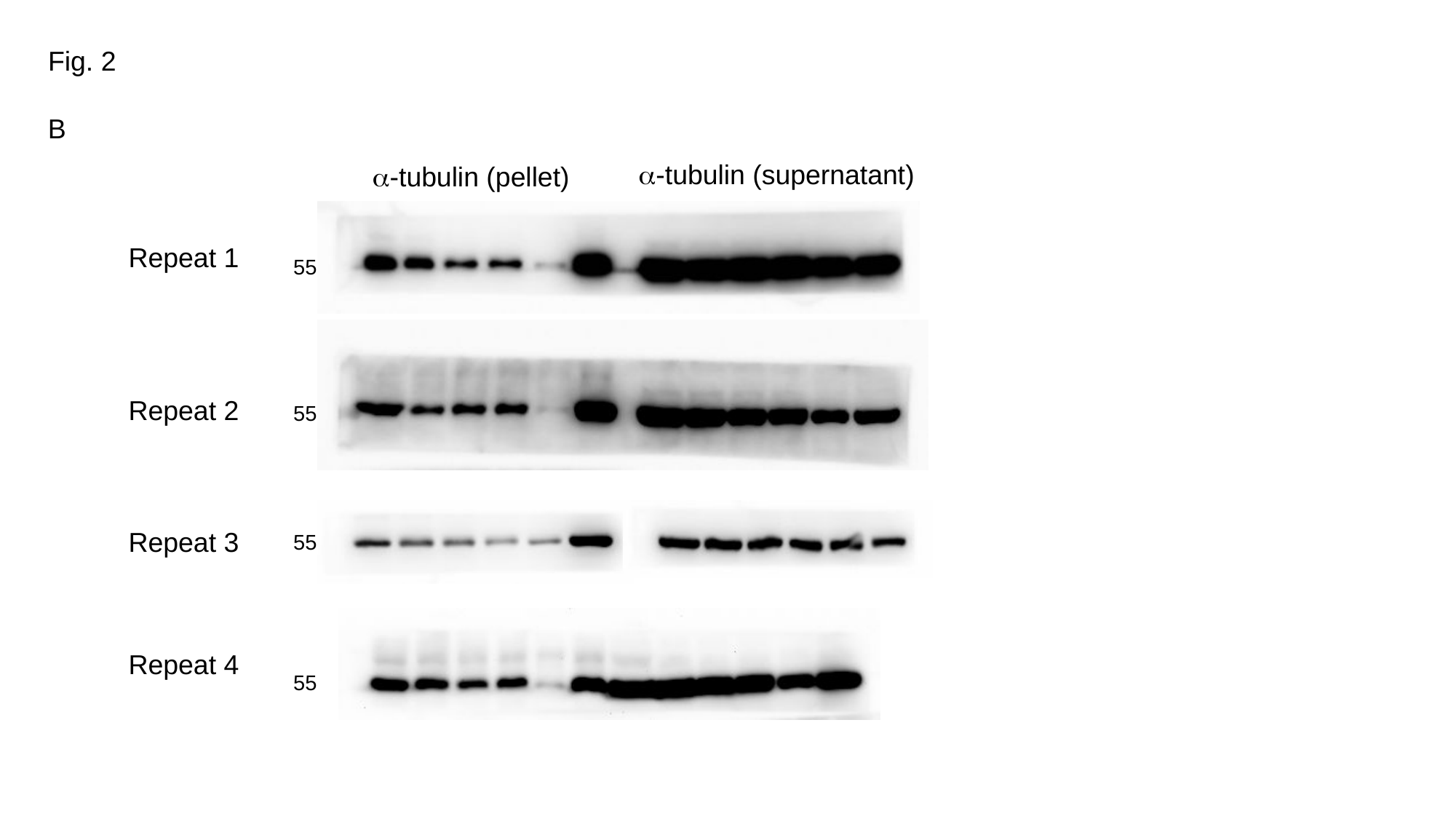

Fig. 2
B
 a-tubulin (supernatant)
 a-tubulin (pellet)
Repeat 1
55
Repeat 2
55
Repeat 3
55
Repeat 4
55

## Slide 4
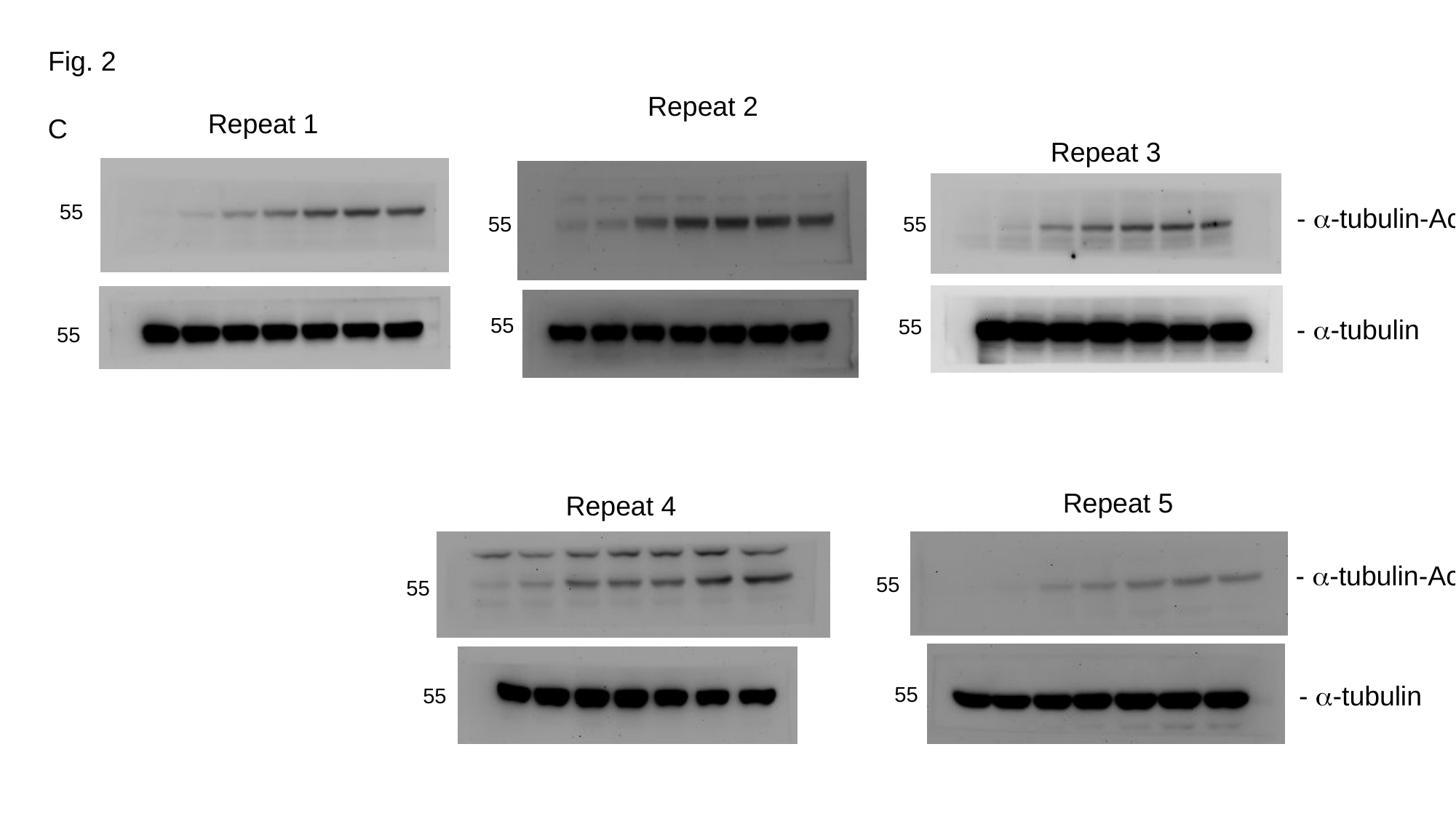

Fig. 2
Repeat 2
Repeat 1
C
Repeat 3
55
- a-tubulin-Ac
55
55
55
- a-tubulin
55
55
Repeat 5
Repeat 4
- a-tubulin-Ac
55
55
- a-tubulin
55
55

## Slide 5
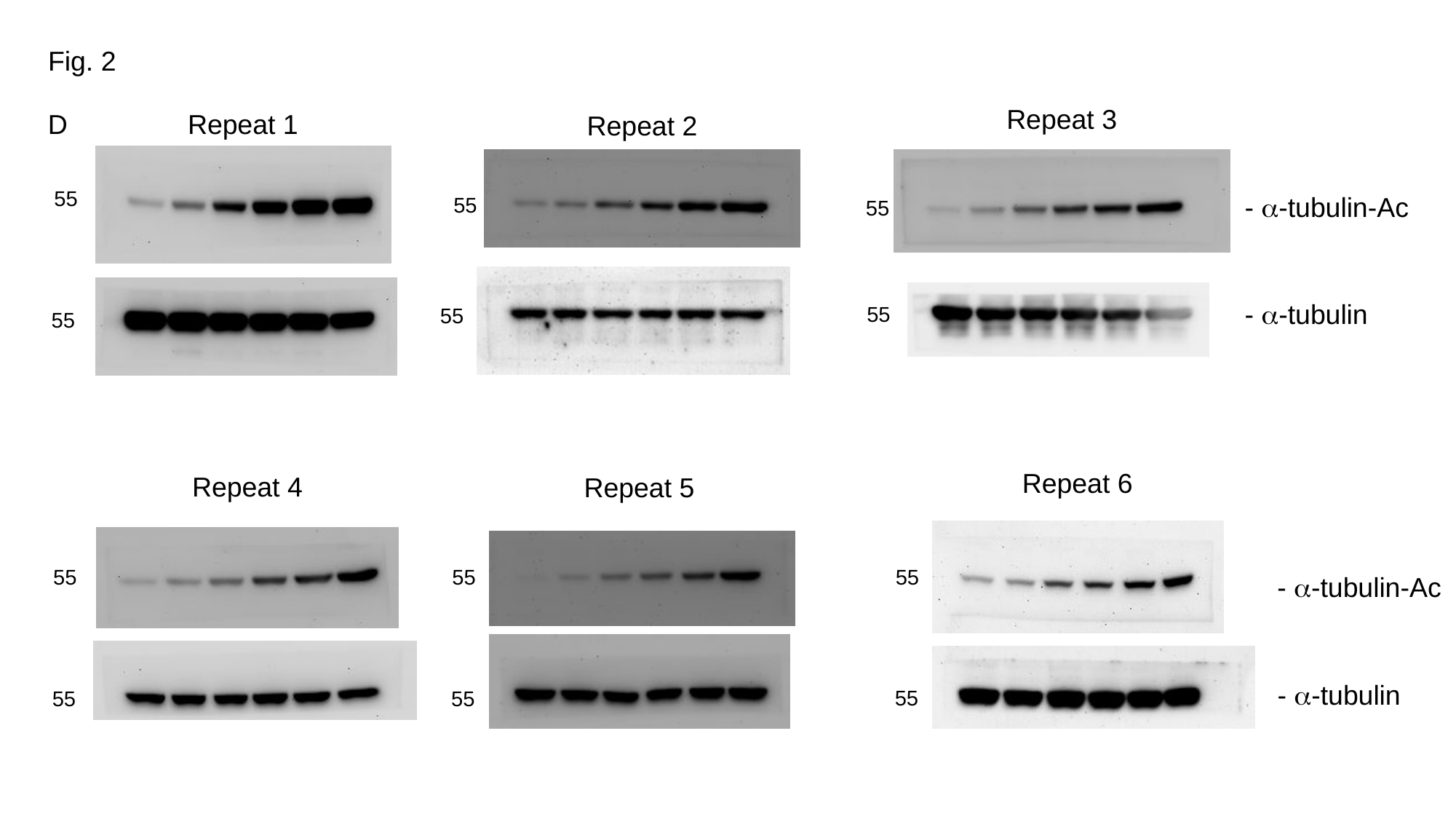

Fig. 2
Repeat 3
D
Repeat 1
Repeat 2
55
- a-tubulin-Ac
55
55
- a-tubulin
55
55
55
Repeat 6
Repeat 4
Repeat 5
55
55
55
- a-tubulin-Ac
- a-tubulin
55
55
55

## Slide 6
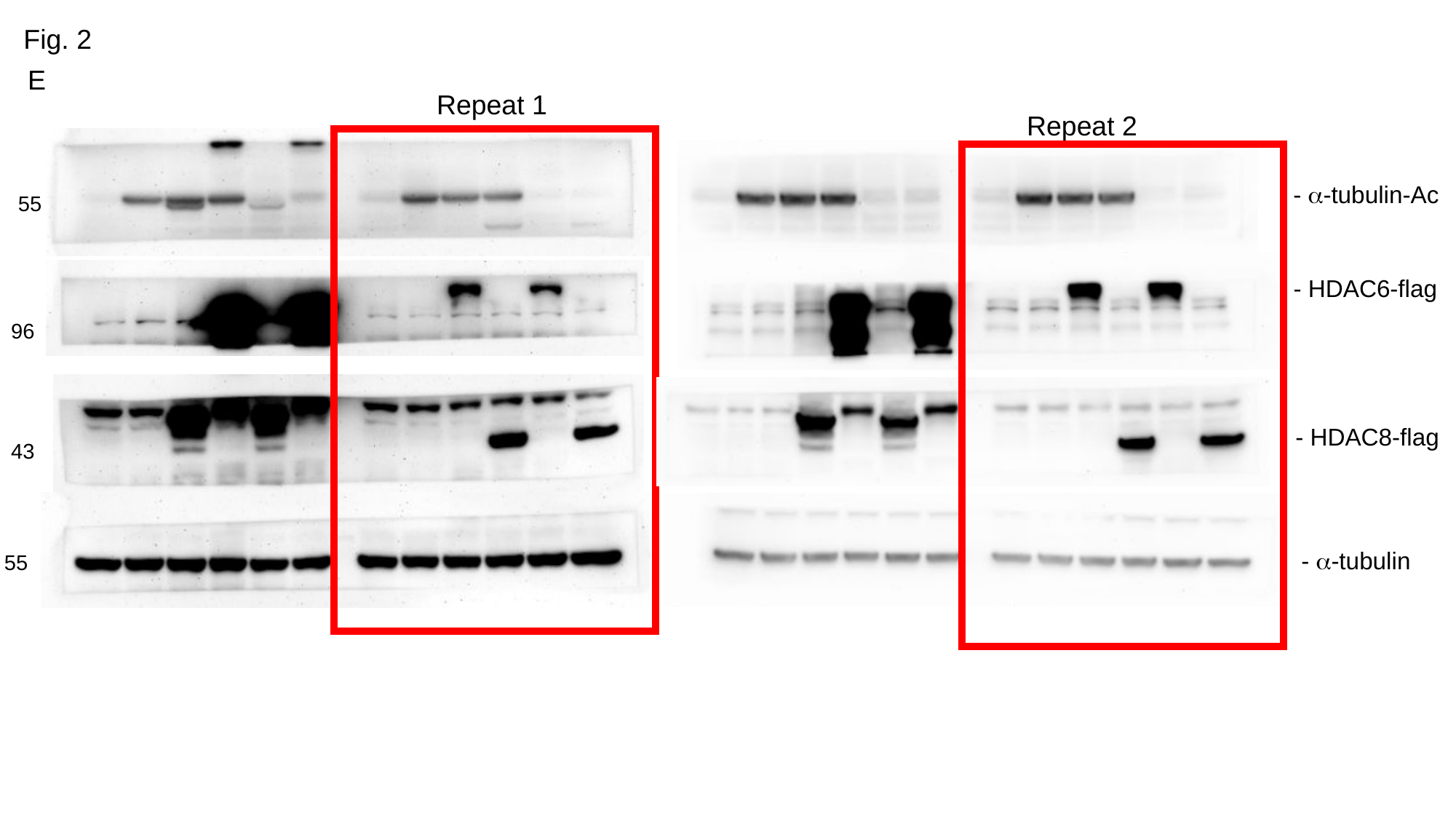

Fig. 2
E
Repeat 1
Repeat 2
- a-tubulin-Ac
55
- HDAC6-flag
96
- HDAC8-flag
43
- a-tubulin
55

## Slide 7
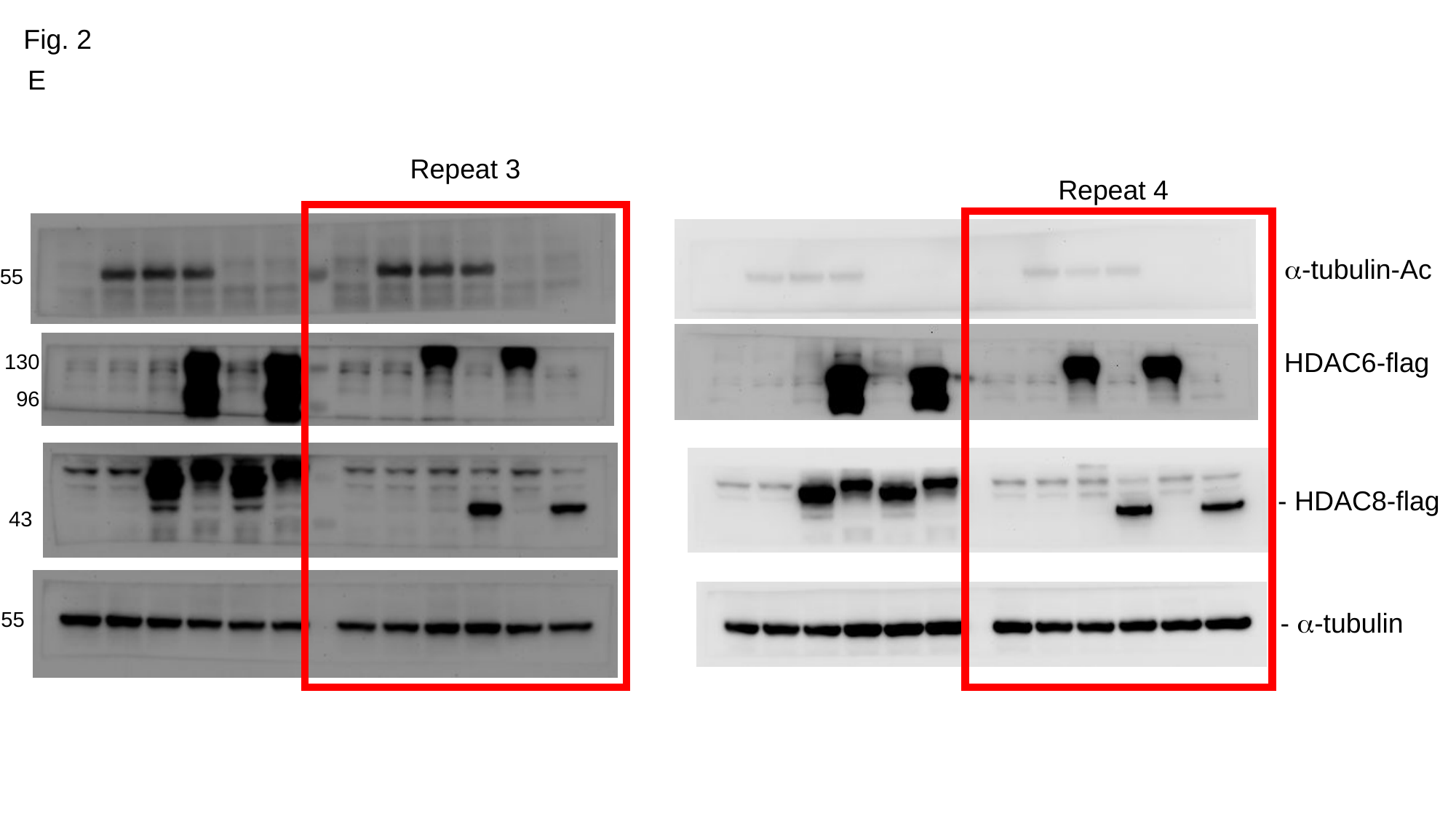

Fig. 2
E
Repeat 3
Repeat 4
- a-tubulin-Ac
55
- HDAC6-flag
130
96
- HDAC8-flag
43
55
- a-tubulin

## Slide 8
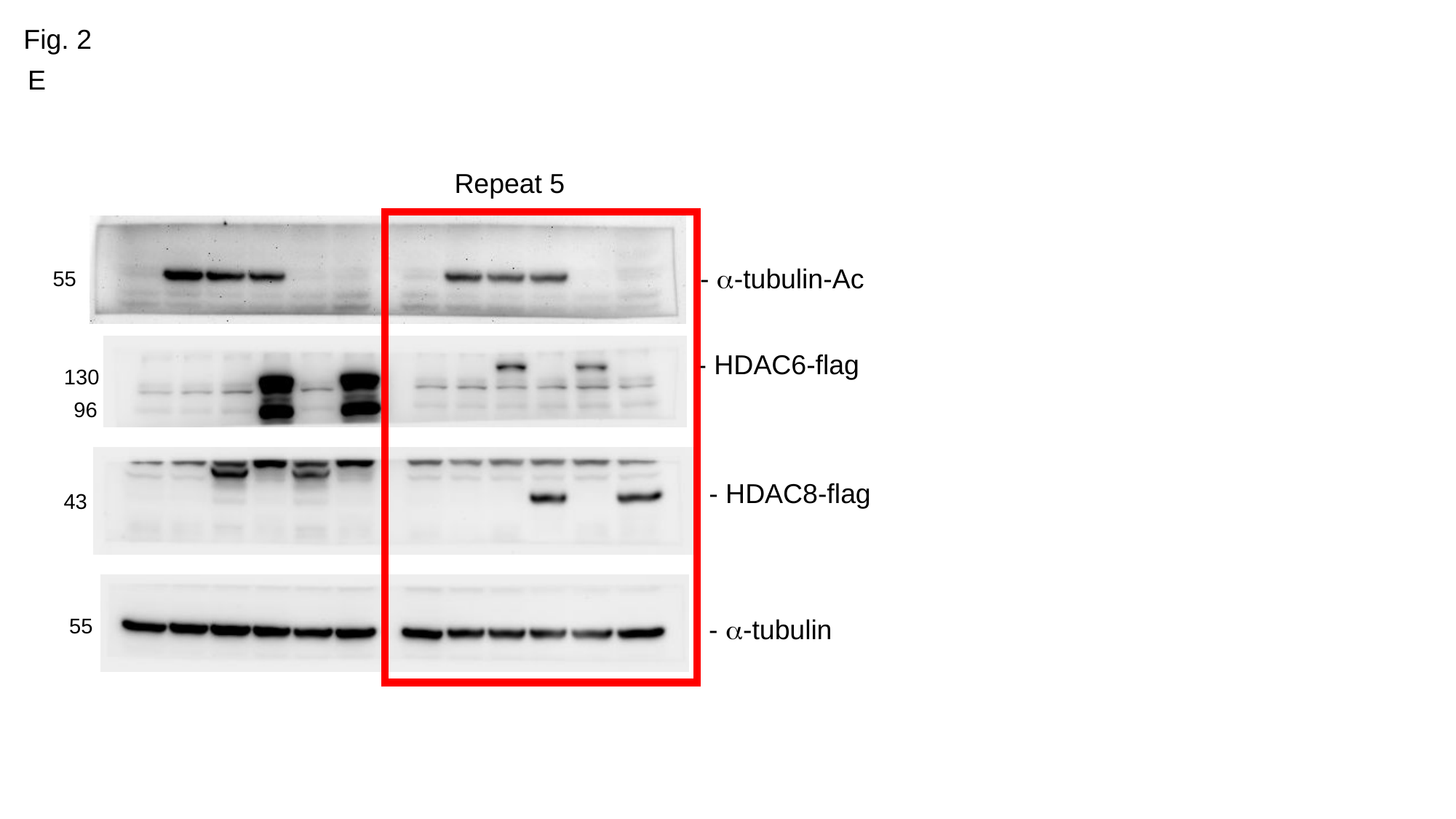

Fig. 2
E
Repeat 5
- a-tubulin-Ac
55
- HDAC6-flag
130
96
- HDAC8-flag
43
55
- a-tubulin

## Slide 9
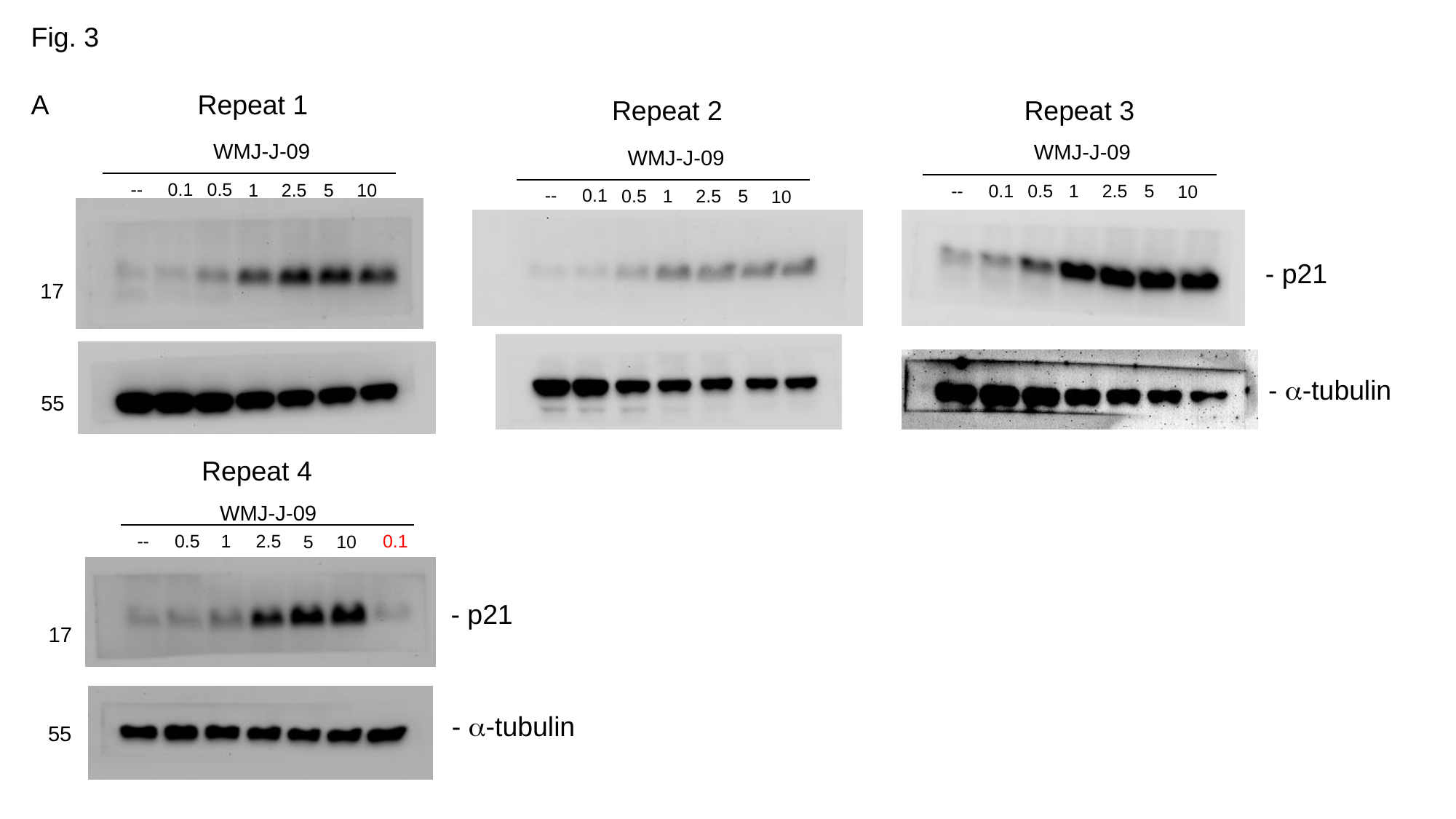

Fig. 3
Repeat 1
A
Repeat 2
Repeat 3
WMJ-J-09
WMJ-J-09
WMJ-J-09
--
0.1
0.5
1
2.5
5
10
--
0.1
0.5
1
2.5
5
10
--
0.1
0.5
1
2.5
5
10
- p21
17
- a-tubulin
55
Repeat 4
WMJ-J-09
--
0.1
0.5
1
2.5
5
10
- p21
17
- a-tubulin
55

## Slide 10
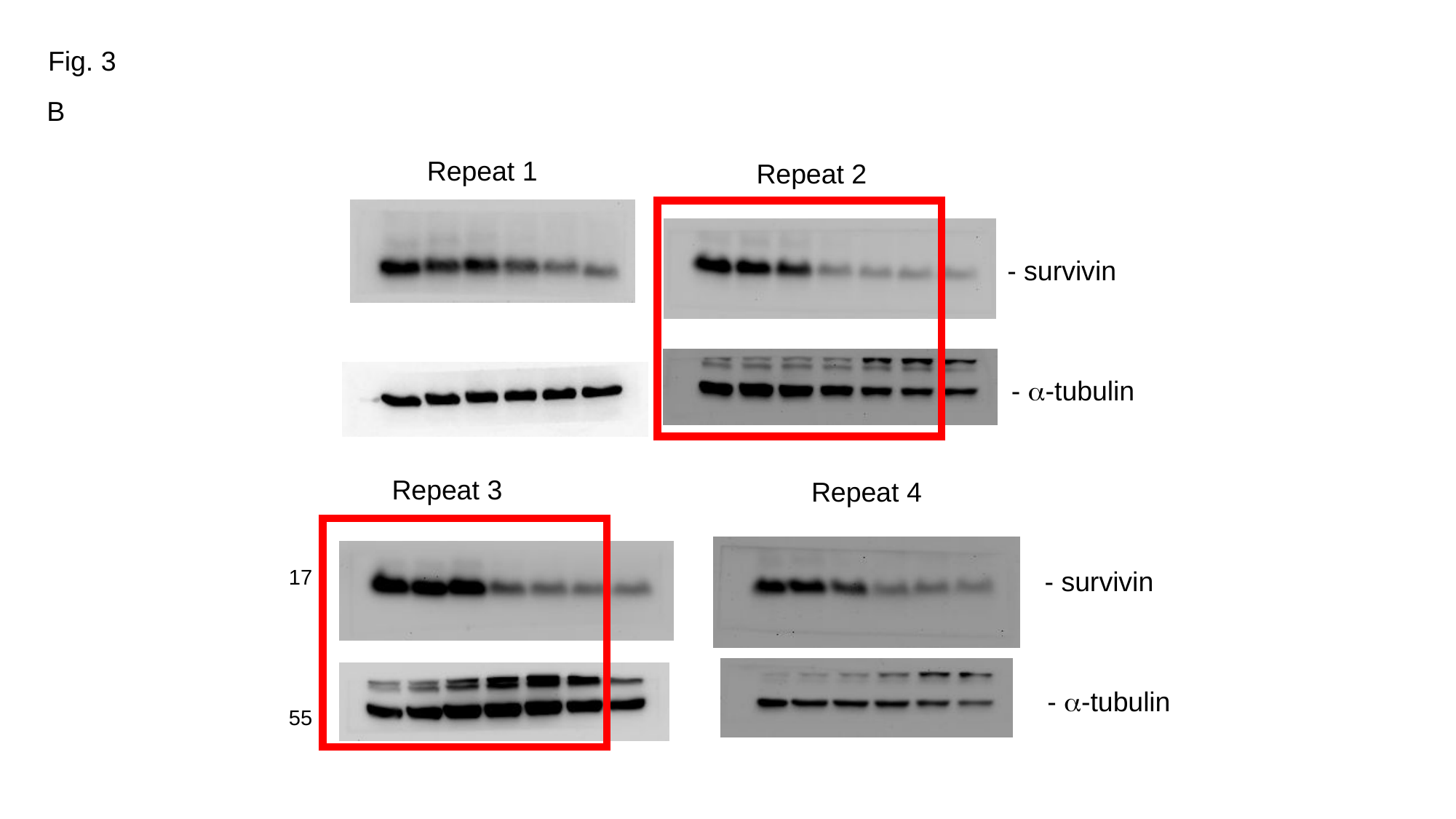

Fig. 3
B
Repeat 1
Repeat 2
- survivin
- a-tubulin
Repeat 3
Repeat 4
17
- survivin
- a-tubulin
55

## Slide 11
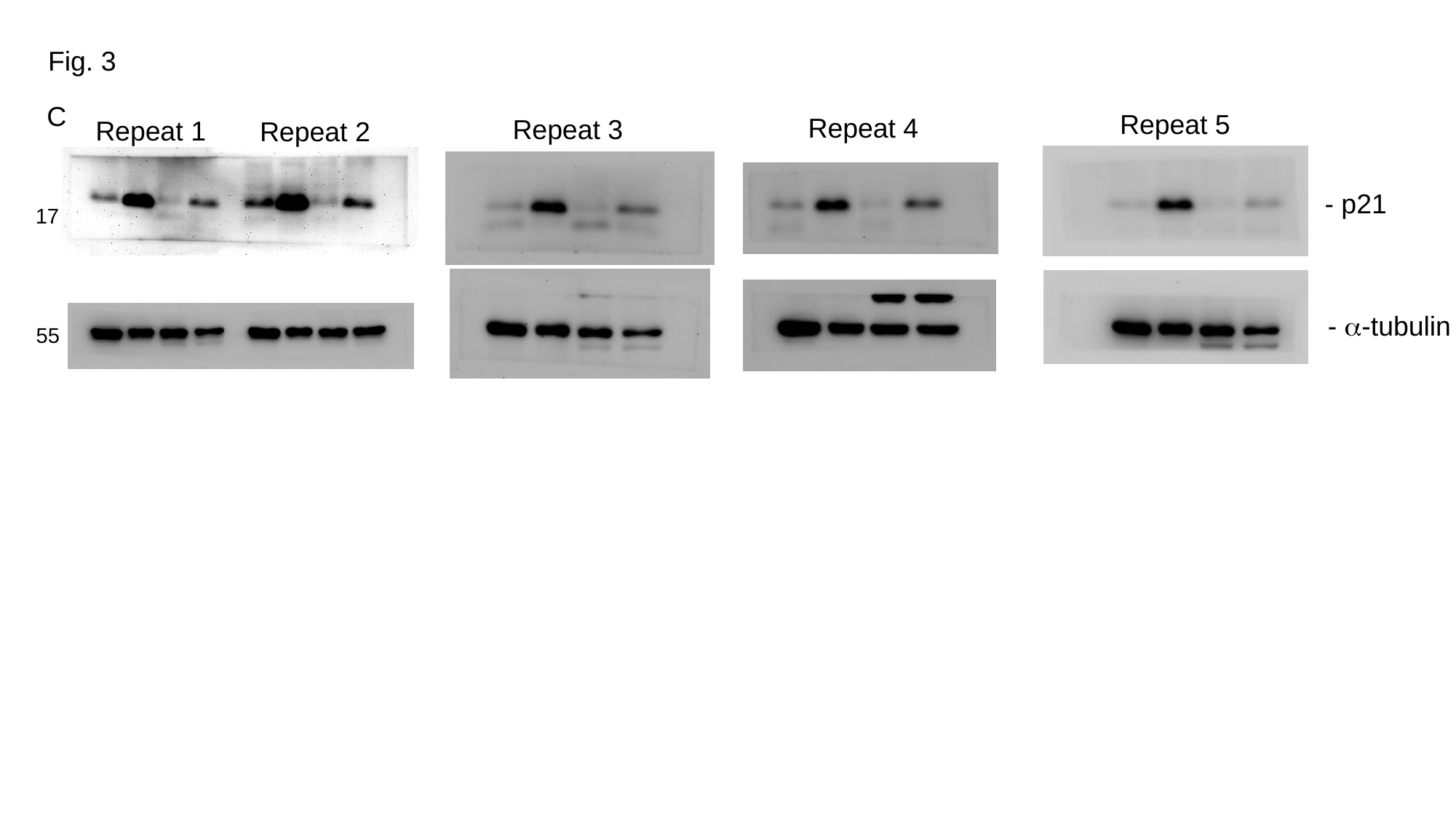

Fig. 3
C
Repeat 5
Repeat 4
Repeat 3
Repeat 1
Repeat 2
- p21
17
- a-tubulin
55

## Slide 12
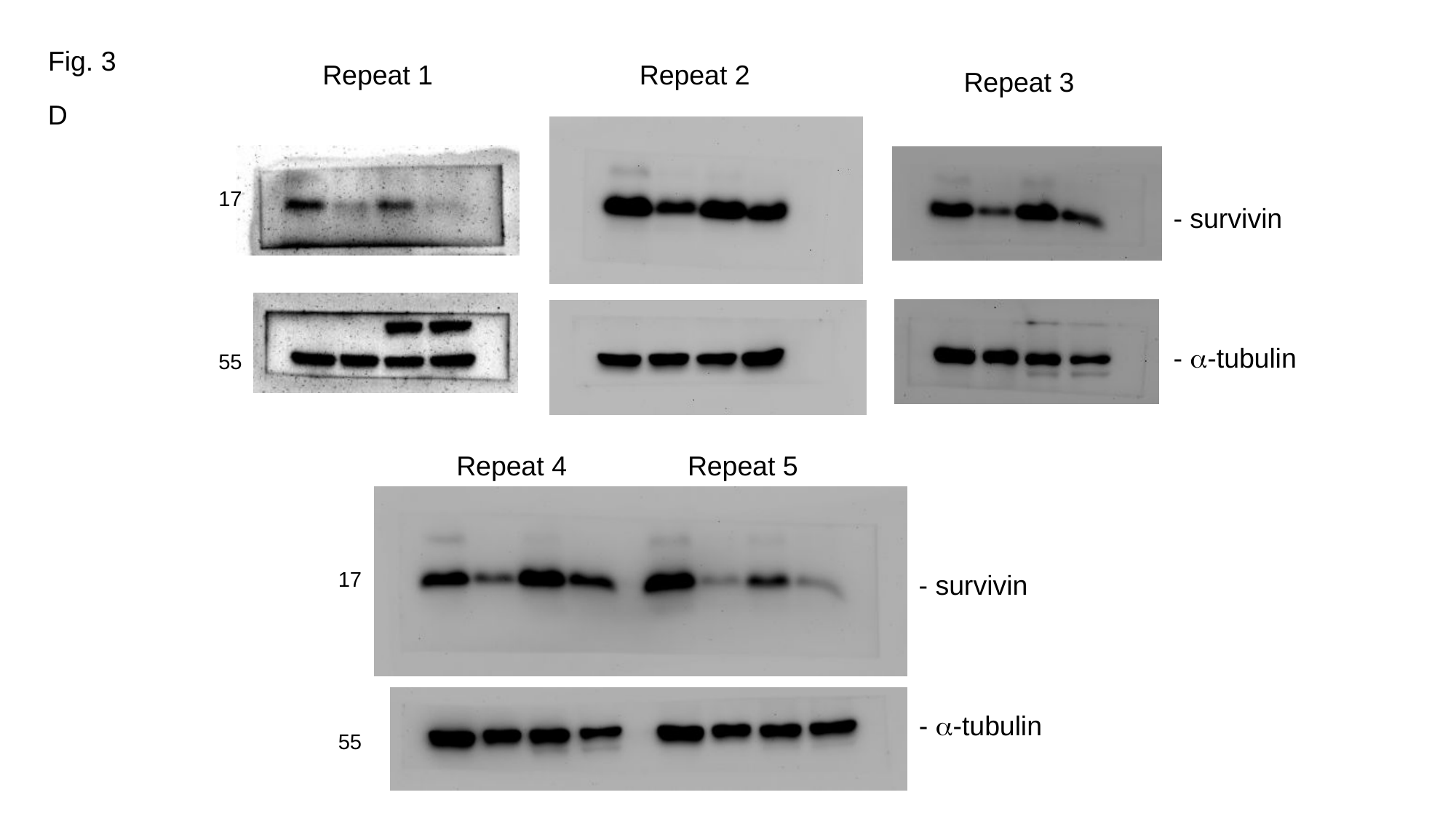

Fig. 3
Repeat 1
Repeat 2
Repeat 3
D
17
- survivin
- a-tubulin
55
Repeat 4
Repeat 5
17
- survivin
- a-tubulin
55

## Slide 13
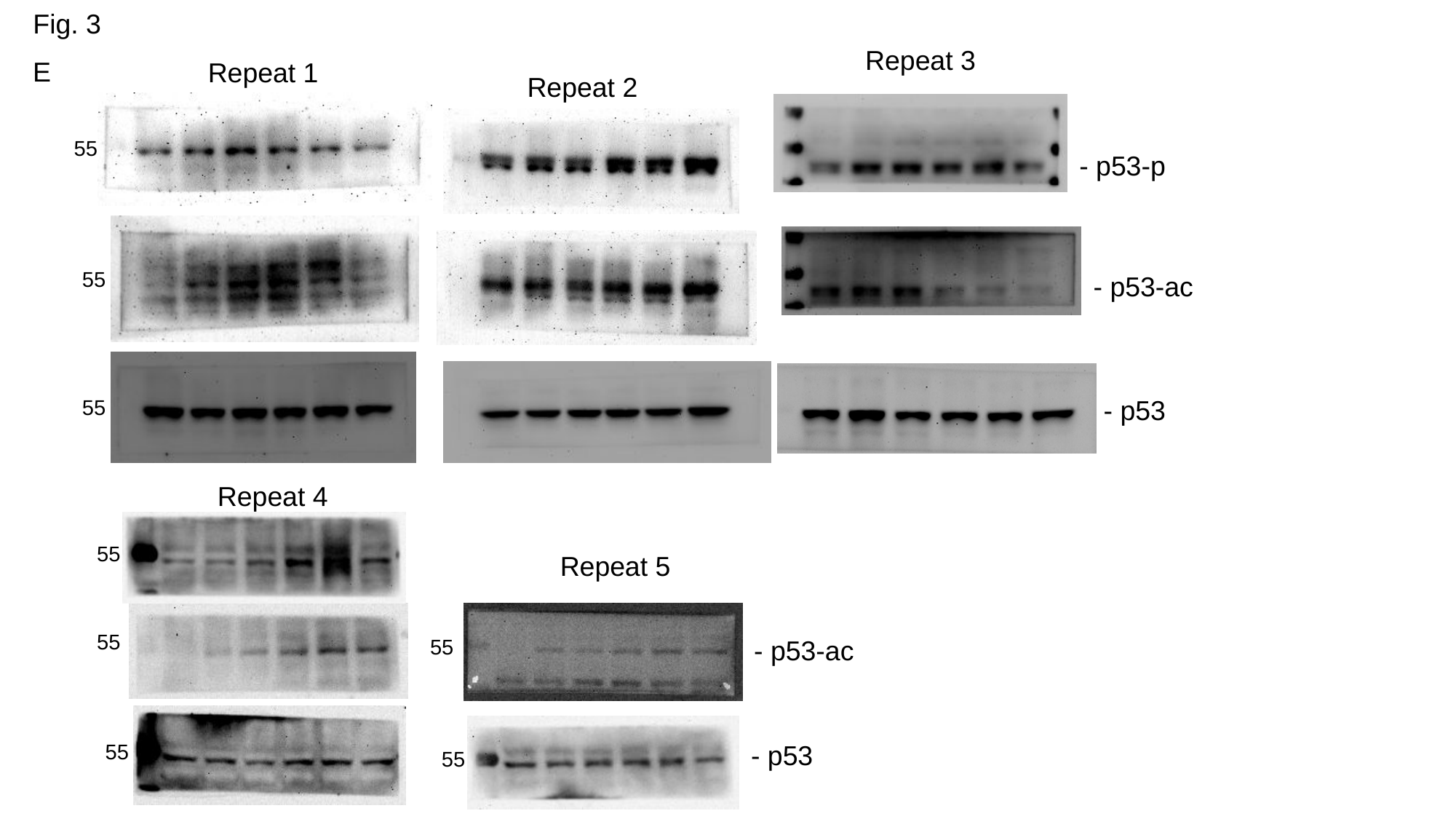

Fig. 3
Repeat 3
E
Repeat 1
Repeat 2
55
- p53-p
55
- p53-ac
- p53
55
Repeat 4
55
Repeat 5
55
55
- p53-ac
55
- p53
55

## Slide 14
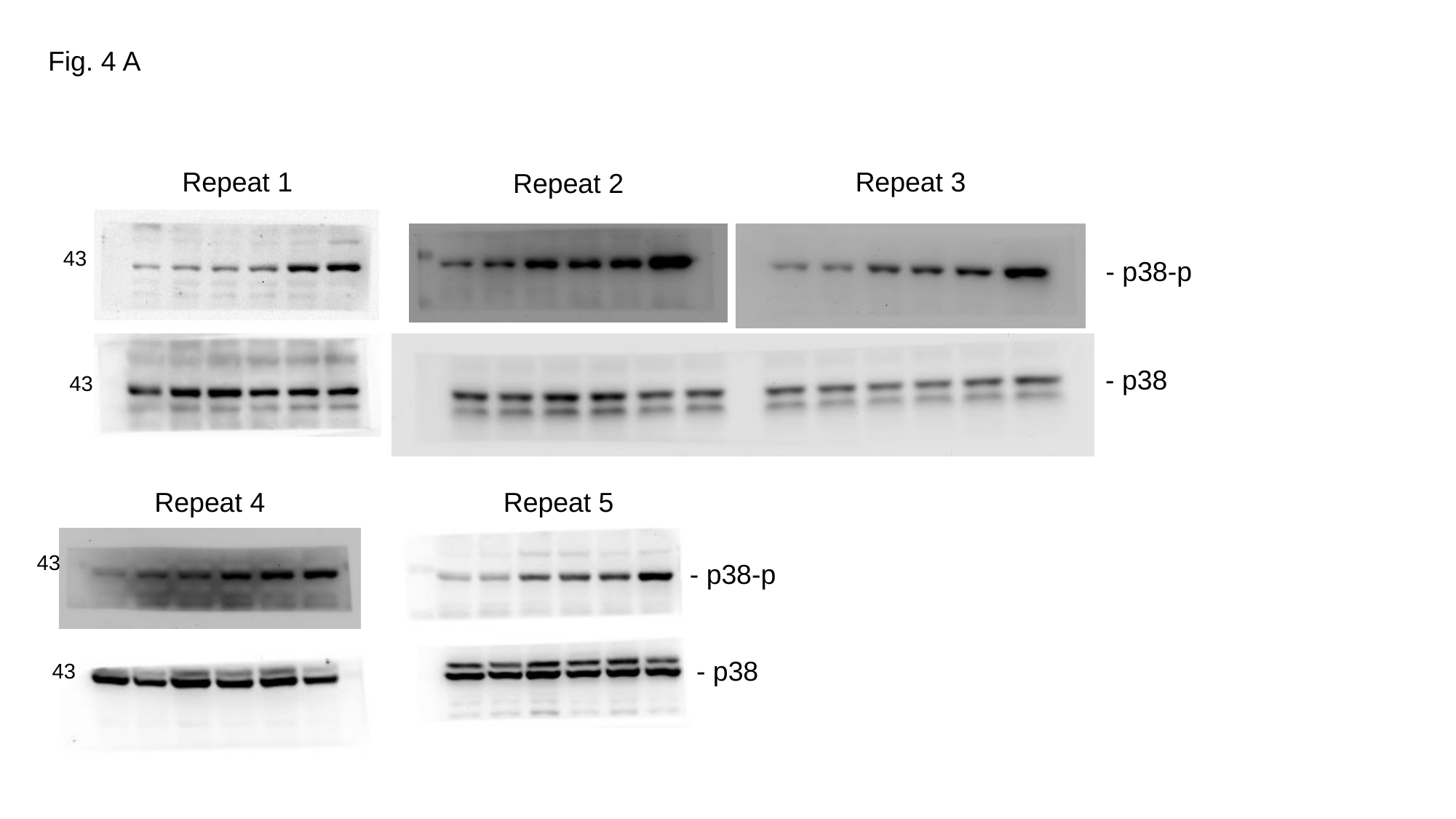

Fig. 4
A
Repeat 1
Repeat 3
Repeat 2
43
- p38-p
- p38
43
Repeat 4
Repeat 5
43
- p38-p
- p38
43

## Slide 15
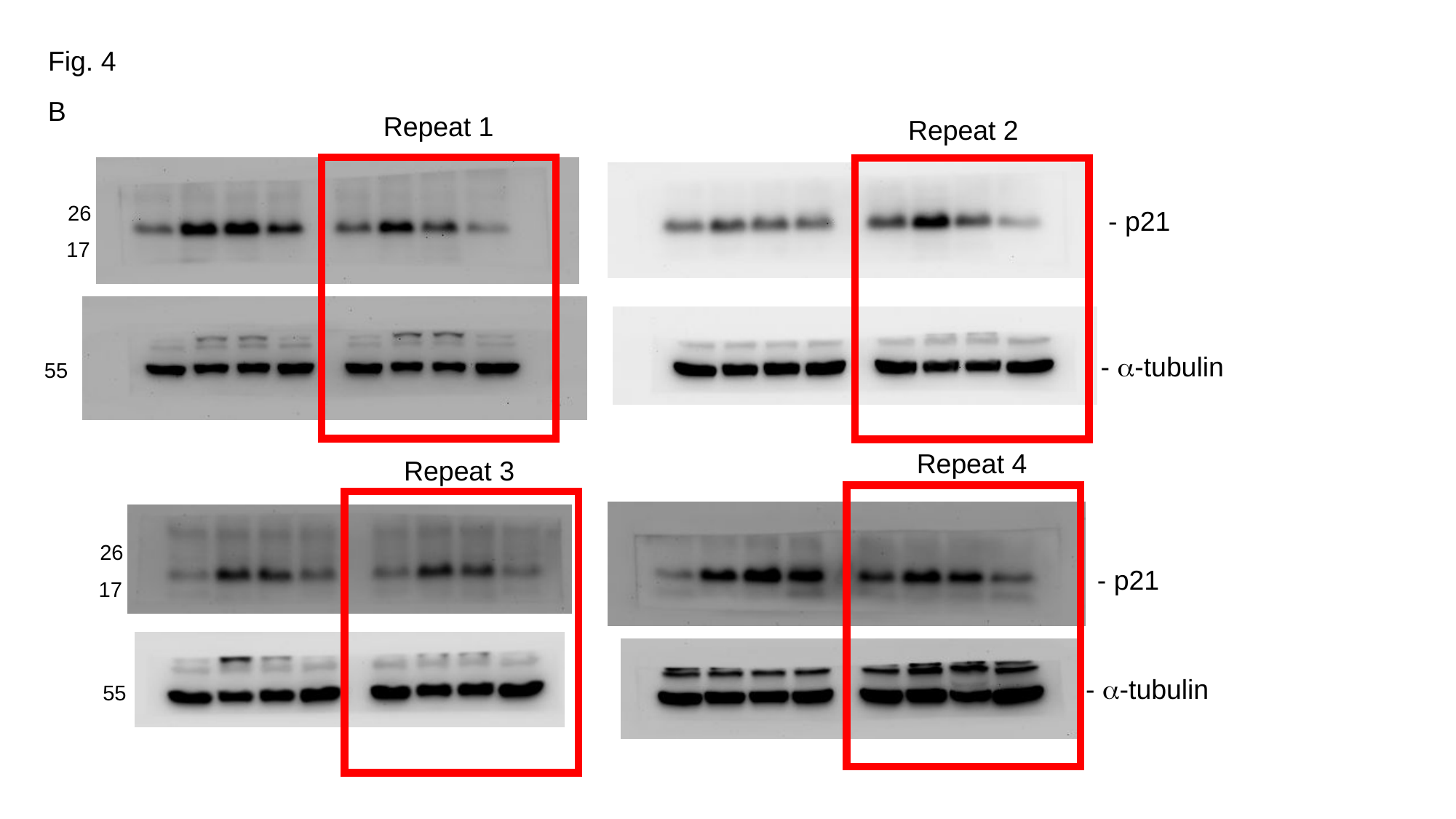

Fig. 4
B
Repeat 1
Repeat 2
26
- p21
17
- a-tubulin
55
Repeat 4
Repeat 3
26
- p21
17
- a-tubulin
55

## Slide 16
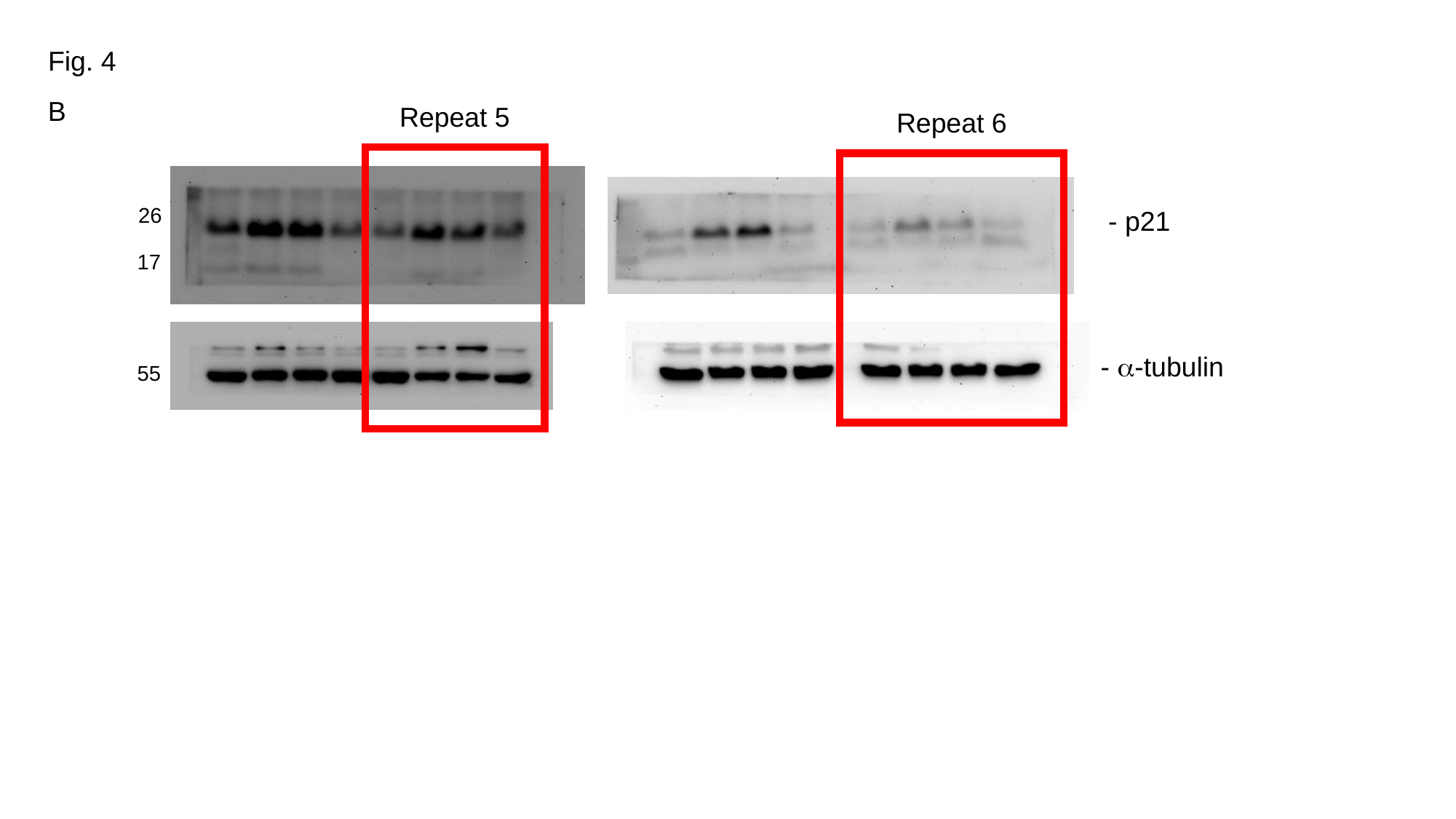

Fig. 4
B
Repeat 5
Repeat 6
26
- p21
17
- a-tubulin
55

## Slide 17
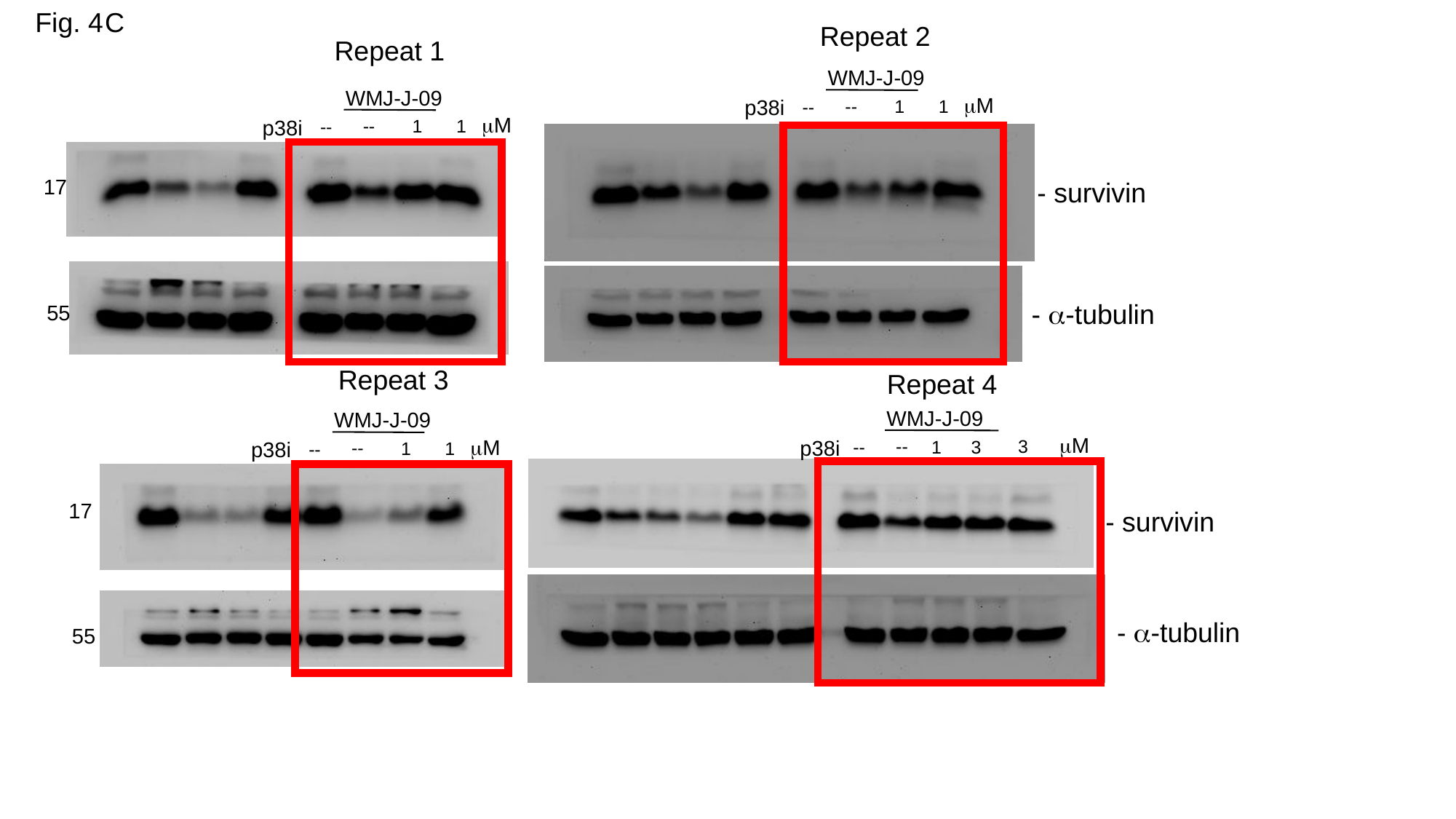

Fig. 4
C
Repeat 2
Repeat 1
WMJ-J-09
WMJ-J-09
mM
p38i
--
1
1
--
mM
p38i
--
1
1
--
17
- survivin
- a-tubulin
55
Repeat 3
Repeat 4
WMJ-J-09
WMJ-J-09
mM
mM
p38i
--
3
1
3
--
p38i
--
1
1
--
17
- survivin
- a-tubulin
55

## Slide 18
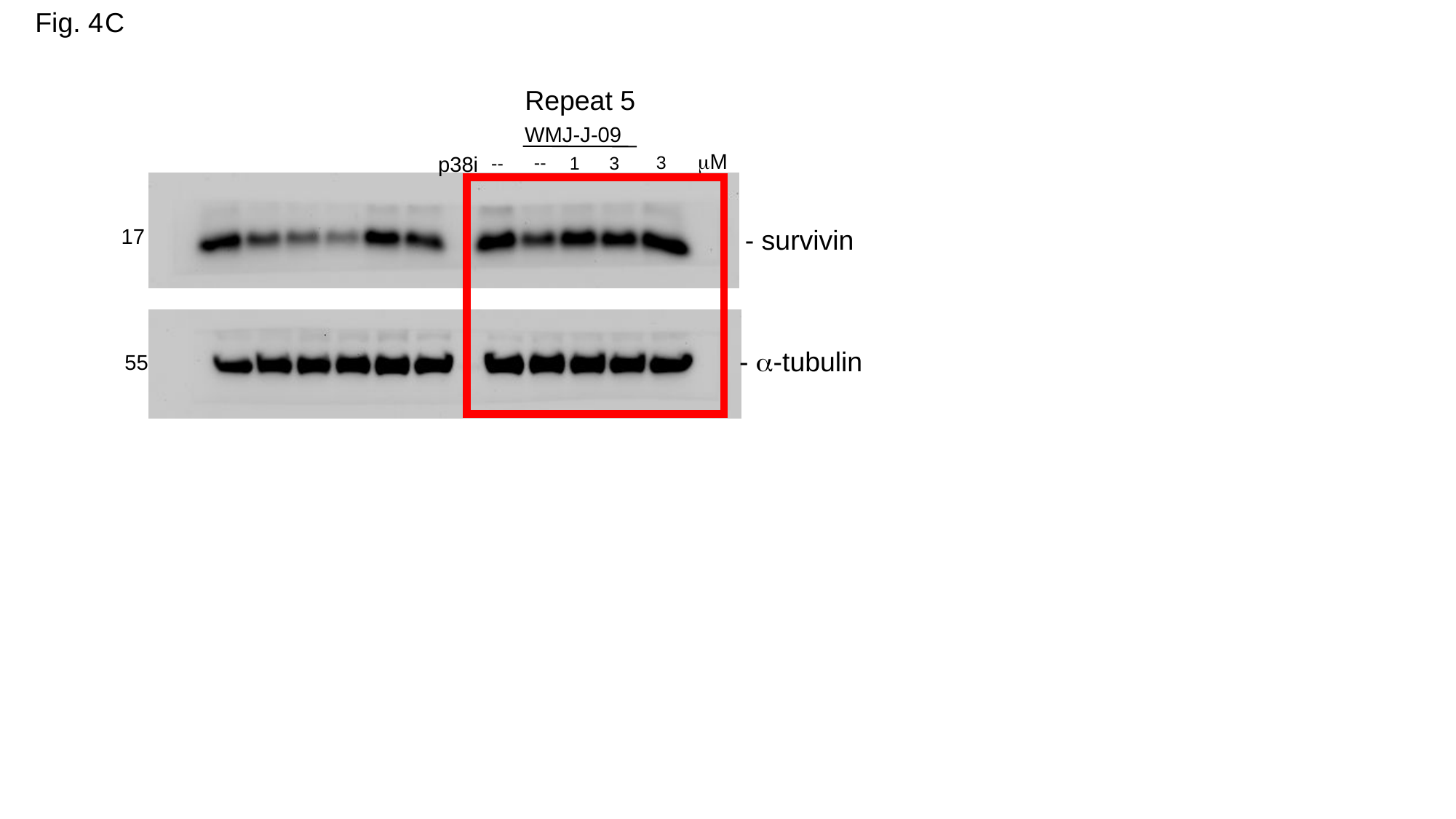

Fig. 4
C
Repeat 5
WMJ-J-09
mM
p38i
--
3
1
3
--
17
- survivin
- a-tubulin
55

## Slide 19
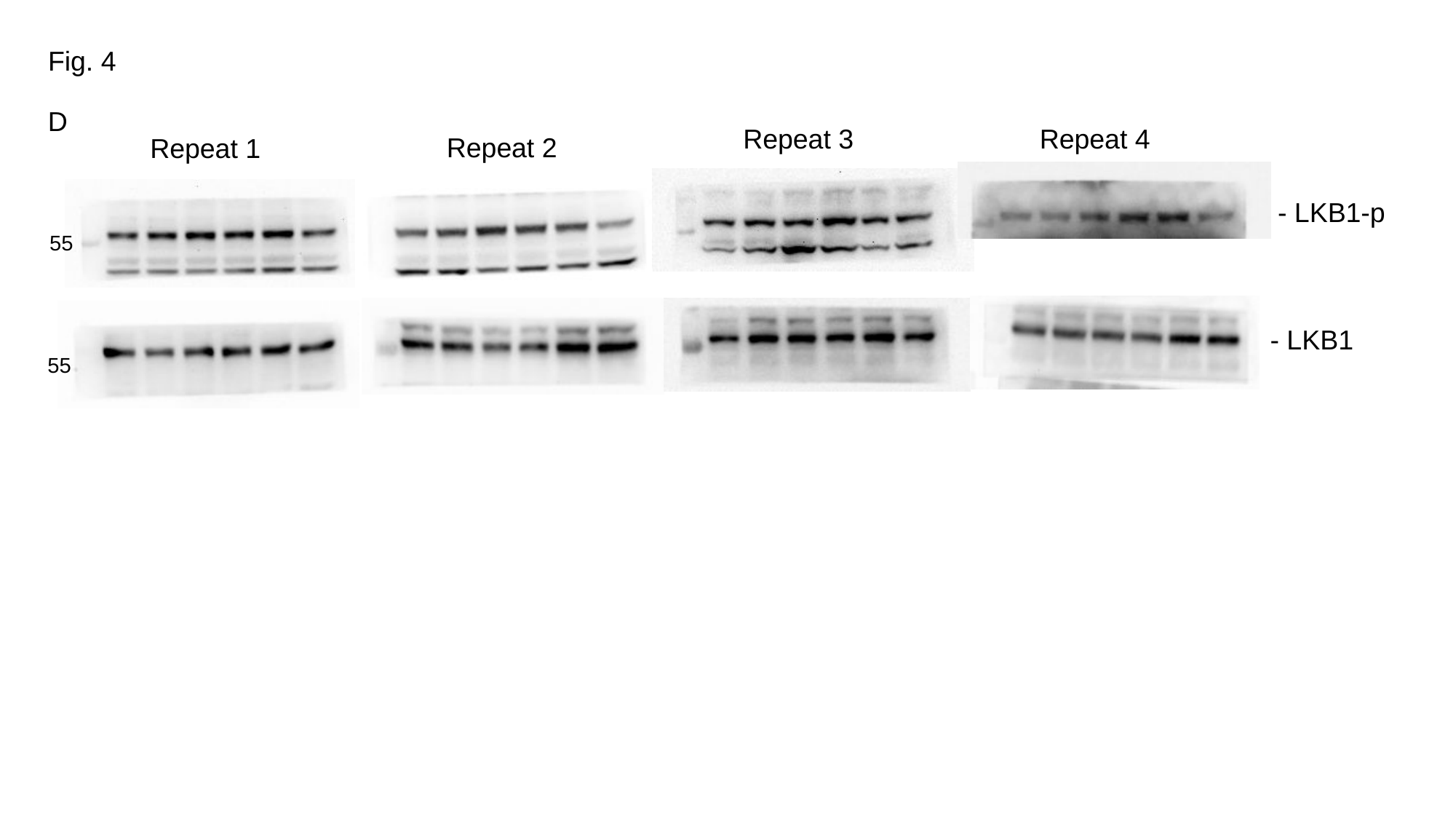

Fig. 4
D
Repeat 3
Repeat 4
Repeat 2
Repeat 1
- LKB1-p
55
- LKB1
55

## Slide 20
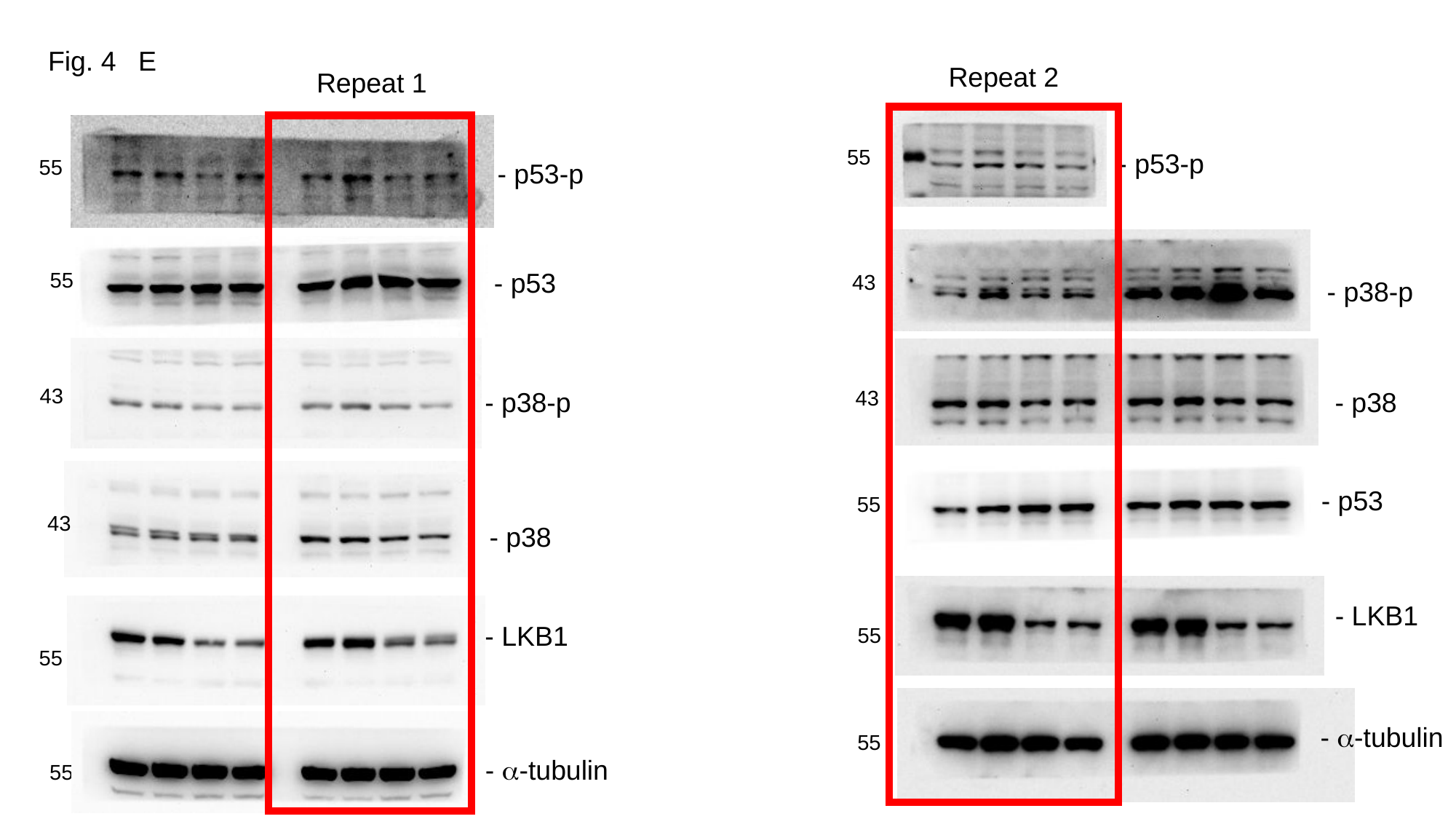

Fig. 4
E
Repeat 2
Repeat 1
55
- p53-p
55
- p53-p
- p53
55
43
- p38-p
43
43
- p38-p
- p38
- p53
55
43
- p38
- LKB1
- LKB1
55
55
- a-tubulin
55
- a-tubulin
55

## Slide 21
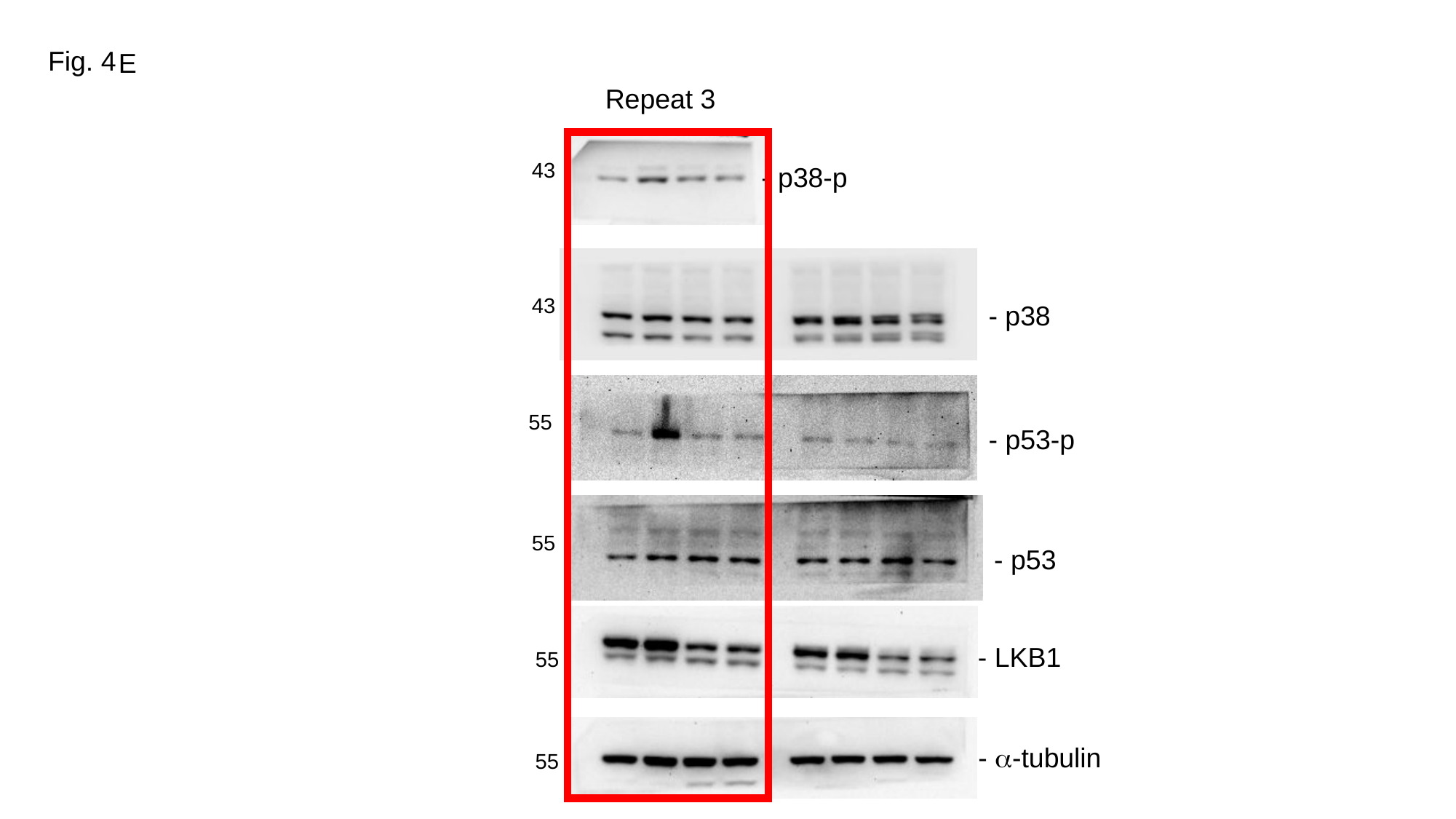

Fig. 4
E
Repeat 3
43
- p38-p
43
- p38
55
- p53-p
55
- p53
- LKB1
55
- a-tubulin
55

## Slide 22
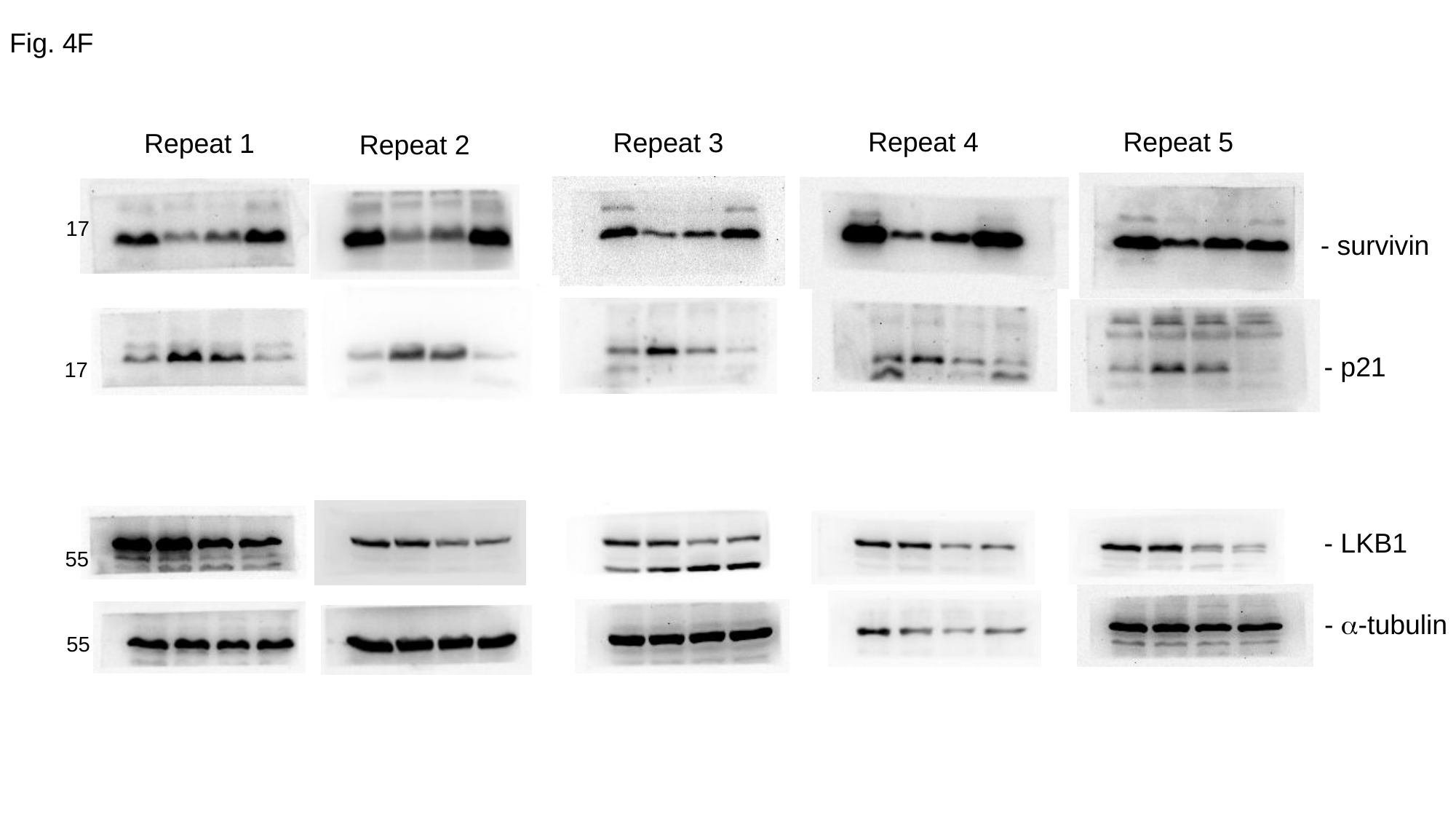

F
Fig. 4
Repeat 4
Repeat 5
Repeat 3
Repeat 1
Repeat 2
17
- survivin
- p21
17
- LKB1
55
- a-tubulin
55

## Slide 23
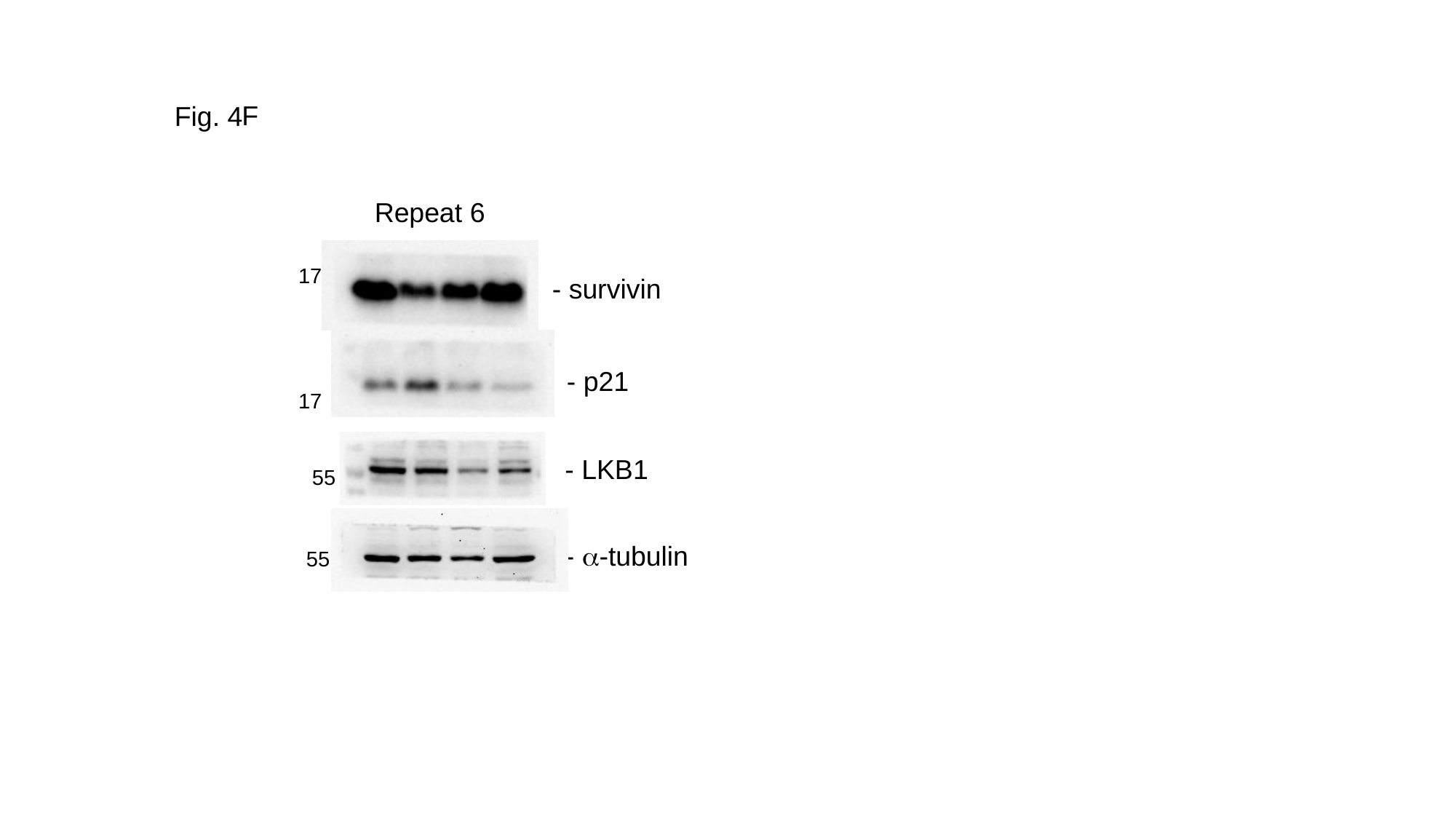

F
Fig. 4
Repeat 6
17
- survivin
- p21
17
- LKB1
55
- a-tubulin
55

## Slide 24
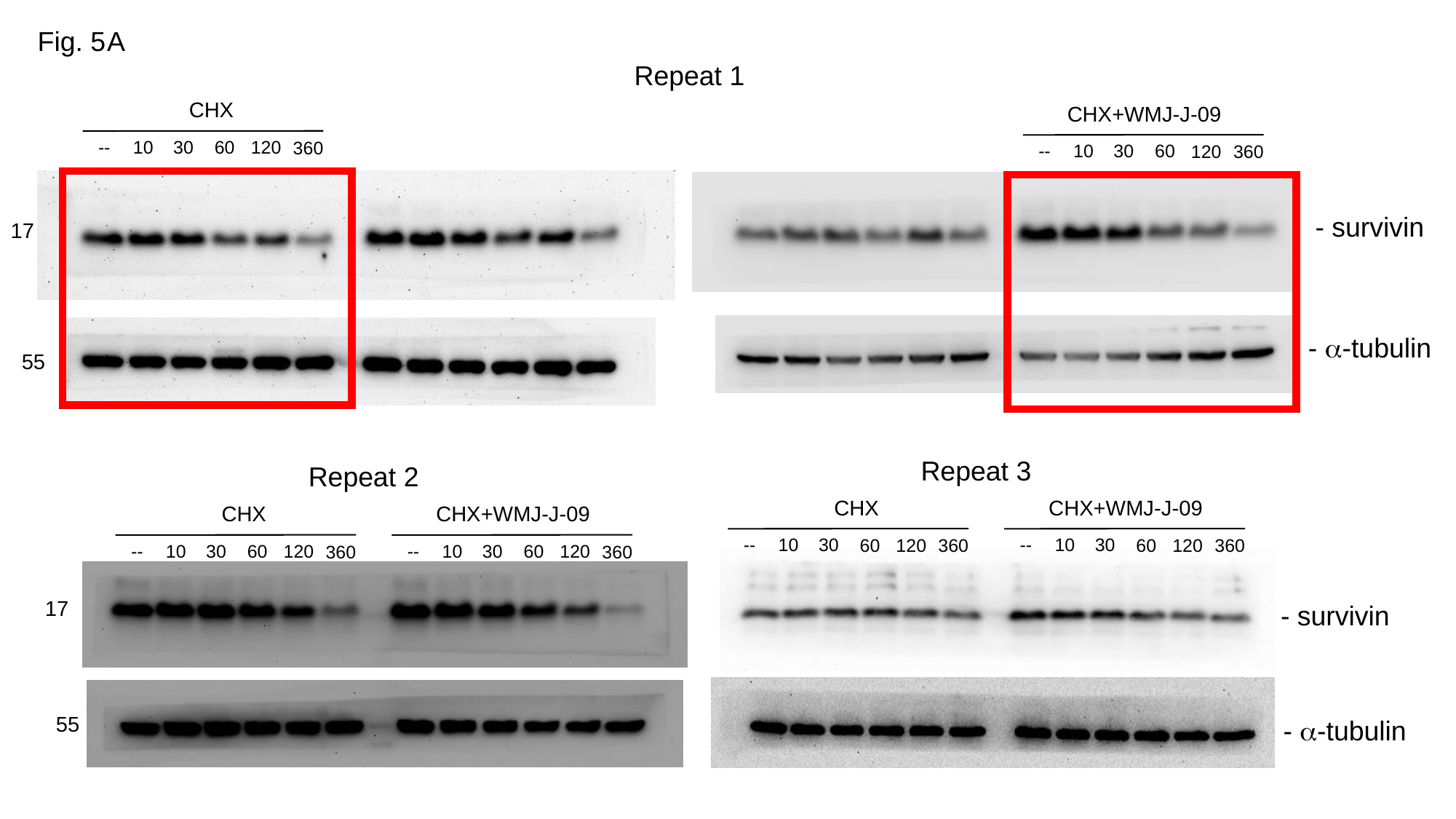

Fig. 5
A
Repeat 1
CHX
CHX+WMJ-J-09
--
10
30
60
120
360
--
10
30
60
120
360
- survivin
17
- a-tubulin
55
Repeat 3
Repeat 2
CHX
CHX+WMJ-J-09
CHX
CHX+WMJ-J-09
--
--
10
10
30
30
60
60
120
120
360
360
--
--
10
10
30
30
60
60
120
120
360
360
17
- survivin
55
- a-tubulin

## Slide 25
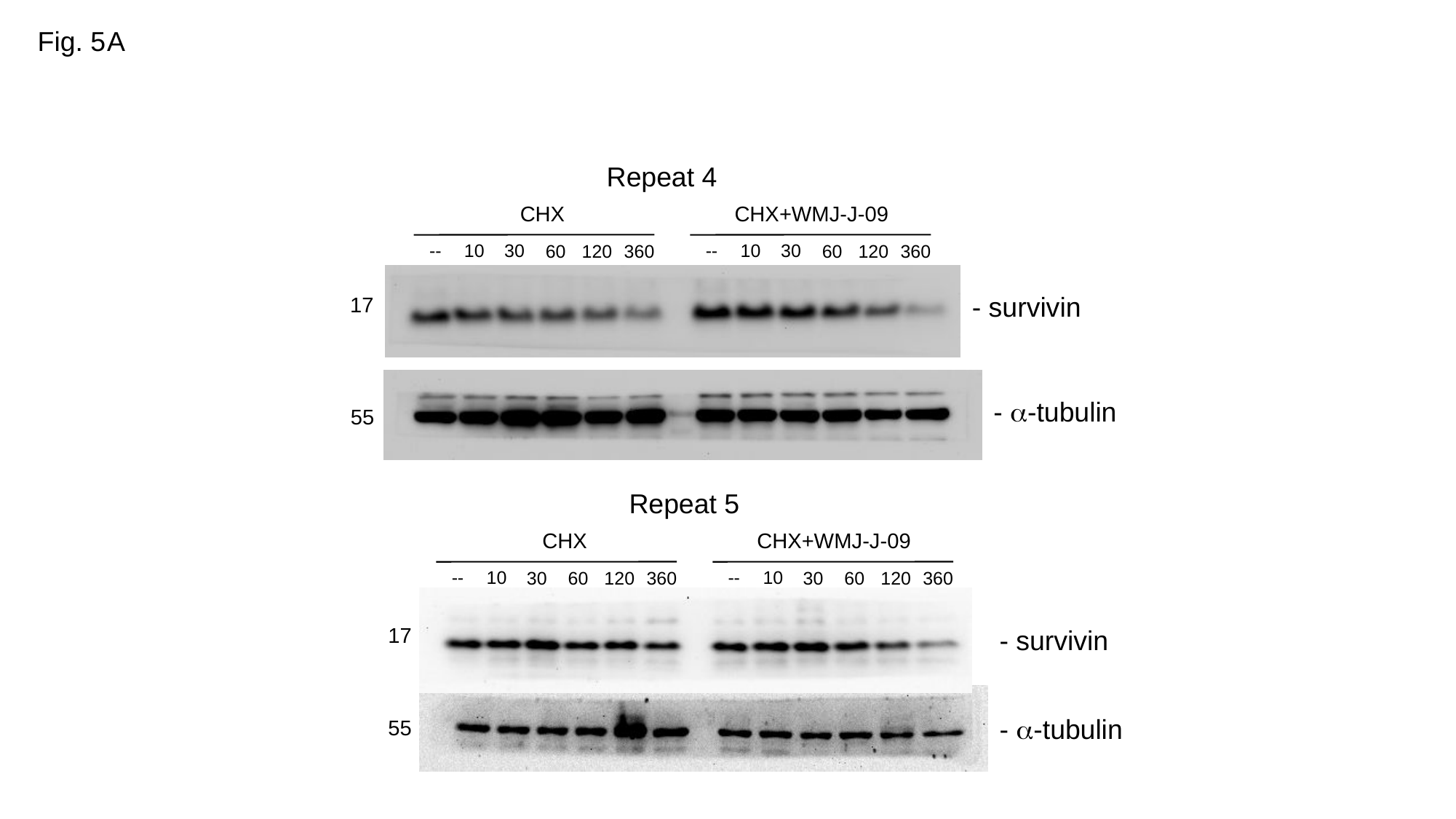

Fig. 5
A
Repeat 4
CHX
CHX+WMJ-J-09
--
--
10
10
30
30
60
60
120
120
360
360
- survivin
17
- a-tubulin
55
Repeat 5
CHX
CHX+WMJ-J-09
--
--
10
10
30
30
60
60
120
120
360
360
17
- survivin
- a-tubulin
55

## Slide 26
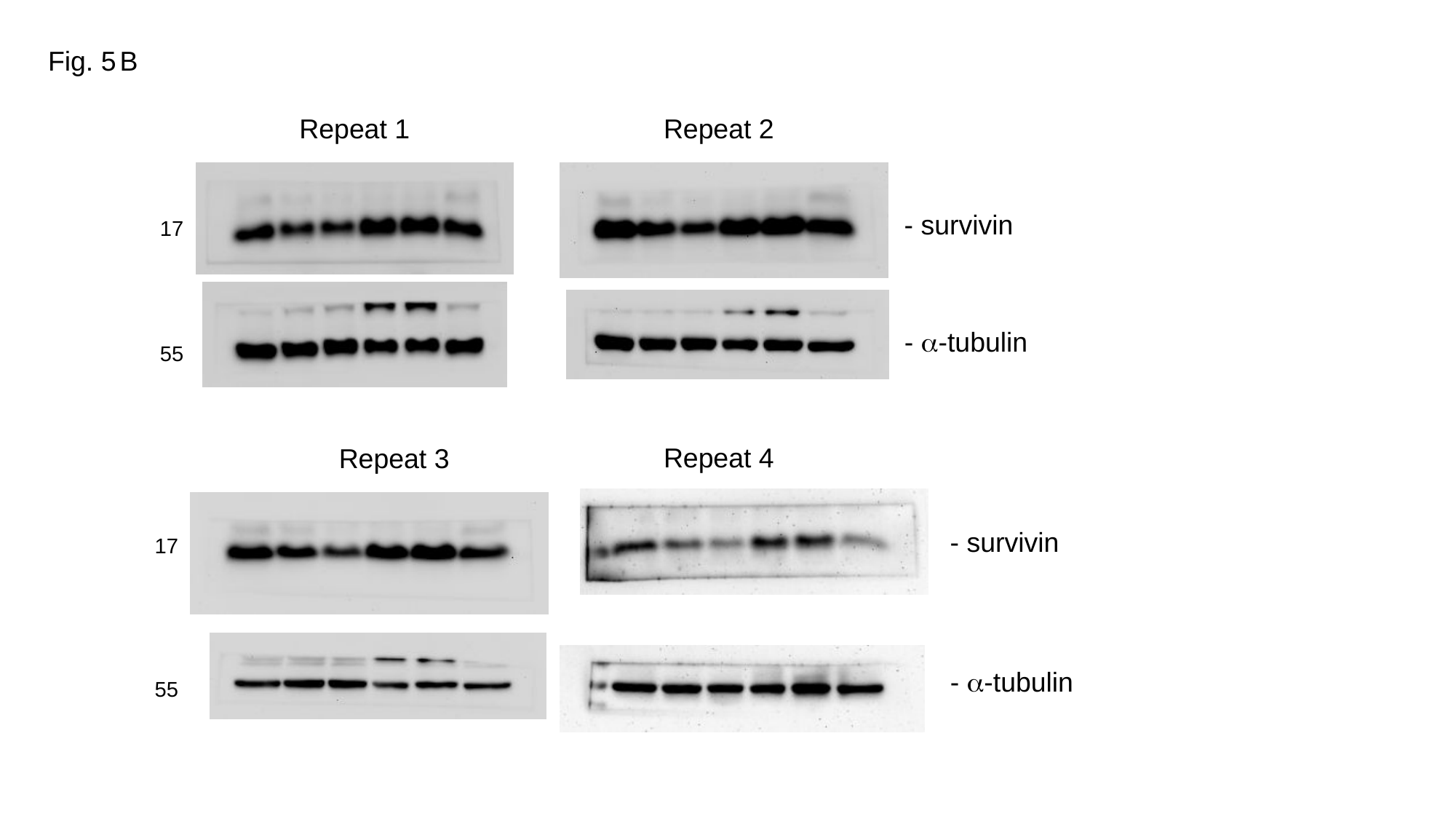

Fig. 5
B
Repeat 1
Repeat 2
- survivin
17
- a-tubulin
55
Repeat 4
Repeat 3
- survivin
17
- a-tubulin
55

## Slide 27
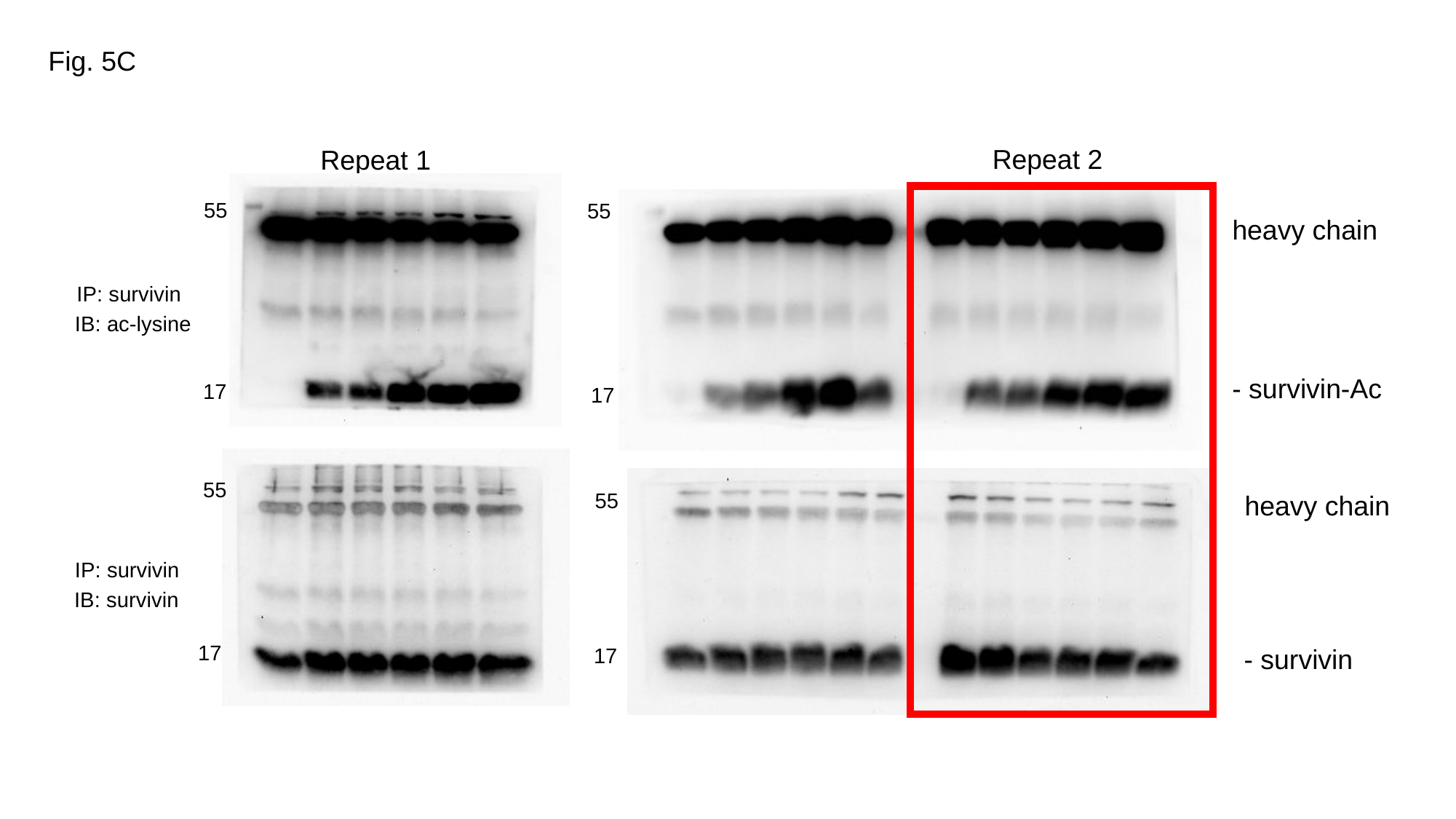

Fig. 5C
Repeat 2
Repeat 1
55
55
heavy chain
IP: survivin
IB: ac-lysine
- survivin-Ac
17
17
55
55
heavy chain
IP: survivin
IB: survivin
17
- survivin
17

## Slide 28
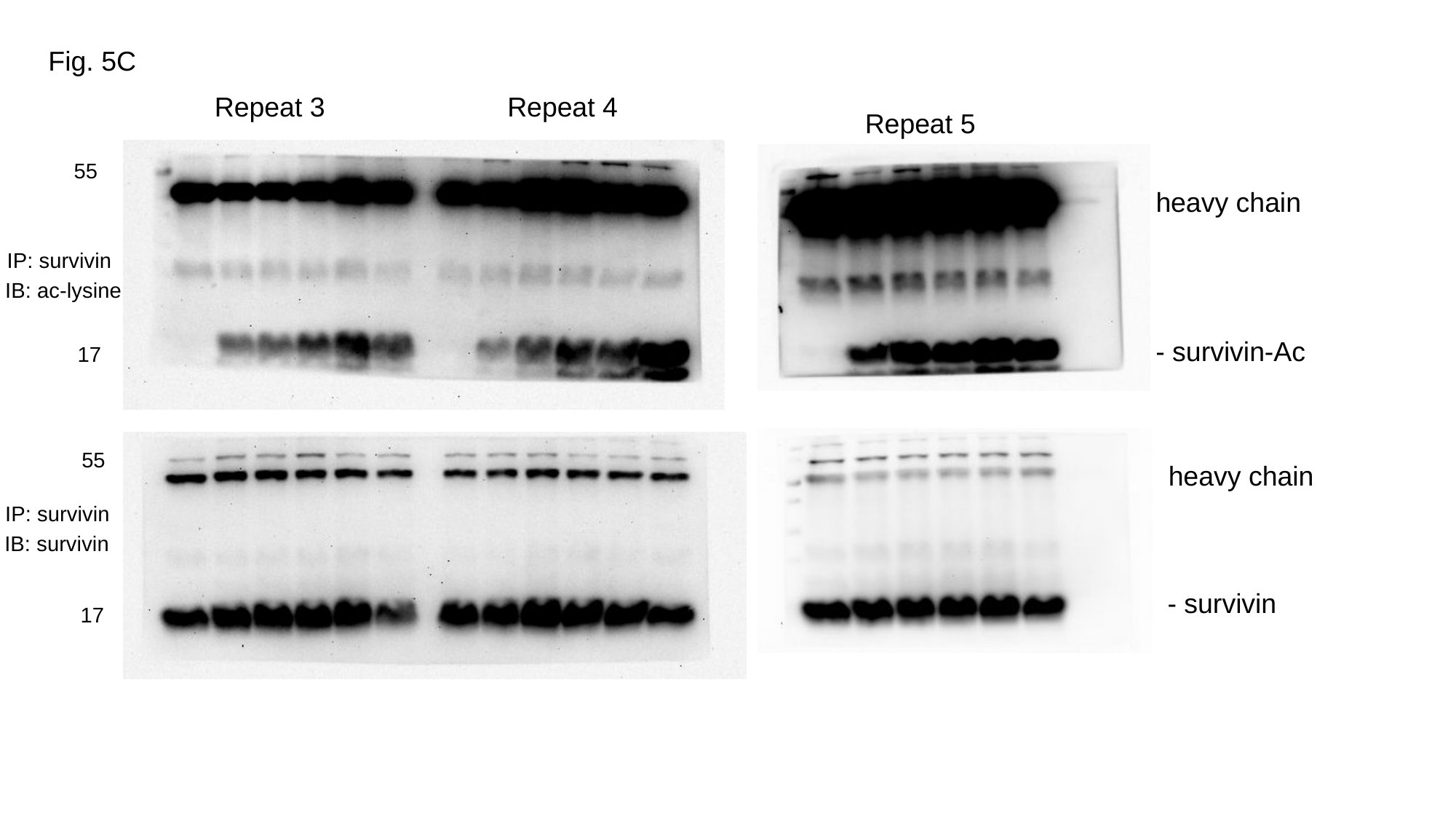

Fig. 5C
Repeat 3
Repeat 4
Repeat 5
55
heavy chain
IP: survivin
IB: ac-lysine
- survivin-Ac
17
55
heavy chain
IP: survivin
IB: survivin
- survivin
17

## Slide 29
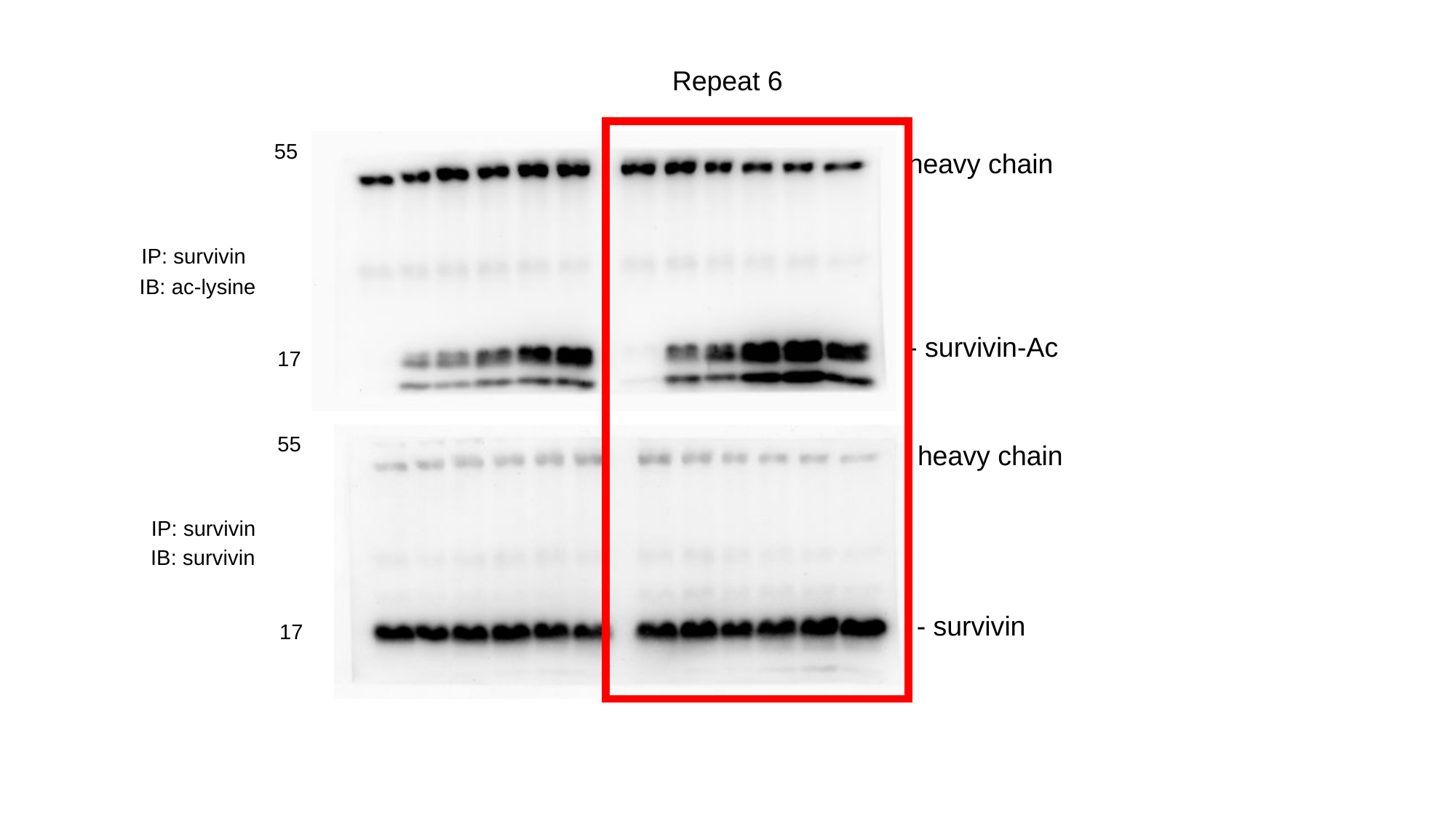

Repeat 6
55
heavy chain
IP: survivin
IB: ac-lysine
- survivin-Ac
17
55
heavy chain
IP: survivin
IB: survivin
- survivin
17

## Slide 30
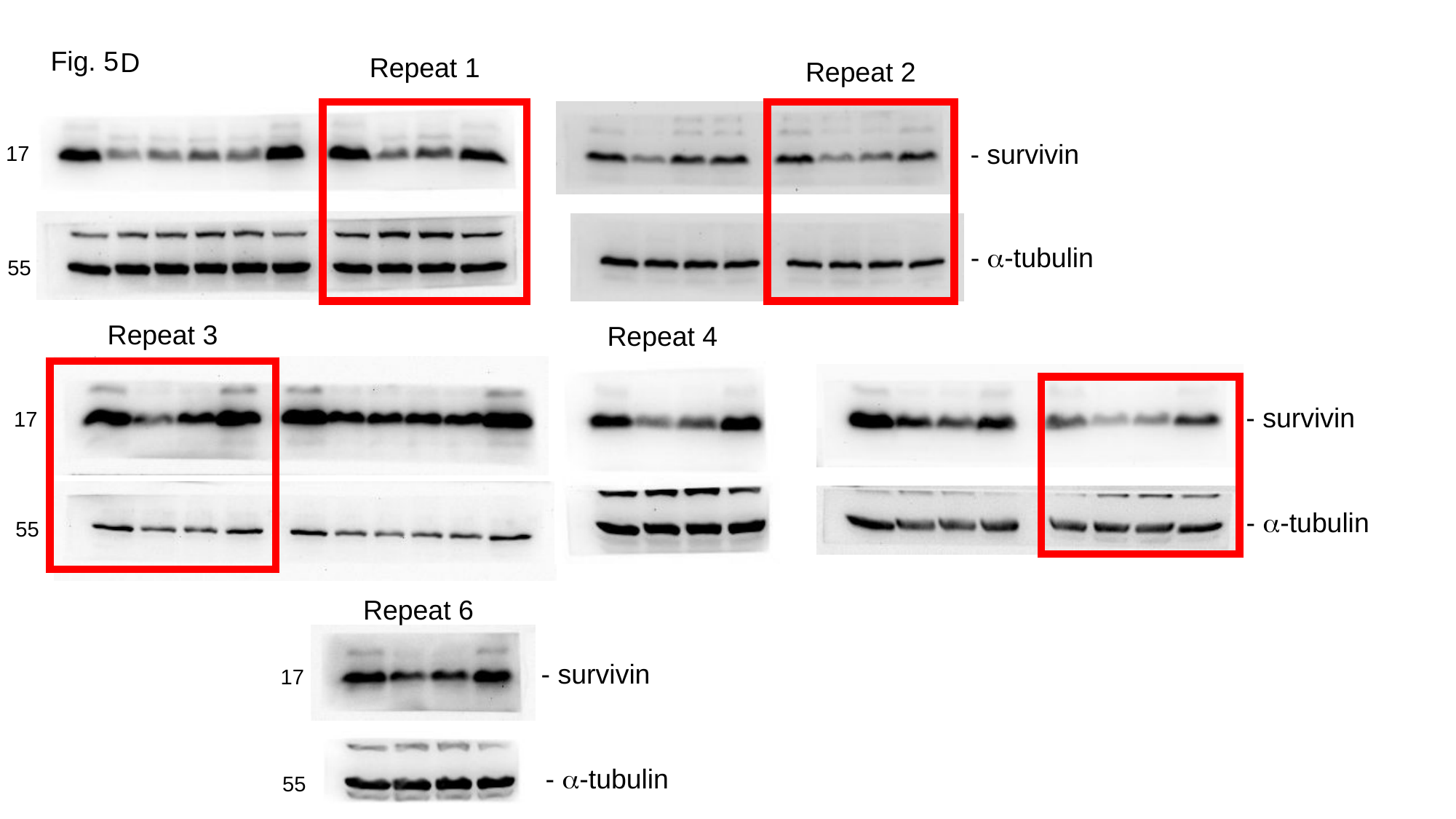

Fig. 5
D
Repeat 1
Repeat 2
- survivin
17
- a-tubulin
55
Repeat 3
Repeat 4
- survivin
17
- a-tubulin
55
Repeat 6
- survivin
17
- a-tubulin
55

## Slide 31
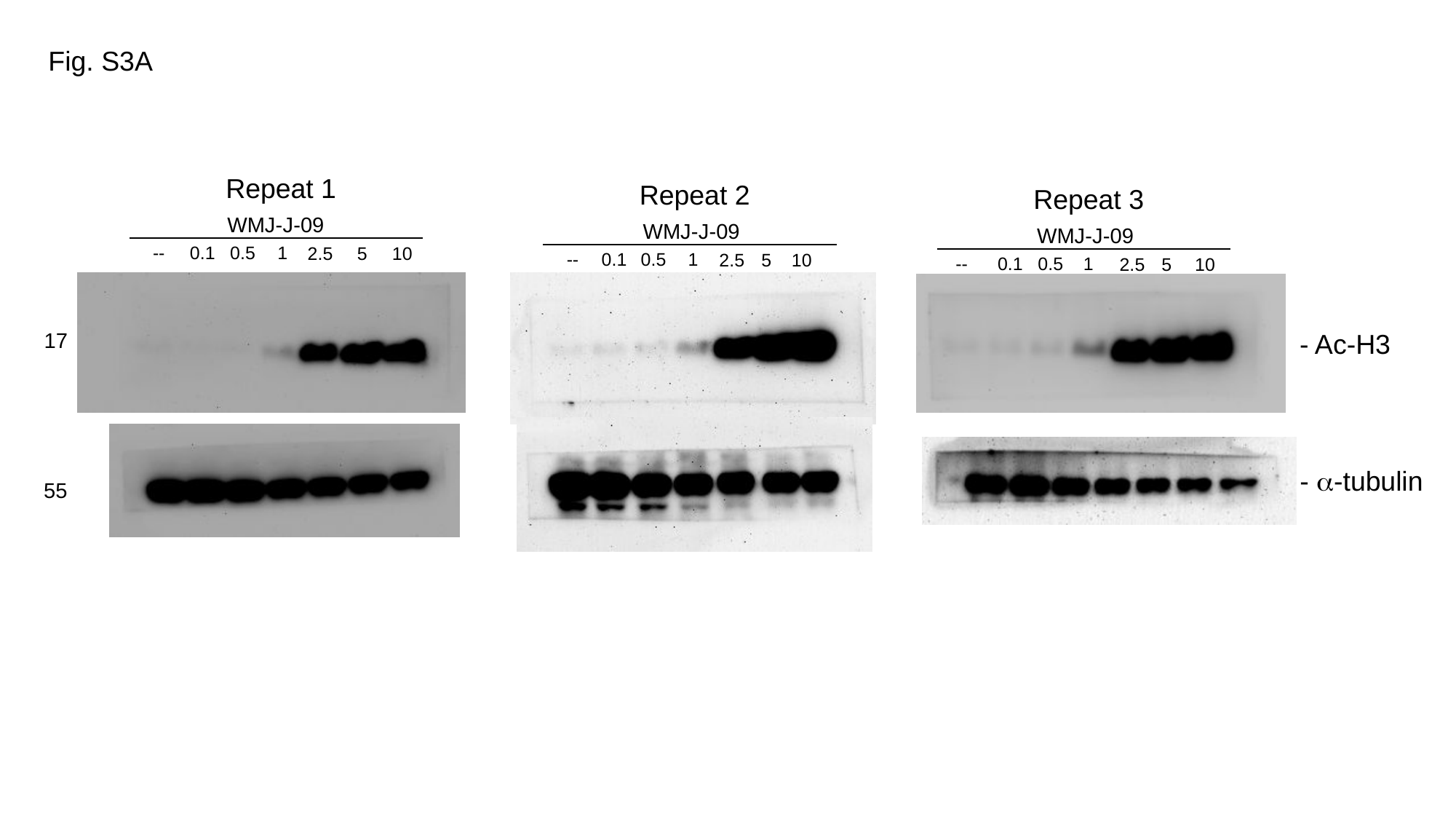

Fig. S3A
Repeat 1
Repeat 2
Repeat 3
WMJ-J-09
WMJ-J-09
WMJ-J-09
--
0.1
0.5
1
2.5
5
10
--
0.1
0.5
1
2.5
5
10
--
0.1
0.5
1
2.5
5
10
- Ac-H3
17
- a-tubulin
55

## Slide 32
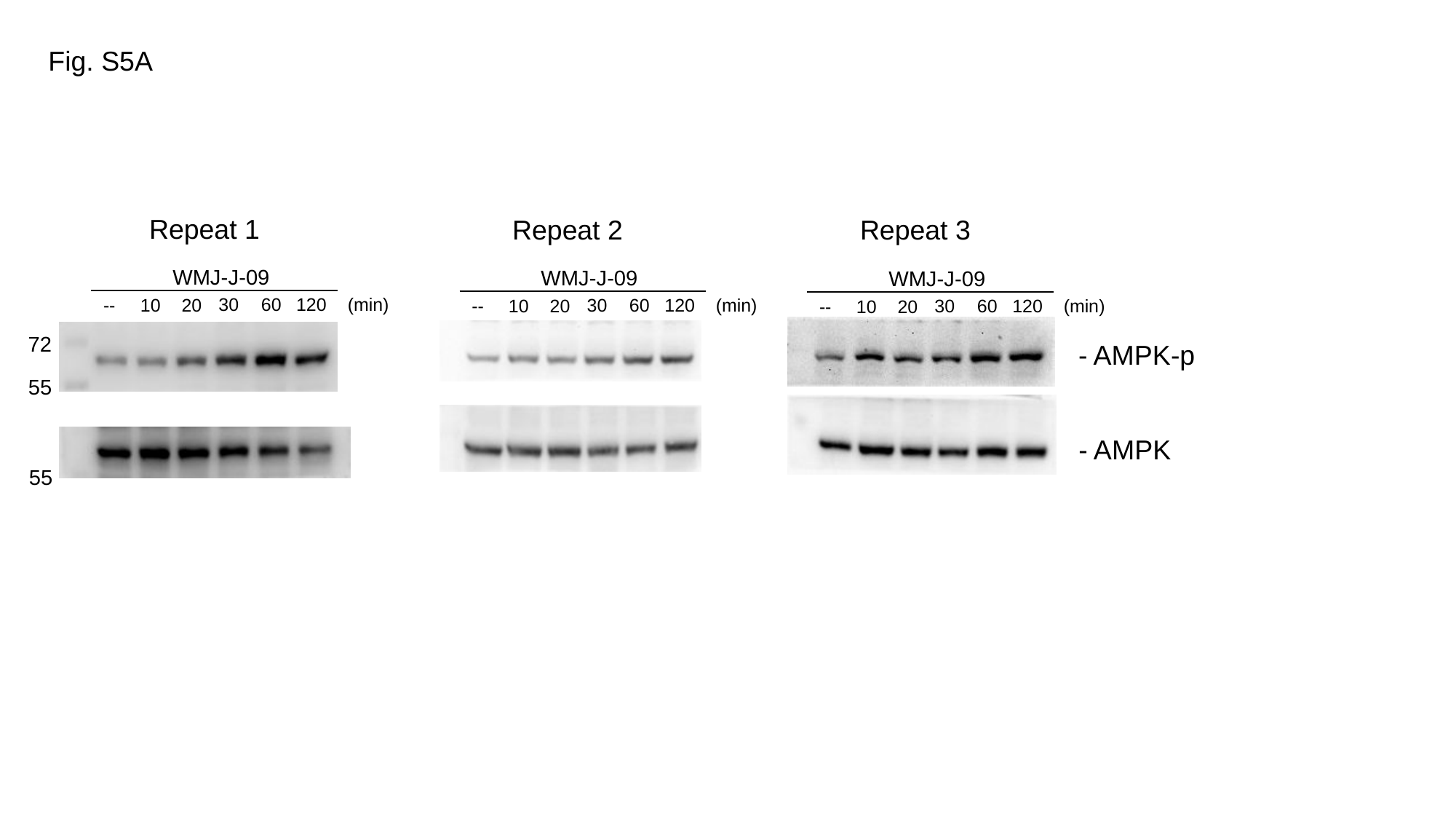

Fig. S5A
Repeat 1
Repeat 2
Repeat 3
WMJ-J-09
WMJ-J-09
WMJ-J-09
(min)
30
60
120
(min)
30
--
10
60
20
120
(min)
30
--
10
60
20
120
--
10
20
72
- AMPK-p
55
- AMPK
55

## Slide 33
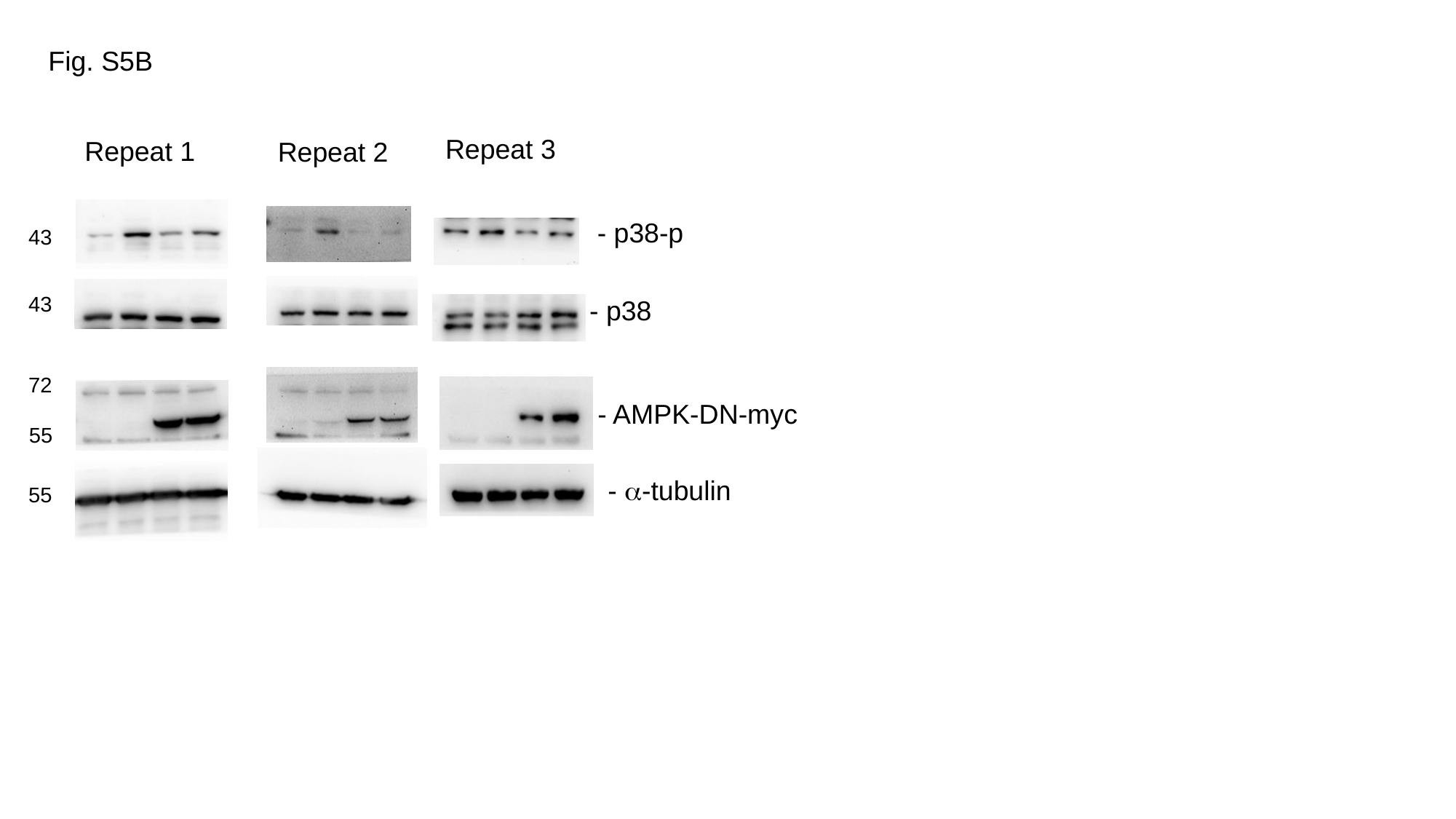

Fig. S5B
Repeat 3
Repeat 1
Repeat 2
- p38-p
43
43
- p38
72
- AMPK-DN-myc
55
- a-tubulin
55

## Slide 34
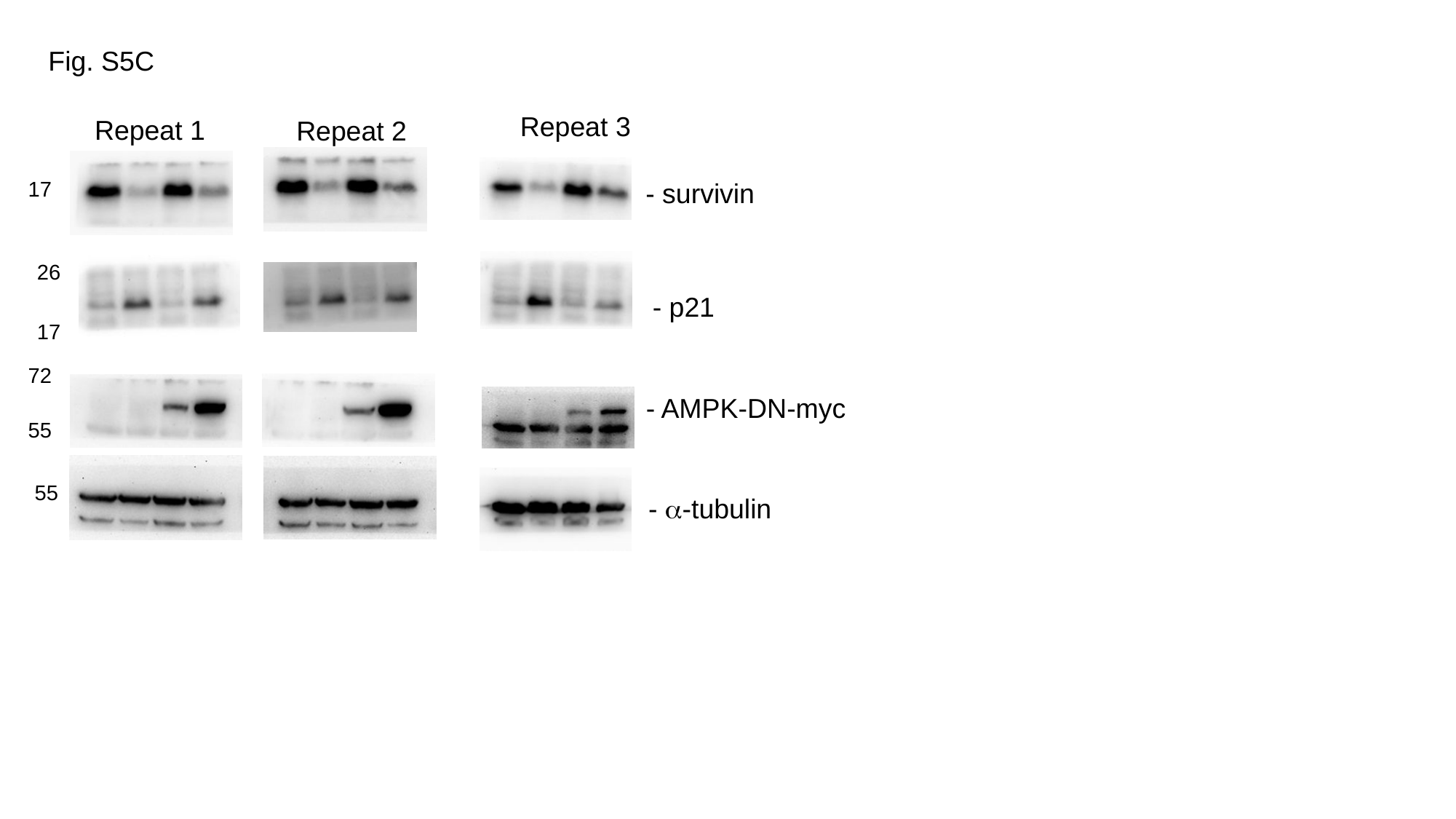

Fig. S5C
Repeat 3
Repeat 1
Repeat 2
17
- survivin
26
- p21
17
72
- AMPK-DN-myc
55
55
- a-tubulin
