# Supplementary material for: The hydroxamate based HDAC inhibitor WMJ-J-09 induces colorectal cancer cell death by targeting tubulin and downregulating survivin
Source: Sci Rep. 2025 Jun 4;15:19590. doi: 10.1038/s41598-025-04714-w (PMC12137728; doi:10.1038/s41598-025-04714-w)
Supplement: Supplementary file 2 — Supplementary Material 2 [file 41598_2025_4714_MOESM2_ESM.pdf]

Supplement Information

Supplement Methods

Materials

The antibody against AMPKα phosphorylated at Thr172 (T172) was purchased from Cell Signaling (Danvers, MA, USA). The antibody against AMPKα and myc tag were obtained from GeneTex Inc (Irvine, CA, USA). The myc tagged AMPK dominant negative mutant (AMPK-DN) was provided by Dr. Morris Birnbaum (HHMI, PA, USA).

Cell culture

The HCT116 cell line was kindly provided by Dr. Bert Vogelstein (Bunz et al., 1998). HCT-15, LoVo, and HT29 cell lines were obtained from the Bioresource Collection and Research Center (BBRC, Hsinchu, Taiwan). The FHC cell line, an epithelial cell line isolated from the large intestine of a healthy donor, from ATCC, was provided by Prof. Tsai-Tsen Liao (Graduate Institute of Medical Sciences, College of Medicine, Taipei Medical University, Taipei, Taiwan). The cells were maintained in McCoy's 5A (HCT116), RPMI1640 (HCT-15), Ham's F12 (LoVo), or DMEM (HT29) containing 10% FCS, 100 U/ml of penicillin G, and 100 μg/ml streptomycin in a humidified 37 °C incubator. The FHC cells were maintained in DMEM-F12 medium containing 25 mM HEPES, 0.005 mg/ml insulin, 0.005 mg/ml transferrin, 100 ng/ml hydrocortisone, 20 ng/ml human recombinant EGF, 10% FBS, 100 U/ml of penicillin G, and 100 μg/ml streptomycin in a humidified 37 °C incubator.

MTT assay

The colorimetric MTT assay was used to determine cell viability, as described previously (Chuang et al., 2017).

Supplement Figures

Supplement Fig. S1

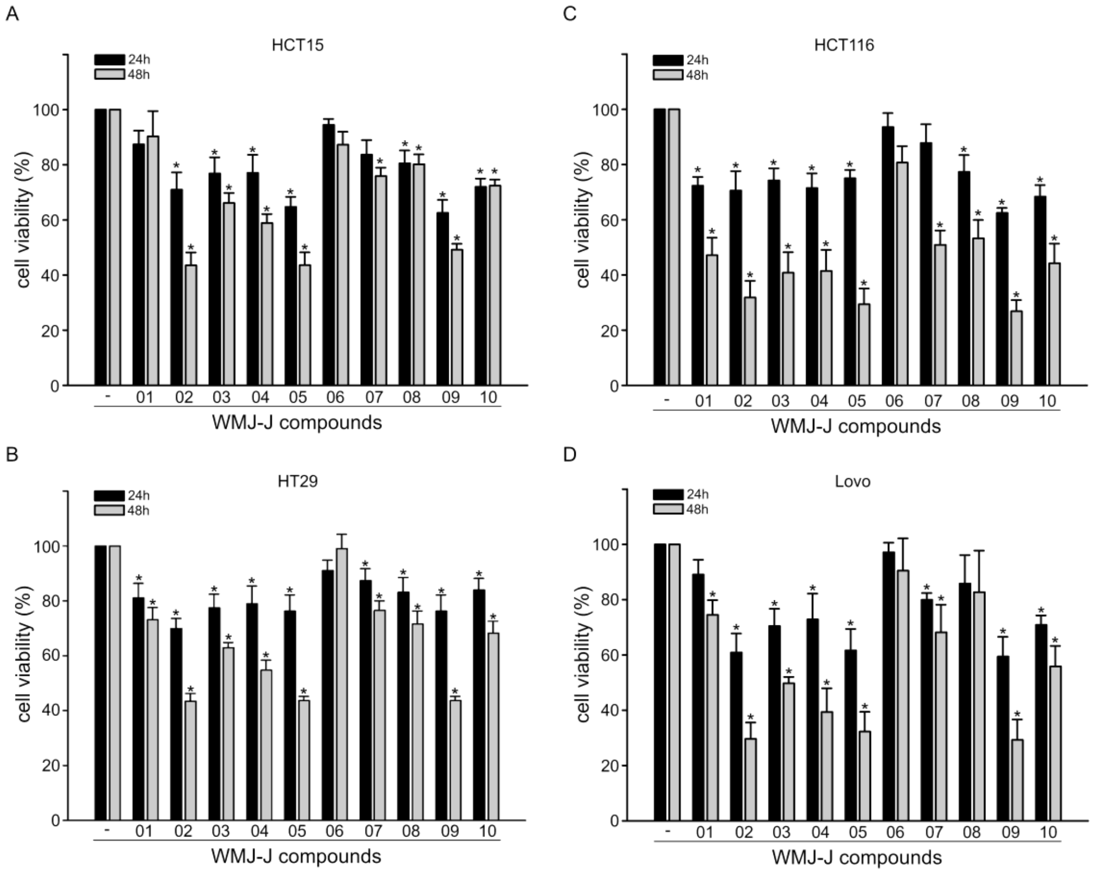

Supplement Fig. S2

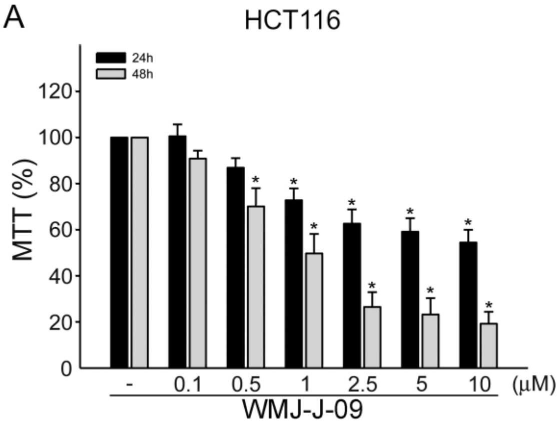

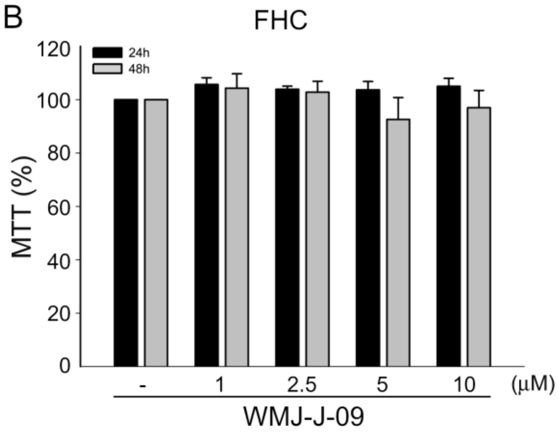

Supplement Fig. S3

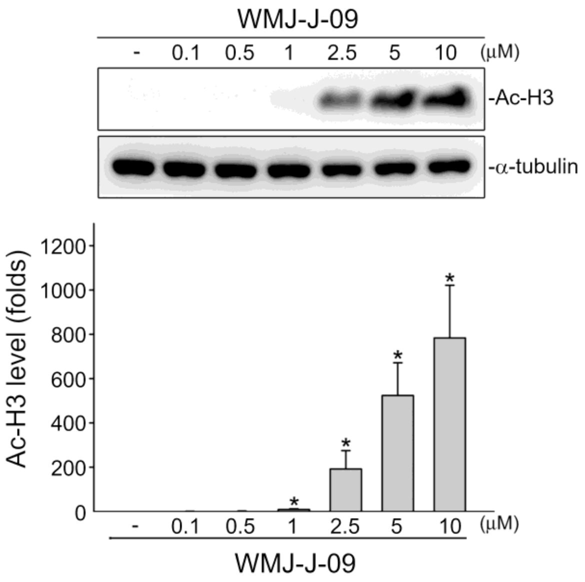

Supplement Fig. S4

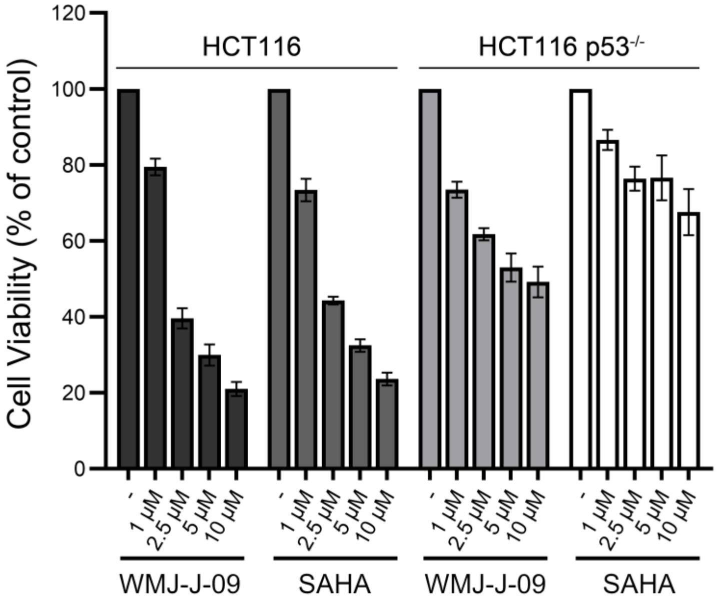

Supplement Fig. S5

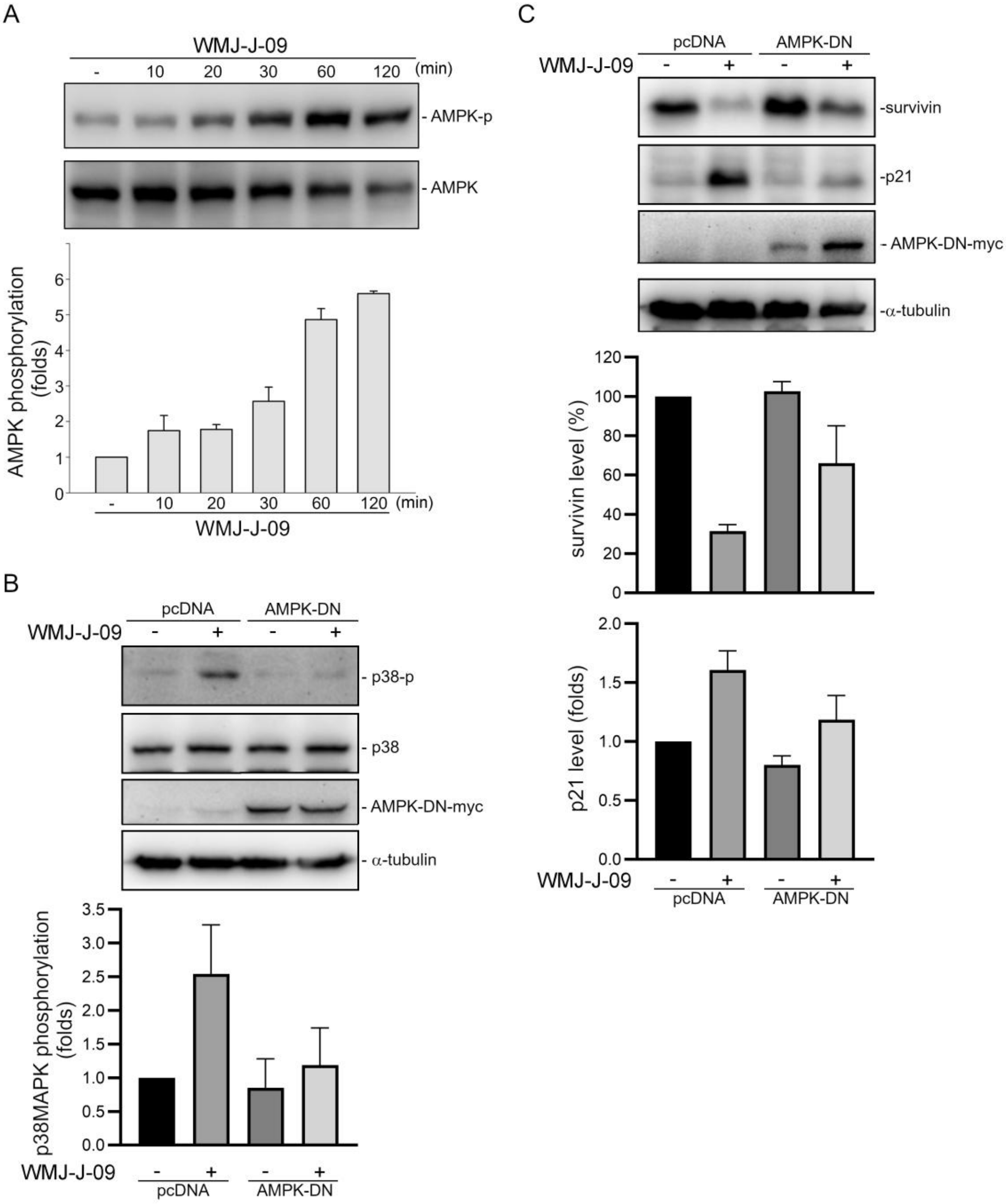

Supplement Fig. S6

A

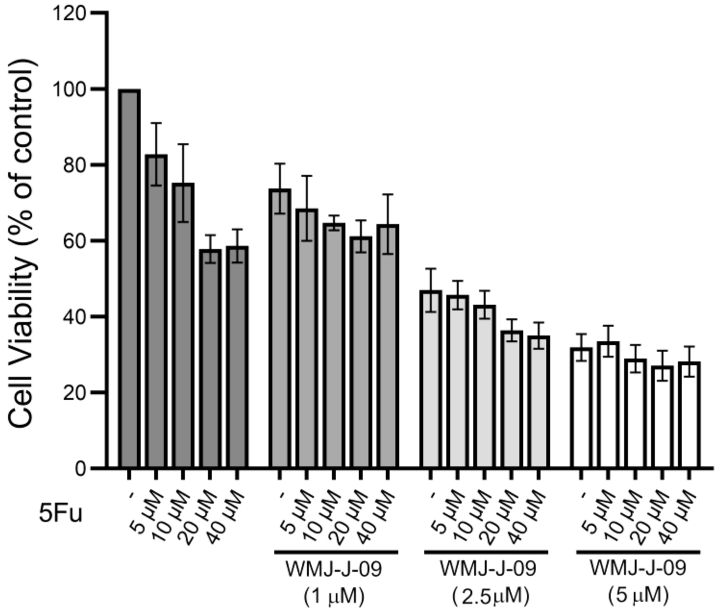

B

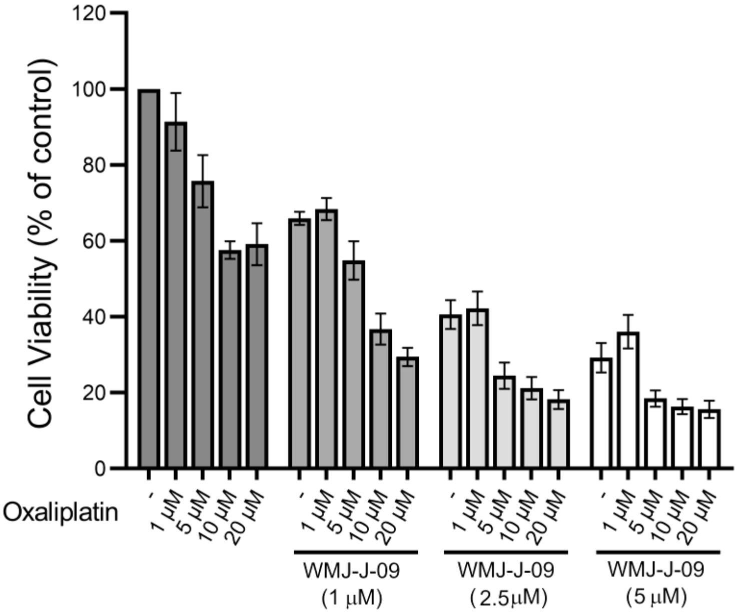

Supplement Figure legends

Fig. S1. Screen of WMJ-J compounds. (A-D) Cell viability of HCT15 cells (A), HT29 cells (B), HCT116 cells (C), and Lovo cells (D) after treating WMJ-J compounds for 24 and 48 h, measured by MTT assay. Error bars, mean ± S.E.M. (shown only for independent replicate experiments with n = 6). One-way ANOVA, with Tukey's post-hoc test, was used to assess statistical significance (compared to the control group, *$p < 0.05$).

Fig. S2. WMJ-J-09 reduced HCT116 cell viability in a time-dependent and concentration-dependent manner. Cell viability of HCT116 cells treated with WMJ-J-09 at indicated concentrations for 24h and 48h, measured by MTT assay. Error bars, mean ± S.E.M. (shown only for independent replicate experiments with n = 6). One-way ANOVA, with Tukey's post-hoc test, was used to assess statistical significance (compared to the control group, *$p < 0.05$).

Fig. S3. WMJ-J-09 enhanced histone 3 acetylation. Representative blot image shows the acetylation of histone 3 in HCT116 cells treated with WMJ-J-09 at indicated concentrations, measured by immunoblotting. The fold changes of histone 3 acetylation are calculated by quantifying band intensity. Error bars, mean ± S.E.M. (shown only for independent replicate experiments with n ≥ 3). One-way ANOVA, with Tukey's post-hoc test, was used to assess statistical significance (compared to the control group, *$p < 0.05$). Ac-H3, histone 3 acetylation

Fig. S4. The reducing effects of WMJ-J-09 and SAHA on cell viability in HCT116 and p53-deficient HCT116 cells. Cell viability of HCT116 or p53-deficient HCT116 (HCT116 $p53^{-/-}$) cells treated with WMJ-J-09 or SAHA at indicated concentrations for 48h, measured by MTT assay. Error bars, mean ± S.E.M. (shown only for independent replicate experiments with n = 4).

Fig. S5. AMPK contributes to WMJ-J-09-induced p38MAPK phosphorylation, surviving reduction, and p21 induction in HCT116 cells. (A) Representative blot image shows the AMPK phosphorylation in HCT116 cells treated with WMJ-J-09 for indicated time periods, measured by immunoblotting. The fold changes of AMPK phosphorylation are calculated by quantifying band intensity. Error bars, mean ± S.E.M. (shown only for independent replicate experiments with n = 3). Cells were transiently transfected for 48 h with pcDNA or AMPK-DN and then treated with WMJ-J-009 (2.5 μM) for another 30 min (B), or 24 h (C). The phosphorylation status

of p38MAPK (B), survivin, and p21 protein levels (C) were examined by immunoblotting. Compiled results are shown at the bottom of the chart. The extent of myc-tagged AMPK-DN was also determined by immunoblotting using anti-myc tag antibody. Each column represents the mean ± S.E.M. of three independent experiments.

Fig. S6. WMJ-J-09 enhanced the reducing effects of 5-fluorouracil (5-FU) or oxaliplatin on HCT116 cell viability. Cell viability of HCT116 cells treated with 5-FU or oxaliplatin in the absence or presence of WMJ-J-09 at indicated concentrations for 48h, measured by MTT assay. Error bars, mean ± S.E.M. (shown only for independent replicate experiments with n = 4).

References:

Bunz, F., Dutriaux, A., Lengauer, C., Waldman, T., Zhou, S., Brown, J. P., … Vogelstein, B. (1998). Requirement for p53 and p21 to sustain G2 arrest after DNA damage. *Science, 282*(5393), 1497-1501. doi:10.1126/science.282.5393.1497

Chuang, Y. F., Huang, S. W., Hsu, Y. F., Yu, M. C., Ou, G., Huang, W. J., & Hsu, M. J. (2017). WMJ-8-B, a novel hydroxamate derivative, induces MDA-MB-231 breast cancer cell death via the SHP-1-STAT3-survivin cascade. *Br J Pharmacol, 174*(17), 2941-2961. doi:10.1111/bph.13929
